# Silver Antibacterial Synergism Activities with Eight Other Metal(loid)-Based Antimicrobials against *Escherichia coli*, *Pseudomonas aeruginosa*, and *Staphylococcus aureus*

**DOI:** 10.3390/antibiotics9120853

**Published:** 2020-11-28

**Authors:** Ali Pormohammad, Raymond J. Turner

**Affiliations:** Department of Biological Sciences, Faculty of Science, University of Calgary, Calgary, AB T2N 1N4, Canada; ali.pormohammad@ucalgary.ca

**Keywords:** metal-based antimicrobials, antimicrobial synergies, gold, silver, copper, zinc, nickel, gallium, aluminum, selenite, tellurite, media, *Escherichia coli*, *Pseudomonas aeruginosa*, *Staphylococcus aureus*, bactericidal, bacteriostatic

## Abstract

The present study surveys potential antibacterial synergism effects of silver nitrate with eight other metal or metalloid-based antimicrobials (MBAs), including silver nitrate, copper (II) sulfate, gallium (III) nitrate, nickel sulfate, hydrogen tetrachloroaurate (III) trihydrate (gold), aluminum sulfate, sodium selenite, potassium tellurite, and zinc sulfate. Bacteriostatic and bactericidal susceptibility testing explored antibacterial synergism potency of 5760 combinations of MBAs against three bacteria (*Escherichia coli*, *Pseudomonas aeruginosa*, *Staphylococcus aureus*) in three different media. Silver nitrate in combination with potassium tellurite, zinc sulfate, and tetrachloroaurate trihydrate had remarkable bactericidal and bacteriostatic synergism effects. Synergism properties of MBAs decreased effective antibacterial concentrations remarkably and bacterial cell count decreased by 8.72 log10 colony-forming units (CFU)/mL in *E. coli*, 9.8 log10 CFU/mL in *S. aureus*, and 12.3 log10 CFU/mL in *P. aeruginosa*, compared to each MBA alone. Furthermore, most of the MBA combinations inhibited the recovery of bacteria; for instance, the combination of silver nitrate–tetrachloroaurate against *P. aeruginosa* inhibited the recovery of bacteria, while three-fold higher concentration of silver nitrate and two-fold higher concentration of tetrachloroaurate were required for inhibition of recovery when used individually. Overall, higher synergism was typically obtained in simulated wound fluid (SWF) rather than laboratory media. Unexpectedly, the combination of A silver nitrate–potassium tellurite had antagonistic bacteriostatic effects in Luria broth (LB) media for all three strains, while the combination of silver nitrate–potassium tellurite had the highest bacteriostatic and bactericidal synergism in SWF. Here, we identify the most effective antibacterial MBAs formulated against each of the Gram-positive and Gram-negative pathogen indicator strains.

## 1. Introduction

The increase in antibiotic(s) resistance in bacteria is a growing threat to global human health that has led to what is now referred to as the antimicrobial resistance (AMR) era [1]. AMR has led to an urgent call for antimicrobial stewardship as well as a call for alternative antibacterial treatment(s). Metals, metalloids, and halogen ions and alloys have seen historic use in infection control and might be an appropriate option for treatment of AMR isolates in modern times [2,3]. A comprehensive review by our research group overviewed metals antimicrobial history, molecular mechanisms, and antimicrobial potency of these components [2]. There are now many studies reporting the antibacterial potency of metals; moreover, certain metals have demonstrated a remarkable efficacy against both planktonic and biofilm forms of bacteria [4,5,6,7]. Additionally, several metals have strong antibacterial activity against multidrug resistance isolates [5,8,9]. Metal(loid)s have also been found to have synergistic antibacterial and antibiofilm activity in combination with antiseptics [10], biosurfactants [11] and other antibiotics [12,13].

Silver (Ag) is the most well-known and studied metal(loid)-based antimicrobials (MBA), and applications of Ag as an antimicrobial agent can be dated back to ancient civilizations, including the Phoenicians, Greeks, Egyptians and Romans [14]. Silver as an MBA is now commercially available and widely used in industry, agriculture and healthcare. Such MBAs now have a wide variety of applications in personal care products; wound dressings; detergents; and indwelling medical devices, such as artificial joints, catheters and implants [15,16]. Silver shows broad-spectrum antibacterial and antimicrobial potency likely due to the multiple cellular targets giving pleiotropic killing effects [17], with multiple genes involved in tolerance and sensitivity response [18]. Thus, Ag exhibits a huge capability to overcome AMR and participate in infection control [17,19]. However, resistance against Ag in clinical isolates is increasing [20,21]. Bacteria may acquire resistance against Ag and other antimicrobial agents via the acquisition of resistance genes through horizontal gene transfer or genome mutation [21,22]. One of the best strategies for eradication and prevention of AMR is combination therapy [23]. For instance, if the independent chances of resistance to antibiotic A and B are 1 × 10^5^ and 1 × 10^6^, respectively, then the possibility of spontaneous resistance to the A + B combination will be 1 × 10^11^ or the product of the two rates [23,24]. Moreover, if the agents have synergism effects, a lower dosage of the antimicrobials is required when using them as a combination treatment in comparison with using each antimicrobial alone [10]. Additionally, using MBAs in synergism combinations would lead to decreasing the effective concentration and decreasing antibiotic side effects (10).

In the present study, we explored the potential antibacterial synergism effects of silver nitrate with eight other MBAs. We developed an effective technique to evaluate both bacteriostatic and bactericidal susceptibility of MBA combinations. The combinations of Ag with other MBA concentrations were evaluated against three indicator strains of pathogens, namely, *Escherichia coli*, *Pseudomonas aeruginosa*, and *Staphylococcus aureus*. We also evaluated the synergistic combinations in three different media, as our experience has shown that antimicrobial efficacy is considerably different in different media due to physiological fitness and MBA speciation differences. This study concludes by providing the most effective antibacterial formulations of MBA combinations against both the Gram-positive and Gram-negative candidate bacteria.

## 2. Result

### 2.1. Bacteriostatic and Bactericidal Potency of Various Metal(loid) Salts

Bactericidal and bacteriostatic efficacy of various metal salts was obtained for the three strains in three different media, Luria broth (LB), Mueller–Hinton broth (MHB) and simulated wound fluid (SWF). As bacteria behave differently in different nutrition and growth conditions, it is important to evaluate stress tolerances under various media in order to capture physiological differences in lab media closer to actual infection conditions. In this study, MBAs are chosen from a spectrum of transition elements and metalloids shown previously to have potential as antimicrobial agents. The metal(loid) salts of silver nitrate (AgNO_3_, abbreviated to **Ag**) copper (II) sulfate (CuSO_4_, **Cu**), gallium (III) nitrate ((Ga(NO_3_)_3_, **Ga**), nickel sulfate (NiSO_4_, **Ni**), tetrachloroaurate (III) (AuCl_4_, **Au**), aluminum sulfate (Al_2_(SO_4_)_3_, **Al**), sodium selenite (Na_2_SeO_3_, **Se**), potassium tellurite (K_2_TeO_3_, **Te**), and zinc sulfate (ZnSO_4_, **Zn**), were explored. For all Figures and texts, the metal(loid) salts regardless of speciation of element ion in solution are abbreviated to the elemental symbol for convenience to the reader. As expected, the different bacteria in different media showed different tolerance outcomes from MBA challenges.

Table 1 shows the minimum inhibitory concentration (MIC) and minimum bactericidal concentration (MBC) of nine MBAs for *P. aeruginosa*, *S. aureus*, and *E. coli*. The MIC values reflect the bacteriostatic activity of the MBAs. The data show that for several of the MBAs investigated, there were differences in the MICs obtained between media conditions. Further, the media effect was different for different MBAs against different strains. Ag with MIC of 0.015 mM in MHB (value ranges are given in Table 1 in parenthesis) and Te with the same MIC in LB media had the lowest MIC for *E. coli*. For *S. aureus*, the lowest MIC was Ag 0.03 mM in MHB, and for *P. aeruginosa*, it was Te at 0.015 mM in LB media. Te had an expected higher MIC for *S. aureus* (6.25 mM in MHB, 0.2 mM in LB, and 50< in SWF) in comparison with *P. aeruginosa* (0.06 mM in MHB, 0.015 in LB, and 6.25 mM in SWF) and *E. coli* (0.25 mM in MHB, 0.015 in LB, and 0.25 mM in SWF). The MIC of Te experiments was repeated seven times to validate trends of MIC for the three different bacteria.

Bactericidal efficacy of the MBAs towards the three strains was also reflected in their MBC values (Table 1). Similar to MIC, the MBA values also ranged by media type and strain studied. Between nine MBAs, Ag with MBC of 0.125 mM in MHB and Te with MBC of 0.125 mM in LB had the lowest MBC for *E. coli*. The lowest MBC for *S. aureus* was Te 0.2 mM in LB, and for *P. aeruginosa*, Te with MIC of 0.125 mM in MHB media. Generally, the MIC/MBC decreased with increasing complexity of the media, although this is not true with all metals for all strains.

Overall, Te (except *S. aureus*) and Ag gave the lowest MIC and MBC between the nine MBAs for all three bacteria in the three different culture media (Table 1). 

### 2.2. Synergistic Bacteriostatic and Bactericidal Activity of Ag in Combinations with Other Metal-Based Antimicrobials

In this binary screening process, we evaluated the synergism effects of a total of 5760 combinations of MBA concentrations, where Ag was systematically paired with each of the other eight MBAs against the three pathogen indicator strains in three different media. Figures S1–S52 (in Supplementary Materials) give the synergism patterns of both bacteriostatic and bactericidal as well as the fractional inhibitory concentration (FIC) values for combinations of Ag with eight MBAs against *P. aeruginosa*, *S. aureus*, and *E. coli*. For simplicity, these results were categorized by tested bacteria, media, MBA combinations, and synergism FIC values. the lowest FIC (highest synergism combinations) were collected and ranked, and are reported in Table 2.

**Figure 1:**
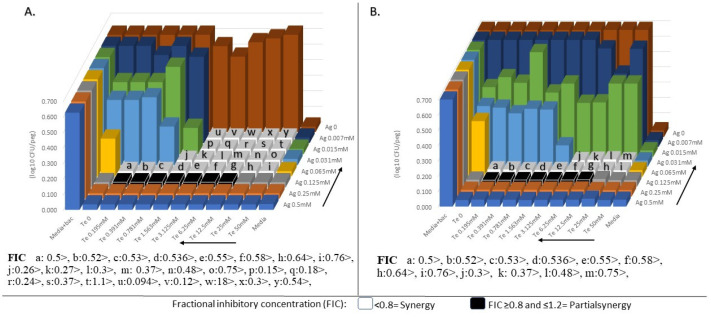
(**A**) Minimum inhibitory concentration (MIC) and (**B**) minimum bactericidal concentration (MBC) synergism (syn) effects of silver nitrate (AgNO_3_) with potassium tellurite (K_2_TeO_3_) combination against *S. aureus* in simulated wound fluid (SWF). Combination of Ag–Te (0.007 mM of Ag + 3 mM of Te) was the lowest fractional inhibitory concentration (FIC < 0.094) and the highest bacteriostatic synergism effect between all metal-based antibiotics combinations.

From Table 2, we see the lowest bacteriostatic FICs (the highest synergism effect) for *E. coli* was the combination of Ag–Zn (0.031 mM Ag + 0.25 mM Zn) (Figure S10) and Ag–Au (0.007 mM Ag + 0.062 mM Au) (Figure S27) both with FIC of 0.37 obtained in LB media. For *S. aureus*, the combination of Ag–Te (0.007 mM Ag + 3 mM Te) was the lowest bacteriostatic FIC (0.094) in SWF (Figure 1). For *P. aeruginosa*, the lowest bacteriostatic FIC (0.3) was the combination of Ag–Te (0.007 mM Ag + 0.125 mM Te) in SWF (Figure S24).

**Figure 2:**
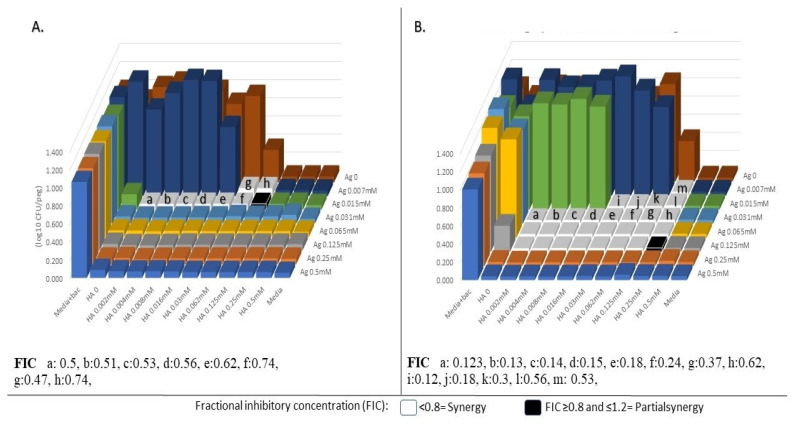
(**A**) Minimum inhibitory concentration (MIC) and (**B**) minimum bactericidal concentration (MBC) synergism (syn) effects of silver nitrate (AgNO_3_) with hydrogen tetrachloroaurate trihydrate (HA, AuCl_4_•3H_2_O) combination against *P. aeruginosa* ATCC 27853 in Mueller–Hinton broth (MHB). Combination of Ag–Au (0.015 Ag + 0.03 Au) was the lowest fractional inhibitory concentration (FIC = 0.12) and the highest bactericidal synergism effect between all metal-based antibiotics combinations.

The lowest bactericidal fractional bactericidal concentration (FBC) (the highest synergism effect) obtained for *E. coli* was the combination of Ag–Zn (0.015 mM Ag + 4 mM Zn) with 0.31 FIC in MHB (Figure S11); for *S. aureus*, the combination of Ag–Zn (0.125 mM Ag + 1 mM Zn) was lowest bactericidal (FBC = 0.25) in SWF (Figure S18); for *P. aeruginosa*, the lowest bactericidal (FBC = 0.12) was the combination of Ag–Au (0.015 Ag + 0.03 Au) in MHB (Figure 2).

Out of the six highest bacteriostatic and bactericidal synergism combinations, three of them shows the higher synergism of Ag in combination with MBAs in SWF rather than LB and MHB.

### 2.3. Comparison of Bacteriostatic and Bactericidal Synergism Effects of Metal(loid)-Based Antimicrobials 

A comparison of bacteriostatic and bactericidal synergism effects of MBAs is shown in Figure 1 and Figure 2, and Figures S1–S52. Out of 72 MBA combination tests, 66 tests had higher bactericidal synergism effects rather than bacteriostatic, which demonstrates the huge potential of exploring Ag–MBA combinations as biocides and antiseptics. For example, the combination of Ag–Au against *P. aeruginosa* in MHB had bactericidal FBC = 0.12 and bacteriostatic FIC = 0.47, which shows that combinations of Ag–Au had a higher bactericidal synergism effect rather than the bacteriostatic effect (Figure 2). Moreover, Figure 2B shows that synergism effect of Ag–Au remarkably reduced the effective bactericidal concentration of each agent (four-fold), which means that the combination of Ag–Au has bactericidal effects at a concentration four-fold lower than each agent alone.

The eight MBA combinations that had s higher bacteriostatic synergism effect rather than bactericidal are as follows: Ag–Zn in LB-*E. coli*, Ag–Te in SWF-*S. aureus*, Ag–Ni in SWF-*E. coli*, Ag–Ni in SWF-*P. aeruginosa*, Ag–Ni in LB-*S. aureus*, Ag–Ni in MHB-*S. aureus*, Ag–Se in SWF-*E. coli*, and Ag–Zn in LB-*P. aeruginosa*.

### 2.4. Antagonistic Activity of Ag in Combinations with other Metal(loid)-Based Antimicrobials

Out of 5760 combinations of Ag–MBAs, just seven of them had antagonistic effects. Interestingly, the combination of Ag–Te had antagonistic bacteriostatic effects in LB vs. synergy in SWF against all three strains (Table 1). Therefore, these data highlight a cautionary tale of concluding antimicrobial efficacies using laboratory media compared to those reflecting infection conditions. Another combination that had antagonistic bacteriostatic effects was the combination of Ag–Al in both LB and MHB. Moreover, Ag–Al had antagonistic bactericidal effects against *P. aeruginosa* and *S. aureus* in SWF (Table 1). When it comes to metal toxicity, the speciation state is critical. In such complex media, we expect the metal ions to be complexed with media molecules and in equilibrium between many different species. With two metals present, the number of species and interacting species becomes even more complex. We also recognize that with two metals present, the metals may be acting independently (i.e., different biochemical cell sites) or together (both metals hitting the same biochemical site). Regardless of this complexity, clearly, some mixtures lead to remarkable antimicrobial outcomes.

### 2.5. Recovery Potency of Bacteria after Exposure to the Combinations of Metal(loid)-Based Antimicrobial

**Figure 3:**
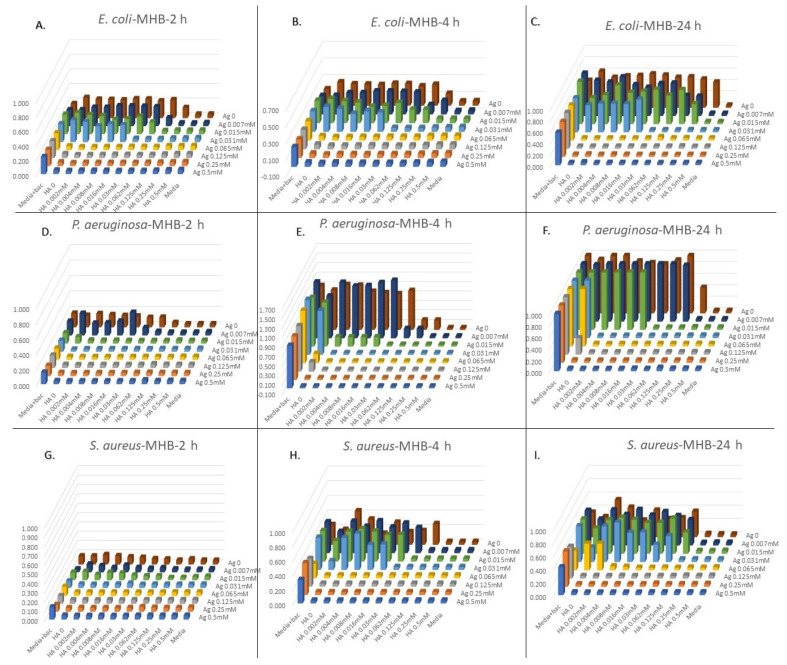
Recovery potency of *E. coli* against synergism effects of silver nitrate (AgNO_3_) with tetrachloroaurate trihydrate (AuCl_4_•3H_2_O) combination after 2 h (**A**), 4 h (**B**), and 24 h (**C**), *P. aeruginosa* after 2 h (**D**), 4 h (**E**), and 24 h (**F**), and *S. aureus* after 2 h (**G**), 4 h (**H**), and 24 h (**I**) in Mueller–Hinton broth (MHB). Combination of Ag–Au against *P. aeruginosa* in MHB inhibited the recovery of bacteria in the same fresh media, while Ag and Au without combination could not inhibit the recovery of bacteria, and the bacteria recovered in three-fold higher dilution of Ag and two-fold higher dilution of Au in fresh media.

One of the biggest clinical and industrial problems with controlling and treatment of bacterial infections is the strong recovery potency of some bacteria after exposing them to some antibiotics, i.e., when the antibiotic concentration is diluted. This is due to the antimicrobial being bacteriostatic vs. bactericidal. Therefore, recovery potency of three indicator strains was surveyed from the initial synergism screen. Figures S53–S69 show the recovery potency of *P. aeruginosa*, *S. aureus*, and *E. coli* in three different media after 2, 4, and 24 h. A promising outcome was the combination of Ag with some MBAs inhibited the recovery potency of these strains when transferred and cultured in fresh media. For instance, the combination of Ag–Au against *P. aeruginosa* in MHB inhibited the recovery of bacteria in the same fresh media, while the Ag and Au without combination could not inhibit the recovery of bacteria, and the bacteria recovered in three-fold higher dilution of Ag and two-fold higher dilution of Au when we used them alone. This and other combinations with high recovery potency in different media are shown in Figure 3.

### 2.6. Synergism Exposure Growth Curve Assays

**Figure 4:**
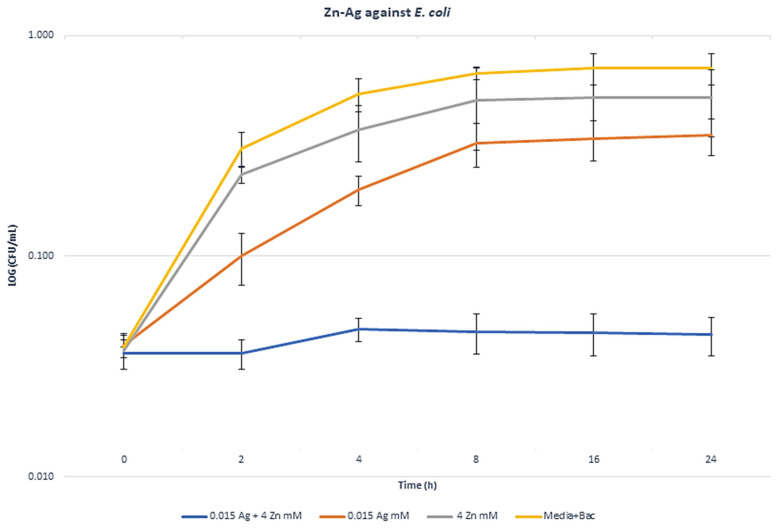
Growth curves in the presence of MBA combinations and the synergism effect of Ag–Zn against *E. coli* cultured in Mueller–Hinton broth.

**Figure 5:**
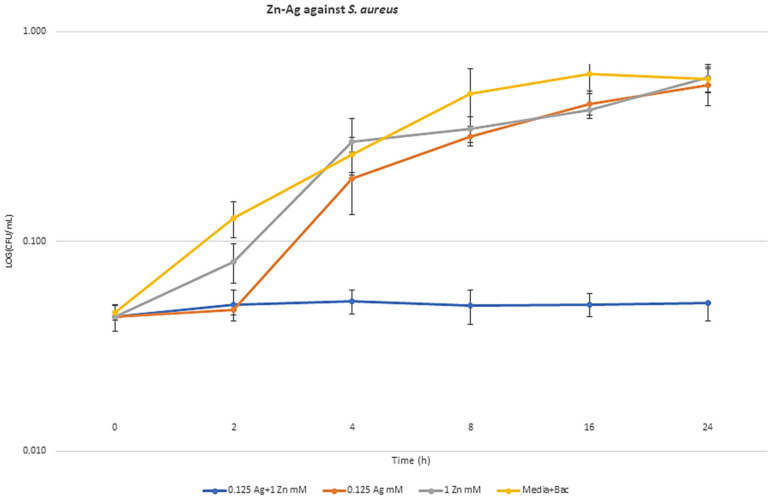
Growth curves in the presence of MBA combinations and the synergism effect of Ag–Zn against *S. aureus* cultured in simulated wound fluid.

**Figure 6:**
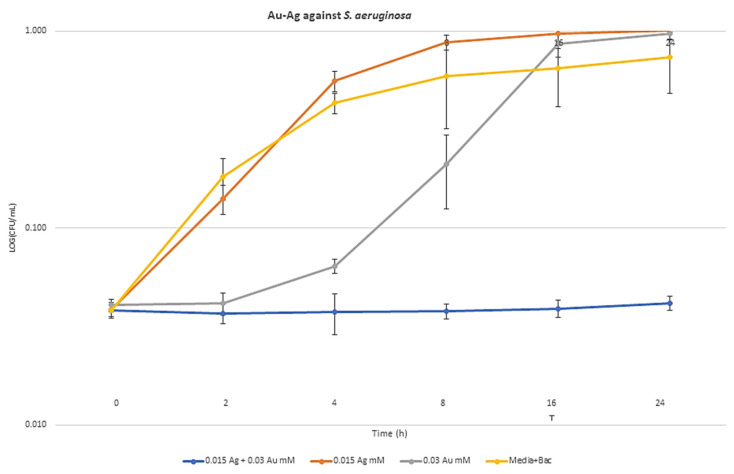
Growth curves in the presence of MBA combinations and the synergism effect of Ag–Au against *P. aeruginosa* cultured in Mueller–Hinton broth.

The highest synergism bactericidal MBA components against *E. coli* (0.015 mM Ag + 4 mM Zn), *S. aureus* (0.125 mM Ag + 1 mM Zn), and *P. aeruginosa* (0.015 mM Ag + 0.03 mM Au) were selected for metal exposed growth curves. Through the time-based bactericidal experiment, the growth inhibition abilities of selected MBA combinations compared to a single dosage of each MBAs were measured against the three strains. The colony count of the metal combination data for all three bacteria was significantly lower than that of the single metal data (*p* < 0.05). Bacterial cell count decreased by 8.72 log10 colony-forming units (CFU)/mL in *E. coli* (Figure 4), 9.8 log10 CFU/mL in *S. aureus* (Figure 5), and 12.3 log10 CFU/mL in *P. aeruginosa* (Figure 6), compared to each MBA alone at 24 h. The curves in Figure 4, Figure 5 and Figure 6 show the remarkable ability of combination treatment to control the growth of these strains under planktonic conditions.

## 3. Discussion

The main aim of the present study is identifying if silvers’ antimicrobial efficacy can be enhanced through the addition of a second MBA. This led to finding the best antibacterial combinations between silver with other MBAs in appropriate ratios to enhance its antimicrobial efficacy. The actual molecular mechanism of the synergies observed was not investigated here. Our data set showed that Ag has strong synergism in combination with the number of other MBAs. In our study, we first evaluated both bactericidal and bacteriostatic efficacy of the nine MBAs, which was determined for *P. aeruginosa*, *S. aureus*, and *E. coli* in three different culture media. Then, the synergism effects of Ag with the eight other MBAs were explored.

As bacteria have different physiological fitness when growing under different carbon, nutritional (nitrogen, phosphate, sulfur amino acids, etc.) and energy sources [25,26], we were also curious to see if there would be differences between a defined (MHB) to a more complex (LB) laboratory media compared with an extremely complex media more closely representing an actual infection environment (SWF). Typically, the use of a medium with a defined composition allows researchers to probe the specific effects of nutrients on bacteria growth, antibacterial susceptibility pattern, and recovery potency of bacteria after exposure with a specific antibiotic [26,27]. The different media would give rise to different fitness states of the bacteria, but also give different metal speciation states (redox, ionization, and chelation forms), which can change bioavailability and toxicity. It would be very complex to determine all the species present and which one is giving rise to the antimicrobial effect in each of the media. In fact, it could even be a different species for each strain in each media, as each could secrete different excreted metabolites and other biomolecules. Thus, as expected, the bacteria in different media had different antibacterial and synergism effects, which may be a result of speciation of differential physiological fitness.

Another key variable that is often overlooked is the strain of the species used in the antimicrobial testing. Most microbiologists now recognize issues around domestication of lab strains, where the same strain in different labs can behave differently due to repetitive culture passages. Thus, the antimicrobial literature is often difficult to compare, in part also from different antimicrobial assays used. Additionally, since organic-based antibiotics have a different effect on different bacterial species, especially between Gram-negative and Gram-positive bacteria [12,28], we wanted to make sure we sampled key indicator strains from each group. Therefore, we analyzed synergism potency of silver with other MBAs using the American Type Culture Collection (ATCC)pathogen indicator strains of both Gram-negative (*E. coli* and *P. aeruginosa*) and Gram-positive (*S. aureus*) bacteria in all three media. To avoid the stated domestication issues, the strains obtained from ATCC were cultured to produce the lab stock, and all culturing for assays began from these primary stocks.

Briefly, our results showed that Te and Ag were the most antibacterial of the nine MBAs towards all three bacteria in the three different culture media. Ag is well-known and one of the first known metals reported to be used as an antimicrobial agent [14,29]. However, our results showed that Te in the form of tellurite (most likely species: HTeO_3_^−^) gives a lower MIC and MBC in comparison with Ag in many conditions, and that it is especially effective for the Gram-negative bacteria (*E. coli* and *P. aeruginosa*) compared to Gram-positive bacteria. Different cell wall structures in Gram-negative and Gram-positive bacteria might be a possible reason for this difference by affecting uptake as well as the reactivity of Te to glutathione versus mycothiol [30,31]. This feature may lead to lower toxicity and side effects of Te for eukaryotic cells and humans. A study by Pugin et al. used -SH and -OH functional group containing compounds for enhancing Te toxicity against Gram-positive bacteria, especially *S. aureus* [32], perhaps influencing Te uptake or the speciation. Despite the wealth of Te antimicrobial activity, there has been less exploration in regard to specific applications of Te as an antimicrobial in industry and clinical approaches, or in synergism with other antibiotics [30,31,33]. Although Se and Te have closely related properties, under the conditions studied here, we observed no antimicrobial activity from selenite.

Previous studies showed significant antibacterial synergism potency of Ag with other antibiotics against bacterial infection, especially for multidrug resistance bacteria [12,13,34,35]. In the present study, antibacterial synergism potency of 5760 concentration combinations of Ag with MBAs against three indicator strains showed that Ag in combination with Te, Zn, and Au has excellent bactericidal and bacteriostatic synergism. The benefit of strong synergism effects of MBAs is that it decreases the effective antibacterial concentration remarkably. Our research group’s previous studies showed antibacterial and antibiofilm potency of MBAs [2,3,36,37]. However, there is a limitation on the selection of different metal ions one can use due to the possible host cell toxicity of certain metals [38,39] such as lead, mercury and cadmium. Accordingly, considering the observations in our study, the clinical application of MBA combinations could find greater use, because synergism effects give efficacy at much lower working concentrations. Furthermore, combination therapy would help to decrease the evolution of antimicrobial resistance in bacteria [10,40,41] as it is worth noting that resistance to most metal ion species already exists.

One of the biggest problems with controlling and treatment of bacterial infections is strong recovery potency of certain bacteria after exposing with antibiotics [42,43]. Particularly, there is a greater chance of developing antibiotic resistance in antibiotic recovering bacterium compared to unchallenged bacteria [44]. Therefore, in regard to the recovery potency of the three different bacteria surveyed, we found in this study that the synergy mixes led to less recovery compared exposure to the individual metals alone. We also observed the recovery from synergistic application to be different between media types, which is most likely due to the overall physiological fitness differences.

## 4. Materials and Methods

### 4.1. Bacterial Strains and Culture Media

Bacterial strains were stored at −70 °C in Micro-bank vials as described by the manufacturer (proLab Diagnostics, Richmond Hill, ON, Canada). The three ATCC bacterial strains, *P. aeruginosa* ATCC 27853, *S. aureus* ATCC 25923 and *E. coli* ATCC 25922, were used for all experiments. The three different media, Luria broth (LB, VWR chemicals, Lot# 190756384), Mueller–Hinton broth (MHB, BD Bacto, Oxoid, Basingstoke, UK Cat# X296B), and simulated wound fluid (SWF) (50% peptone water (0.85% NaCl, 0.1 g L^−1^ peptone):50% fetal calf serum (GIBCO, Thermo Fisher Scientific, Waltham, MA, USA, Lot# 2212202RP)) were used as the growth medium and for susceptibility testing media in this study [45,46].

### 4.2. Stock and Working Metal(loid)-Based Antibiotic (MBA) Solutions

Nine metal(loid)-based antibiotics (MBAs), including, silver nitrate (AgNO_3_, Sigma-Aldrich, St Louis, MO, USA, Lot#39F-3539), copper (II) sulfate (CuSO_4_, Sigma-Aldrich, St Louis, MO, USA, Cat#C1297-100G), gallium (III) nitrate (Ga(NO_3_)_3_•H_2_O, Sigma-Aldrich, St Louis, MO, USA, Cat#289892-25G) and nickel sulfate (NiSO_4_•6H_2_O, Sigma-Aldrich, St Louis, MO, USA, Lot#68H0027), hydrogen tetrachloroaurate (III) trihydrate (AuCl_4_•3H_2_O, Alfa Aesar Tweksbury, MA, USA, Lot#U17G046), aluminum sulfate (Al_2_(SO_4_)_3_•H_2_O, Norwood, OH, USA, Cat#UCC000TA8), sodium selenite (Na_2_SeO_3_, Alfa Aesar, Ward Hill, MA, USA, Lot#61400984), potassium tellurite (K_2_TeO_3_, Sigma-Aldrich, St Louis, MO, USA, Cat#P0677-25G), and zinc sulfate (ZnSO_4_•7H_2_O, Fisher Scientific Fair Lawn, NJ, USA, Lot#723689). Stock solutions for Ag were made up to 50 mM, and for all other MBA, they were made up to 1 M; working solutions for Ag were made up to 5 mM, and for all other MBA, they were made up to 100 mM in distilled and deionized (dd) H_2_O. Stocks were defined as dissolved from a lack of turbidity in the solution. All stock metal dilutions were stored in glass vials stored at room temperature in a dark place for no longer than 2 weeks. No more than 30 min before experimental use, working solutions were made from stock metal solutions in equal amounts of each media.

Antimicrobial assays were performed in a 96-well plate (the challenge plate), and serial dilutions of each metal with a dilution factor of 2 were prepared; reservation of the first column served as a negative control (media, 0 mM metal salt and no bacteria), and the last column served as a positive control (media and bacteria, 0 mM metal salt and with bacteria).

### 4.3. Minimum Inhibitory Concentration (MIC) Assay

Briefly, −70 °C stored bacteria were sub-cultured two times overnight (O/N) 37 °C on agar plates to obtain pure single colony. A total of 75 µL of the desired concentration of metal salt stock (provided in the media) was added to the 96 wells, and 75 µL of the 150-fold diluted 1.0 McFarland standardized inoculum of bacteria (equivalent 1.0 × 10^6^ CFU/mL) was then added to each well. Following this, the plate was incubated for 24 h at 37 °C in a microplate shaker at 150 rpm [46]. MIC was determined by reading the optical density (OD) at 600 nm (OD600), using a Thermomax microtiter plate reader with Softmax Pro data analysis software (Molecular Devices, Sunnyvale, CA, USA). The last well which had no bacterial growth by OD, was defined as the MIC. In the case of Ga and Al, turbidity was found to be high at concentrations > 4 mM (despite adding them to media after they were completely dissolved in the ddH_2_O as the stock), suggesting insoluble complexes forming with media components. This led to the MIC being more difficult to define; therefore, colony-forming units (CFU) were obtained for the determination of the exact MIC in these two metals.

### 4.4. Minimum Bactericidal Concentration (MBC) Assay and Recovery Potency of Bacteria

At the end of the MIC determination experiment, 10 µL of each MIC well was transferred in a 140 µL of the same fresh media in a new 96 plate and incubated for 24 h at 37 °C in a microplate shaker at 150 rpm. MBC was determined by reading the optical density at 600 nm (OD600) of the recovery plates using a Thermomax microtiter plate reader with Softmax Pro data analysis software (Molecular Devices, Sunnyvale, CA, USA). The last well that had no bacterial growth by OD600 was defined as the MBC. For analysis of recovery potency of the exposed bacteria with metals, plates were read at 2, 4, and 24 h.

### 4.5. Synergism High-Throughput Susceptibility Testing of Microbial Planktonic Growth

“Checkerboard” arrangements of MBA combinations were made in the 96-well microtiter plates as previously described [10,47]. When prepared, each checkerboard microtiter plate had one negative control column (media without bacteria and MBAs) and one column of growth controls as a positive control (without MBA and with media and bacteria,); 10 different concentrations of MBAs alone, 8 different concentrations of Ag alone, and each MBA and Ag in 80 different combinations of concentrations were in each checkboard. For each checkboard analysis, the same MIC and MBC steps indicated above were conducted to survey the bacteriostatic, bactericide, and reading synergism effects of the MBA combinations.

### 4.6. Determination of FIC (Fractional Inhibitory Concentration) for the Detection of Synergism Effects

The synergistic interaction rules suggested by the American Society for Microbiology for the testing of planktonic cells are used for both MIC and MBC synergism data obtained here [47]. The fractional inhibitory concentration (FIC) and fractional bactericidal concentration (FBC) index for each combination of antimicrobial agents were calculated with the following formula:

FIC = MIC antibiotic A in combination/MIC antibiotic A alone + MIC antibiotic B in combination/MIC antibiotic B alone.

FBC = MBC antibiotic A in combination/MBC antibiotic A alone + MBC antibiotic B in combination/MBC antibiotic B alone.

To evaluate antimicrobial interactions, we used the lowest FIC/FBC index method as described by Bonapace et al [48]. The lowest FIC/FBC obtained for all inhibitory or bactericidal combinations on the checkerboard was considered the FIC/FBC for the pair. Finally, FIC/FBC were interpreted as follows: FIC/FBC < 0.8 = synergy, FIC/FBC ≥ 0.8 and ≤ 1.2 = partial synergy, FIC/FBC > 1.2 = antagonistic. 

### 4.7. Synergism Exposure Growth Curve Assays

Synergism time–exposure growth curve assays were performed similarly to those described elsewhere [49,50]. Briefly, combinations that had the highest bactericidal synergism effect against each indicator strains were selected for synergism evaluation for growth in the presence of the defined dosage of MBA formulations. The initial 1.0 × 10^6^ CFU/mL inoculation of *E. coli* ATCC 25922 with combination and/or single dosage of 0.015 mM Ag and 4 mM of Zn was added to MHB. Similarly, *S. aureus* ATCC 25,923 with combination and/or single dosage of 0.125 mM Ag and 1 mM of Zn was added to SWF, and *P. aeruginosa* ATCC 27853 with combination and/or single dosage of 0.015 mM Ag with 0.03 mM Au added to MHB. All incubations were conducted for 24 h at 37 °C in a microplate shaker at 150 rpm. Samples were taken at 0, 2, 4, 8, 16, and 24 h and appropriately diluted; 100 μL of the diluted bacterial solution was uniformly applied to the agar form of each media and incubated at 37 °C for 24 h to obtain viable cell counts (CFU/mL). Synergism effects were interpreted as the following: the bacterial colony number decreased by more than 2 log10 CFU/mL in the combination group considered as synergy. The decrease in bacterial colonies less than 2 log10 CFU/mL was considered to be partial synergy; the increase in bacterial colony number was greater than 2 log10 CFU/mL, which was considered to be antagonistic [49,50].

### 4.8. Statistical Tests and Data Analysis

All data organization, analysis, mean, mode, standard deviation, calculation of FIC, and the three-dimensional graphical representations were performed using Microsoft Excel 365 (Microsoft Corporation, Redmond, WA, USA). All experiments were repeated at least three times, and experiments that had more variable results were repeated seven times.

## 5. Conclusions

Here, we found many metal(loid) combinations that are synergistic with Ag for antimicrobial efficacies. If we consider SWF as the most representative of a wound environment, the best combination for efficacy for control of *P. aeruginosa* and *S. aureus* is silver nitrate with potassium tellurite, and for *E. coli*, it is silver nitrate with tetrachloroaurate. For the killing of bacteria (MBC), the combination of silver nitrate with zinc sulfate against *S. aureus* would be the best option, while that for both *E. coli* and *P. aeruginosa* would be silver nitrate with tetrachloroaurate. Other combinations with other metal ions were also synergistic, and while these were the most effective, other metals may be more cost effective using less “precious metal” in larger-scale applications. Overall, the combination of silver nitrate with potassium tellurite, tetrachloroaurate or zinc sulfate, would be the best bactericide and bacteriostatic synergism for all three bacteria. The data here are now a guideline for different formulations in different materials and coatings.

## Figures and Tables

**Table 1:** Minimum inhibitory concentration (MIC) and minimum bactericidal concentration (MBC) of different metal(loid)-based antibiotics (MBAs) for *Pseudomonas aeruginosa*, *Staphylococcus aureus* and *Escherichia coli*.

	MBAs	*E. coli*			*S. aureus*			*P. aeruginosa*		
		MHB	LB	SWF	MHB	LB	SWF	MHB	LB	SWF
**MIC** **Mode** **(range)** **(mM)**	**Ag**	**0.015** **(0.015–0.65)**	0.125 (0.03–0.125)	0.125 (0.065–0.25)	**0.03** **(0.03–0.125)**	0.125 (0.125–0.25)	0.125 (0.06–0.25)	0.03 (0.015–0.065)	0.125 (0.06–0.125)	0.125 (0.15–0.25)
	**Cu**	8 (4–16)	4 (4–16)	1 (1–4)	8 (4–8)	8 (4–16)	2 (1–4)	8 (4–32)	8 (4–16)	4 (2–4)
	Ga *	8 (4–16)	8 (8–32)	2 (2–4)	8 (4–32)	12.5 (12.5–25)	25 (6.25–25)	0.8 (0.4–1.6)	0.8 (0.4–1.6)	4 (2–4)
	**Zn**	2 (1–4)	2 (1–2)	2 (1–4)	4 (1–4)	2 (1–4)	2 (1–4)	8 (4–16)	8 (4–8)	8 (4–16)
	**Te**	0.25 (0.015–0.25)	**0.015** **(0.015–0.65)**	0.25 (0.125–0.25)	6.25 (1.6–12.5)	0.2 (0.2–1.6)	50<	0.06 (0.06–0.012)	**0.015** **(0.015–0.65)**	0.25 (0.03–0.25)
	**Se**	>50	>50	>50 (25->50)	>50	>50	>50	>50	>50	>50
	**Al**	25 (6.25–25)	6.25 (6.25–25)	12.5 (12.5–25)	>50	12.5 (6.25–25)	25 (6.25–25)	6.25 (6.25–12.5)	12.5 (12.5–25)	6.25 (6.25–25)
	**Ni**	4 (2–8)	2 (1–4)	1 (1–4)	4 (2–8)	2 (1–4)	2 (1–2)	8 (2–16)	8 (2–8)	2 (1–4)
	**Au**	0.25 (0.25–0.5)	0.1 (0.1–0.25)	0.5 (0.25–0.5)	0.1 (0.06–0.125)	0.2 (0.125–0.25)	0.25 (0.25–0.5)	0.25 (0.25–0.5)	0.25 (0.25–0.5)	0.25 (0.25–1)
**MBC** **Mode** **(range)** **(mM)**	**Ag**	**0.125** **(0.015–0.25)**	0.25 (0.125–4)	0.25 (0.065–0.5)	1 (0.25–1)	1 (0.5–1)	1 (0.5–1)	**0.125** **(0.03–0.25)**	0.25 (0.125–0.25)	0.5 (0.5–1)
	**Cu**	8 (4–16)	8 (4–16)	2 (1–4)	16 (4–32)	16 (8–16)	8 (4–32)	8 (4–16)	16 (4–32)	4 (2–4)
	Ga *	12.5 (12.5–25)	25 (12.5–50)	6.25 (6.25–25)	12.5 (12.5–50)	25 (6.25–25)	50 (6.25–50)	3.1 (1.5–12.5)	6.25 (6.25–12.5)	8 (4–16)
	**Zn**	16 (4–32)	2 (2–4)	12.5	8 (2–16)	2 (1–4)	8 (8–16)	8 (2–16)	16	12.5
	**Te**	0.25 (0.25–0.5)	**0.125** **(0.015–0.125)**	0.25 (0.125–0.25)	6.25 (1.6–12.5)	**0.2** **(0.2–6)**	50<	**0.125** **(0.125–0.25)**	0.25 (0.125–0.25)	1 (1–4)
	**Se**	>50	>50	>50	>50	>50	>50	>50	>50	>50
	Al *	25 (12.5–25)	6.25 (6.25–25)	12.5 (12.5–25)	>50	25 (12.5–25)	25 (3–50)	12.5 (12.5–25)	50 (25–100)	25 (12.5–50)
	**Ni**	4 (2–8)	2 (1–4)	1 (1–4)	8 (2–16)	4 (2–8)	8 (2–16)	>32	16 (4–32)	4 (2–8)
	**Au**	0.5 (0.5–8)	0.25 (0.25–2)	0.5 (0.5–2)	0.25 (0.25–1)	0.2 (0.2–1)	0.5 (0.5–4)	0.5 (0.25–0.5)	0.5 (0.25–0.5)	0.5 (0.5–1)

Value ranges are given in parenthesis MHB = Mueller–Hinton broth, LB = Luria–Bertani, SWF = simulated wound fluid. * Turbidity of Ga and Al at concentrations > 4 mM was high, and inhibition of bacteria growth was not always clear; therefore, colony-forming units (CFU) were used for the determination of exact MIC.

**Table 2:** Synergism analysis—ranked list of best synergistic metal(loid) combinations.

		Metal(loid)-Based Antibiotic		Bacteriostatic Synergism (MIC)			Bactericidal Synergism (MBC)		
Media	Bacteria	Agent A	Agent B	FIC	Interpretation	Concentrations (mM)	FBC	Interpretation	Concentrations (mM)
SWF	*S. aureus*	Ag	Te	**0.094>**	Synergy	**0.007 Ag + 3 Te**	0.3	Synergy	0.03 Ag + 6 Te
SWF	*P. aeruginosa*	Ag	Te	**0.3**	Synergy	**0.007 Ag + 0.125 Te**	0.27	Synergy	0.007 Ag + 0.25 Te
SWF	*P. aeruginosa*	Ag	Au	0.36	Synergy	0.03 Ag + 0.125 Au	0.18	Synergy	0.03 Ag + 0.125 Au
LB	*E. coli*	Ag	Au	**0.37**	Synergy	**0.007 Ag + 0.062 Au**	0.49	Synergy	0.031 Ag + 0.031 Au
LB	*E. coli*	Ag	Zn	**0.37**	Synergy	**0.031 Ag + 0.25 Zn**	0.5	Synergy	0.125 Ag + 1 Zn
MHB	*P. aeruginosa*	Ag	Au	0.47	Synergy	0.007 Ag + 0.062 Au	**0.12**	Synergy	**0.015 Ag + 0.03 Au**
LB	*S. aureus*	Ag	Au	1.1	Partial synergy	0.065 Ag + 0.125 Au	**0.138**	Synergy	**0.065 Ag + 0.008 Au**
LB	*S. aureus*	Ag	Au	1.1	Partial synergy	0.065 Ag + 0.125 Au	**0.138**	Synergy	**0.065 Ag + 0.008 Au**
SWF	*S. aureus*	Ag	Zn	0.5	Synergy	0.065 Ag + 0.25 Zn	**0.25**	Synergy	**0.125 Ag + 1 Zn**
MHB	*E. coli*	Ag	Zn	0.9	Partial synergy	0.007 Ag +1 Zn	**0.31**	Synergy	**0.015 Ag + 4 Zn**
LB	*P. aeruginosa*	Ag	Zn	0.37	Synergy	0.031 Ag + 1 Zn	0.5	Synergy	0.125 Ag + 0.25 Zn
SWF	*S. aureus*	Ag	Au	0.47	Synergy	0.007 Ag + 0.125 Au	0.49	Synergy	0.031 Ag + 0.25 Au
SWF	*E. coli*	Ag	Au	0.48	Synergy	0.007Ag + 0.125 Au	0.3	Synergy	0.031 Ag + 0.062 Au
SWF	*E. coli*	Ag	Te	0.48	Synergy	0.031 Ag + 0.031 Te	0.36	Synergy	0.015 Ag + 0.06 Te
MHB	*E. coli*	Ag	Au	0.48	Synergy	0.015 Ag + 0.125 Au	0.49	Synergy	0.031 Ag + 0.031 Au
LB	*P. aeruginosa*	Ag	Au	0.48	Synergy	0.03 Ag + 0.062 Au	0.49	Synergy	0.031 Ag + 0.125 Au
MHB	*S. aureus*	Ag	Cu	0.49	Synergy	0.031 Ag + 2 Cu	0.31	Synergy	0.031 Ag + 4 Cu
MHB	*P. aeruginosa*	Ag	Ni	0.5	Synergy	0.007 Ag + 1 Ni	0.23	Synergy	0.007 Ag + 8 Ni
MHB	*S. aureus*	Ag	Zn	0.5	Synergy	0.031 Ag + 0.125 Zn	0.31	Synergy	0.125 Ag + 0.5 Zn
SWF	*E. coli*	Ag	Zn	0.5	Synergy	0.007 Ag + 3 Zn	1	Partial synergy	0.007 Ag + 12.5 Zn
MHB	*S. aureus*	Ag	Ni	0.51	Synergy	0.015 Ag + 0.125 Ni	1	Partial synergy	0.007 Ag + 8 Ni
SWF	*P. aeruginosa*	Ag	Zn	0.53	Synergy	0.125 Ag + 0.2 Zn	0.49	Synergy	0.125 Ag + 3 Zn
LB	*P. aeruginosa*	Ag	Ni	0.55	Synergy	0.007 Ag + 4 Ni	0.51	Synergy	0.065 Ag + 4 Ni
SWF	*P. aeruginosa*	Ag	Ni	0.55	Synergy	0.007 Ag + 2 Ni	0.62	Synergy	0.25 Ag + 0.5 Ni
LB	*E. coli*	Ag	Cu	0.58	Synergy	0.065 Ag + 0.5 Cu	0.5	Synergy	0.065 Ag + 2 Cu
SWF	*E. coli*	Ag	Ni	0.6	Synergy	0.007 Ag + 1 Ni	1	Partial synergy	0.007 Ag + 1 Ni
MHB	*E. coli*	Ag	Se	0.6	Synergy	0.015 Ag + 12.5 Se	ND	ND	ND
LB	*S. aureus*	Ag	Zn	0.62	Synergy	0.015 Ag + 1 Zn	0.5	Synergy	0.125 Ag + 0.5 Zn
MHB	*E. coli*	Ag	Al	0.62	Synergy	0.015 Ag + 0.1 Al	1.1	Partial synergy	0.125 Ag + 6.25 Al
LB	*E. coli*	Ag	Ni	0.7	Synergy	0.065 Ag + 0.5 Ni	1.05	Partial synergy	0.007 Ag + 2 Ni
MHB	*E. coli*	Ag	Ni	0.7	Synergy	0.065 Ag + 1 Ni	1.05	Partial synergy	0.007 Ag + 4 Ni
MHB	*E. coli*	Ag	Te	0.7	Synergy	0.007 Ag + 0.06 Te	1.06	Partial synergy	0.015 Ag + 0.015 Te
MHB	*S. aureus*	Ag	Te	0.7	Synergy	0.007 Ag + 0.06 Te	1.06	Partial synergy	0.015 Ag + 0.015 Te
MHB	*P. aeruginosa*	Ag	Zn	0.72	Synergy	0.007 Ag + 4 Zn	0.31	Synergy	0.031 Ag + 0.5 Zn
MHB	*P. aeruginosa*	Ag	Te	0.73	Synergy	0.015 Ag + 0.015 Te	0.96	Partial synergy	0.015 Ag + 0.06 Te
SWF	*P. aeruginosa*	Ag	Cu	0.75	Synergy	0.065 Ag + 2 Cu	0.75	Synergy	0.25 Ag + 1 Cu/0.125 Ag + 2 Cu
LB	*S. aureus*	Ag	Ni	0.76	Synergy	0.125 Ag + 1 Ni	1	Partial synergy	0.007 Ag + 4 Ni
SWF	*E. coli*	Ag	Al	0.76	Synergy	0.065 Ag + 6.25 Al	1	Partial synergy	0.007 Ag + 12.5 Al
SWF	*S. aureus*	Ag	Ni	0.97	Partial synergy	0.031 Ag + 1 Ni	0.37	Synergy	0.125 Ag + 1 Ni
SWF	*S. aureus*	Ag	Cu	1	Partial synergy	0.065 Ag + 1 Cu	0.56	Synergy	0.25 Ag + 0.5 Cu
MHB	*E. coli*	Ag	Cu	1	Partial synergy	0.065 Ag + 0.5 Cu	0.6	Synergy	0.125 Ag + 1 Cu
LB	*S. aureus*	Ag	Cu	1	Partial synergy	0.25 Ag + 0.5 Cu	0.65	Synergy	0.25 Ag + 1 Cu
SWF	*E. coli*	Ag	Cu	1	Partial synergy	0.065 Ag + 0.5 Cu	0.76	Synergy	0.065 Ag + 1 Cu
MHB	*S. aureus*	Ag	Al	1	Partial synergy	0.015 Ag + 0.1 Al	1	Partial synergy	0.031 Ag + 0.4 Al
LB	*E. coli*	Ag	Al	1	Partial synergy	0.031 Ag + 0.4 Al	1.1	Partial synergy	0.125 Ag + 25 Al
SWF	*P. aeruginosa*	Ag	Al	1	Partial synergy	0.125 Ag + 0.2 Al	1.25	antagonistic	0.007 Ag + 50 Al
SWF	*S. aureus*	Ag	Al	1	Partial synergy	0.065 Ag + 12.5 Al	1.25	antagonistic	0.007 Ag + 50 Al
LB	*P. aeruginosa*	Ag	Cu	1	Partial synergy	0.065 Ag + 0.5 Cu	O.5	Synergy	0.065 Ag + 4 Cu
MHB	*P. aeruginosa*	Ag	Cu	1	Partial synergy	0.065 Ag + 0.5 Cu	O.5	Synergy	0.065 Ag + 4 Cu
MHB	*S. aureus*	Ag	Au	1.1	Partial synergy	0.015 Ag + 0.062 Au	0.58	Synergy	0.065 Ag + 0.016 Au
LB	*S. aureus*	Ag	Al	1.1	Partial synergy	0.031 Ag + 0.8 Al	ND	ND	ND
LB	*P. aeruginosa*	Ag	Al	1.2	antagonistic	0.015 Ag + 0.2 Al	1.2	0.62	0.015 Ag + 1.5 Al
LB	*S. aureus*	Ag	Te	1.25	Antagonistic	0.065 Ag + 0.004 Te	0.73	Synergy	0.25 Ag + 0.06 Te/0.5 Ag + 0.03 Te
MHB	*P. aeruginosa*	Ag	Al	1.5	antagonistic	0.015 Ag + 0.2 Al	ND	ND	ND
LB	*E. coli*	Ag	Te	1.9	Antagonistic	0.065 Ag + 0.004 Te	0.75	Synergy	0.025 Ag + 0.062 Te/0. 5 Ag + 0.062 Te
LB	*P. aeruginosa*	Ag	Te	1.98	Antagonistic	0.065 Ag + 0.016 Te	0.55	Synergy	0.007 Ag + 0.125 Te/0.065 Ag + 0.008 Te
LB	*E. coli*	Ag	Se	ND	ND	ND	ND	ND	ND
SWF	*E. coli*	Ag	Se	ND	ND	0.065 Ag + 3 Se	ND	ND	0.065 Ag + 12.5 Se
LB	*P. aeruginosa*	Ag	Se	ND	ND	ND	ND	ND	ND
MHB	*P. aeruginosa*	Ag	Se	ND	ND	0.015 Ag + 25 Se	ND	ND	ND
SWF	*P. aeruginosa*	Ag	Se	ND	ND	0.065 Ag + 25 Se	ND	ND	0.125 Ag + 12.5 Se
LB	*S. aureus*	Ag	Se	ND	ND	0.065 Ag + 25 Se	ND	ND	0.125 Ag + 12.5 Se
MHB	*S. aureus*	Ag	Se	ND	ND	ND	ND	ND	ND
SWF	*S. aureus*	Ag	Se	ND	ND	ND	ND	ND	0.125 Ag + 0.8 Se

The fractional inhibitory/biocidal concentration FIC/FBC < 0.8 = synergy, FIC/FBC ≥ 0.8 and ≤ 1.2 = partial synergy, FIC/FBC > 1.2 = antagonistic. MBAs = metal-based antibiotics, MIC = minimum inhibitory concentration, MBC = minimum bactericidal concentration, MHB = Mueller–Hinton broth, LB = Luria–Bertani, SWF = simulated wound fluid. ND= results could not be determined from the concentration ranges examined experimentally, i.e., the agents did not effectively kill the biofilms.

## Supplementary Materials

The following are available online at https://www.mdpi.com/2079-6382/9/12/853/s1, Figure S1. *E. coli* in LB media. A. Minimum inhibitory concentration (MIC) and B. minimum bactericidal concentration (MBC) synergism (syn) effects of silver nitrate (AgNO_3_) combined with copper (II) sulfate (CuSO_4_). Figure S2. A. Minimum inhibitory concentration (MIC) and B. minimum bactericidal concentration (MBC) synergism (syn) effects of silver nitrate (AgNO_3_) combined with copper (II) sulfate (CuSO_4_) against *E. coli* in MHB media. Figure S3. A. Minimum inhibitory concentration (MIC) and B. minimum bactericidal concentration (MBC) synergism (syn) effects of silver nitrate (AgNO_3_) combined with copper (II) sulfate (CuSO_4_) against *E. coli* in simulated wound fluid (SWF) media. Figure S4. A. Minimum inhibitory concentration (MIC) and B. minimum bactericidal concentration (MBC) synergism (syn) effects of silver nitrate (AgNO_3_) combined with copper (II) sulfate (CuSO_4_) against *P. aeruginosa* in LB media. Figure S5. A. Minimum inhibitory concentration (MIC) and B. minimum bactericidal concentration (MBC) synergism (syn) effects of silver nitrate (AgNO_3_) combined with copper (II) sulfate (CuSO_4_) against *P. aeruginosa* in MHB media. Figure S6. A. Minimum inhibitory concentration (MIC) and B. minimum bactericidal concentration (MBC) synergism (syn) effects of silver nitrate (AgNO_3_) combined with copper (II) sulfate (CuSO_4_) against *P. aeruginosa* in SWF media. Figure S7. A. Minimum inhibitory concentration (MIC) and B. minimum bactericidal concentration (MBC) synergism (syn) effects of silver nitrate (AgNO_3_) combined with copper (II) sulfate (CuSO_4_) against *S. aureus* in LB media. Figure S8. A. Minimum inhibitory concentration (MIC) and B. minimum bactericidal concentration (MBC) synergism (syn) effects of silver nitrate (AgNO_3_) combined with copper (II) sulfate (CuSO_4_) against *S. aureus* in MHB media. Figure S9. A. Minimum inhibitory concentration (MIC) and B. minimum bactericidal concentration (MBC) synergism (syn) effects of silver nitrate (AgNO_3_) combined with copper (II) sulfate (CuSO_4_) against *S. aureus* in SWF media. Figure S10. A. Minimum inhibitory concentration (MIC) and B. minimum bactericidal concentration (MBC) synergism (syn) effects of silver nitrate (AgNO_3_) combined with zinc sulfate (ZnSO_4_) against *E. coli* in LB media. Figure S11. A. Minimum inhibitory concentration (MIC) and B. minimum bactericidal concentration (MBC) synergism (syn) effects of silver nitrate (AgNO_3_) combined with zinc sulfate (ZnSO_4_) against *E. coli* in MHB media. Figure S12. A. Minimum inhibitory concentration (MIC) and B. minimum bactericidal concentration (MBC) synergism (syn) effects of silver nitrate (AgNO_3_) combined with zinc sulfate (ZnSO_4_) against *E. coli* in simulated wound fluid (SWF) media. Figure S13. A. Minimum inhibitory concentration (MIC) and B. minimum bactericidal concentration (MBC) synergism (syn) effects of silver nitrate (AgNO_3_) combined with zinc sulfate (ZnSO_4_) against *P. aeruginosa* in LB media. Figure S14. A. Minimum inhibitory concentration (MIC) and B. minimum bactericidal concentration (MBC) synergism (syn) effects of silver nitrate (AgNO_3_) combined with zinc sulfate (ZnSO_4_) against *P. aeruginosa* in MHB media. Figure S15. A. Minimum inhibitory concentration (MIC) and B. minimum bactericidal concentration (MBC) synergism (syn) effects of silver nitrate (AgNO_3_) combined with zinc sulfate (ZnSO_4_) against *P. aeruginosa* in SWF media. Figure S16. A. Minimum inhibitory concentration (MIC) and B. minimum bactericidal concentration (MBC) synergism (syn) effects of silver nitrate (AgNO_3_) combined with zinc sulfate (ZnSO_4_) against *S. aureus* in LB media. Figure S17. A. Minimum inhibitory concentration (MIC) and B. minimum bactericidal concentration (MBC) synergism (syn) effects of silver nitrate (AgNO_3_) combined with zinc sulfate (ZnSO_4_) against *S. aureus* in MHB media. Figure S18. A. Minimum inhibitory concentration (MIC) and B. minimum bactericidal concentration (MBC) synergism (syn) effects of silver nitrate (AgNO_3_) combined with zinc sulfate (ZnSO_4_•) against *S. aureus* in SWF media. Figure S19. A. Minimum inhibitory concentration (MIC) and B. minimum bactericidal concentration (MBC) synergism (syn) effects of silver nitrate (AgNO_3_) combined with potassium tellurite (K_2_TeO_3_) against *E. coli* in LB media. Figure S20. A. Minimum inhibitory concentration (MIC) and B. minimum bactericidal concentration (MBC) synergism (syn) effects of silver nitrate (AgNO_3_) combined with potassium tellurite (K_2_TeO_3_) against *E. coli* in MHB media. Figure S21. A. Minimum inhibitory concentration (MIC) and B. minimum bactericidal concentration (MBC) synergism (syn) effects of silver nitrate (AgNO_3_) combined with tellurite (K_2_TeO_3_) against *E. coli* in simulated wound fluid (SWF) media. Figure S22. A. Minimum inhibitory concentration (MIC) and B. minimum bactericidal concentration (MBC) synergism (syn) effects of silver nitrate (AgNO_3_) combined with tellurite (K_2_TeO_3_) against *P. aeruginosa* in LB media. Figure S23. A. Minimum inhibitory concentration (MIC) and B. minimum bactericidal concentration (MBC) synergism (syn) effects of silver nitrate (AgNO_3_) combined with tellurite (K_2_TeO_3_) against *P. aeruginosa* in MHB media. Figure S24. A. Minimum inhibitory concentration (MIC) and B. minimum bactericidal concentration (MBC) synergism (syn) effects of silver nitrate (AgNO_3_) combined with tellurite (K_2_TeO_3_) against *P. aeruginosa* in SWF media. Figure S25. A. Minimum inhibitory concentration (MIC) and B. minimum bactericidal concentration (MBC) synergism (syn) effects of silver nitrate (AgNO_3_) combined with tellurite (K_2_TeO_3_) against *S. aureus* in LB media. Figure S26. A. Minimum inhibitory concentration (MIC) and B. minimum bactericidal concentration (MBC) synergism (syn) effects of silver nitrate (AgNO_3_) combined with tellurite (K_2_TeO_3_) against *S. aureus* in MHB media. Figure S27. A. Minimum inhibitory concentration (MIC) and B. minimum bactericidal concentration (MBC) synergism (syn) effects of silver nitrate (AgNO_3_) combined with tetrachloroaurate (iii) (HA, AuCl_4_) against *E. coli* in LB media. Figure S28. A. Minimum inhibitory concentration (MIC) and B. minimum bactericidal concentration (MBC) synergism (syn) effects of silver nitrate (AgNO_3_) combined with tetrachloroaurate (iii) (HA, AuCl_4_) against *E. coli* in MHB media. Figure S29. A. Minimum inhibitory concentration (MIC) and B. minimum bactericidal concentration (MBC) synergism (syn) effects of silver nitrate (AgNO_3_) combined with tetrachloroaurate (iii) (HA, AuCl_4_) against *E. coli* in simulated wound fluid (SWF) media. Figure S30. A. Minimum inhibitory concentration (MIC) and B. minimum bactericidal concentration (MBC) synergism (syn) effects of silver nitrate (AgNO_3_) combined with tetrachloroaurate (iii) (HA, AuCl_4_) against *P. aeruginosa* in LB media. Figure S31. A. Minimum inhibitory concentration (MIC) and B. minimum bactericidal concentration (MBC) synergism (syn) effects of silver nitrate (AgNO_3_) combined with tetrachloroaurate (iii) (HA, AuCl_4_) against *P. aeruginosa* in SWF media. Figure S32. A. Minimum inhibitory concentration (MIC) and B. minimum bactericidal concentration (MBC) synergism (syn) effects of silver nitrate (AgNO_3_) combined with tetrachloroaurate (iii) (HA, AuCl_4_) against *S. aureus* in LB media. Figure S33. A. Minimum inhibitory concentration (MIC) and B. minimum bactericidal concentration (MBC) synergism (syn) effects of silver nitrate (AgNO_3_) combined with tetrachloroaurate (iii) (HA, AuCl_4_) against *S. aureus* in MHB media. Figure S34. A. Minimum inhibitory concentration (MIC) and B. minimum bactericidal concentration (MBC) synergism (syn) effects of silver nitrate (AgNO_3_) combined with tetrachloroaurate (iii) (HA, AuCl_4_) against *S. aureus* in SWF media. Figure S35. A. Minimum inhibitory concentration (MIC) and B. minimum bactericidal concentration (MBC) synergism (syn) effects of silver nitrate (AgNO_3_) combined with nickel sulfate (NiSO_4_) against *E. coli* in LB media. Figure S36. A. Minimum inhibitory concentration (MIC) and B. minimum bactericidal concentration (MBC) synergism (syn) effects of silver nitrate (AgNO_3_) combined with nickel sulfate (NiSO_4_) against *E. coli* in MHB media. Figure S37. A. Minimum inhibitory concentration (MIC) and B. minimum bactericidal concentration (MBC) synergism (syn) effects of silver nitrate (AgNO_3_) combined with nickel sulfate (NiSO_4_) against *E. coli* in simulated wound fluid (SWF) media. Figure S38. A. Minimum inhibitory concentration (MIC) and B. minimum bactericidal concentration (MBC) synergism (syn) effects of silver nitrate (AgNO_3_) combined with nickel sulfate (NiSO_4_) against *P. aeruginosa* in LB media. Figure S39. A. Minimum inhibitory concentration (MIC) and B. minimum bactericidal concentration (MBC) synergism (syn) effects of silver nitrate (AgNO_3_) combined with nickel sulfate (NiSO_4_) against *P. aeruginosa* in MHB media. Figure S40. A. Minimum inhibitory concentration (MIC) and B. minimum bactericidal concentration (MBC) synergism (syn) effects of silver nitrate (AgNO_3_) combined with nickel sulfate (NiSO_4_) against *P. aeruginosa* in SWF media. Figure S41. A. Minimum inhibitory concentration (MIC) and B. minimum bactericidal concentration (MBC) synergism (syn) effects of silver nitrate (AgNO_3_) combined with nickel sulfate (NiSO_4_) against *S. aureus* in LB media. Figure S42. A. Minimum inhibitory concentration (MIC) and B. minimum bactericidal concentration (MBC) synergism (syn) effects of silver nitrate (AgNO_3_) combined with nickel sulfate (NiSO_4_) against *S. aureus* in MHB media. Figure S43. A. Minimum inhibitory concentration (MIC) and B. minimum bactericidal concentration (MBC) synergism (syn) effects of silver nitrate (AgNO_3_) combined with nickel sulfate (NiSO_4_) against *S. aureus* in SWF media. Figure S44. A. Minimum inhibitory concentration (MIC) and B. minimum bactericidal concentration (MBC) synergism (syn) effects of silver nitrate (AgNO_3_) combined with sodium selenite (Na_2_SeO_3_) against *E. coli* in LB media. Figure S45. A. Minimum inhibitory concentration (MIC) and B. minimum bactericidal concentration (MBC) synergism (syn) effects of silver nitrate (AgNO_3_) combined with sodium selenite (Na_2_SeO_3_) against *E. coli* in MHB media. Figure S46. A. Minimum inhibitory concentration (MIC) and B. minimum bactericidal concentration (MBC) synergism (syn) effects of silver nitrate (AgNO_3_) combined with sodium selenite (Na_2_SeO_3_) against *E. coli* in simulated wound fluid (SWF) media. Figure S47. A. Minimum inhibitory concentration (MIC) and B. minimum bactericidal concentration (MBC) synergism (syn) effects of silver nitrate (AgNO_3_) combined with sodium selenite (Na_2_SeO_3_) against *P. aeruginosa* in LB media. Figure S48. A. Minimum inhibitory concentration (MIC) and B. minimum bactericidal concentration (MBC) synergism (syn) effects of silver nitrate (AgNO_3_) combined with sodium selenite (Na_2_SeO_3_) against *P. aeruginosa* in MHB media. Figure S49. A. Minimum inhibitory concentration (MIC) and B. minimum bactericidal concentration (MBC) synergism (syn) effects of silver nitrate (AgNO_3_) combined with sodium selenite (Na_2_SeO_3_) against *P. aeruginosa* in SWF media. Figure S50. A. Minimum inhibitory concentration (MIC) and B. minimum bactericidal concentration (MBC) synergism (syn) effects of silver nitrate (AgNO_3_) combined with sodium selenite (Na_2_SeO_3_) against *S. aureus* in LB media. Figure S51. A. Minimum inhibitory concentration (MIC) and B. minimum bactericidal concentration (MBC) synergism (syn) effects of silver nitrate (AgNO_3_) combined with sodium selenite (Na_2_SeO_3_) against *S. aureus* in MHB media. Figure S52. A. Minimum inhibitory concentration (MIC) and B. minimum bactericidal concentration (MBC) synergism (syn) effects of silver nitrate (AgNO_3_) combined with sodium selenite (Na_2_SeO_3_) against *S. aureus* in SWF media. Figure S53. Recovery potency of *E. coli* ATCC 259 against synergism effects of silver nitrate (AgNO_3_) combined with copper (II) sulfate (CuSO_4_) after 2 h (A), 4 h (B), and 24 h (C); *P. aeruginosa* after 2 h (D), 4 h (E), and 24 h (F); and *S. aureus* after 2 h (G), 4 h (H), and 24 h (I) in LB. Figure S54. Recovery potency of *E. coli* ATCC 259 against synergism effects of silver nitrate (AgNO_3_) combined with copper (II) sulfate (CuSO_4_) after 2 h (A), 4 h (B), and 24 h (C); *P. aeruginosa* after 2 h (D), 4 h (E), and 24 h (F); and *S. aureus* after 2 h (G), 4 h (H), and 24 h (I) in Mueller–Hinton broth (MHB). Figure S55. Recovery potency of *E. coli* ATCC 259 against synergism effects of silver nitrate (AgNO_3_) combined with copper (II) sulfate (CuSO_4_) after 2 h (A), 4 h (B), and 24 h (C); *P. aeruginosa* after 2 h (D), 4 h (E), and 24 h (F); and *S. aureus* after 2 h (G), 4 h (H), and 24 h (I) in simulated wound fluid (SWF). Figure S56. Recovery potency of *E. coli* ATCC 259 against synergism effects of silver nitrate (AgNO_3_) combined with zinc sulfate (ZnSO_4_) after 2 h (A), 4 h (B), and 24 h (C); *P. aeruginosa* after 2 h (D), 4 h (E), and 24 h (F); and *S. aureus* after 2 h (G), 4 h (H), and 24 h (I) in LB. Figure S57. Recovery potency of *E. coli* ATCC 259 against synergism effects of silver nitrate (AgNO_3_) combined with zinc sulfate (ZnSO_4_) after 2 h (A), 4 h (B), and 24 h (C); *P. aeruginosa* after 2 h (D), 4 h (E), and 24 h (F); and *S. aureus* after 2 h (G), 4 h (H), and 24 h (I) in MHB. Figure S58. Recovery potency of *E. coli* ATCC 259 against synergism effects of silver nitrate (AgNO_3_) combined with zinc sulfate (ZnSO_4_) after 2 h (A), 4 h (B), and 24 h (C); *P. aeruginosa* after 2 h (D), 4 h (E), and 24 h (F); and *S. aureus* after 2 h (G), 4 h (H), and 24 h (I) in SWF. Figure S59. Recovery potency of *E. coli* ATCC 259 against synergism effects of silver nitrate (AgNO_3_) combined with tellurite (K_2_TeO_3_) after 2 h (A), 4 h (B), and 24 h (C); *P. aeruginosa* after 2 h (D), 4 h (E), and 24 h (F); and *S. aureus* after 2 h (G), 4 h (H), and 24 h (I) in LB. Figure S60. Recovery potency of *E. coli* ATCC 259 against synergism effects of silver nitrate (AgNO_3_) combined with tellurite (K_2_TeO_3_) after 2 h (A), 4 h (B), and 24 h (C); *P. aeruginosa* after 2 h (D), 4 h (E), and 24 h (F); and *S. aureus* after 2 h (G), 4 h (H), and 24 h (I) in MHB. Figure S61. Recovery potency of *E. coli* ATCC 259 against synergism effects of silver nitrate (AgNO_3_) combined with tellurite (K_2_TeO_3_) after 2 h (A), 4 h (B), and 24 h (C); *P. aeruginosa* after 2 h (D), 4 h (E), and 24 h (F); and *S. aureus* after 2 h (G), 4 h (H), and 24 h (I) in SWF. Figure S62. Recovery potency of *E. coli* ATCC 259 against synergism effects of silver nitrate (AgNO_3_) combined with nickel sulfate (NiSO_4_) after 2 h (A), 4 h (B), and 24 h (C); *P. aeruginosa* after 2 h (D), 4 h (E), and 24 h (F); and *S. aureus* after 2 h (G), 4 h (H), and 24 h (I) in LB. Figure S63. Recovery potency of *E. coli* ATCC 259 against synergism effects of silver nitrate (AgNO_3_) combined with nickel sulfate (NiSO_4_) after 2 h (A), 4 h (B), and 24 h (C); *P. aeruginosa* after 2 h (D), 4 h (E), and 24 h (F); and *S. aureus* after 2 h (G), 4 h (H), and 24 h (I) in MHB. Figure S64. Recovery potency of *E. coli* ATCC 259 against synergism effects of silver nitrate (AgNO_3_) combined with sodium selenite (Na_2_SeO_3_) after 2 h (A), 4 h (B), and 24 h (C); *P. aeruginosa* after 2 h (D), 4 h (E), and 24 h (F); and *S. aureus* after 2 h (G), 4 h (H), and 24 h (I) in SWF. Figure S65. Recovery potency of *E. coli* ATCC 259 against synergism effects of silver nitrate (AgNO_3_) combined with sodium selenite (Na_2_SeO_3_) after 2 h (A), 4 h (B), and 24 h (C); *P. aeruginosa* after 2 h (D), 4 h (E), and 24 h (F); and *S. aureus* after 2 h (G), 4 h (H), and 24 h (I) in LB. Figure S66. Recovery potency of *E. coli* ATCC 259 against synergism effects of silver nitrate (AgNO_3_) combined with sodium selenite (Na_2_SeO_3_) after 2 h (A), 4 h (B), and 24 h (C); *P. aeruginosa* after 2 h (D), 4 h (E), and 24 h (F); and *S. aureus* after 2 h (G), 4 h (H), and 24 h (I) in MHB. Figure S67. Recovery potency of *E. coli* ATCC 259 against synergism effects of silver nitrate (AgNO_3_) combined with sodium selenite (Na_2_SeO_3_) after 2 h (A), 4 h (B), and 24 h (C); *P. aeruginosa* after 2 h (D), 4 h (E), and 24 h (F); and *S. aureus* after 2 h (G), 4 h (H), and 24 h (I) in SWF. Figure S68. Recovery potency of *E. coli* ATCC 259 against synergism effects of silver nitrate (AgNO_3_) combined with tetrachloroaurate (iii) (HA, AuCl_4_) after 2 h (A), 4 h (B), and 24 h (C); *P. aeruginosa* after 2 h (D), 4 h (E), and 24 h (F); and *S. aureus* after 2 h (G), 4 h (H), and 24 h (I) in LB. Figure S69. Recovery potency of *E. coli* ATCC 259 against synergism effects of silver nitrate (AgNO_3_) combined with tetrachloroaurate (iii) (HA, AuCl_4_) after 2 h (A), 4 h (B), and 24 h (C); *P. aeruginosa* after 2 h (D), 4 h (E), and 24 h (F); and *S. aureus* after 2 h (G), 4 h (H), and 24 h (I) in SWF.
