# Supplementary material for: Silver Antibacterial Synergism Activities with Eight Other Metal(loid)-Based Antimicrobials against Escherichia coli, Pseudomonas aeruginosa, and Staphylococcus aureus
Source: Antibiotics (Basel). 2020 Nov 28;9(12):853. doi: 10.3390/antibiotics9120853 (PMC7760997; doi:10.3390/antibiotics9120853)
Supplement: Supplementary file 1 [file antibiotics-09-00853-s001.pdf]

antibiotics

MDPI

Article

Silver Antibacterial Synergism Activities with Eight Other Metal(loid)-Based Antimicrobials against *Escherichia coli*, *Pseudomonas aeruginosa*, and *Staphylococcus aureus*

Ali Pormohammad and Raymond J. Turner *

Department of Biological Sciences, Faculty of Science, University of Calgary, Calgary, Alberta T2N 1N4, Canada; ali.pormohammad@ucalgary.ca

* Correspondence: turnerr@ucalgary.ca; Tel.: +1-403-220-4308

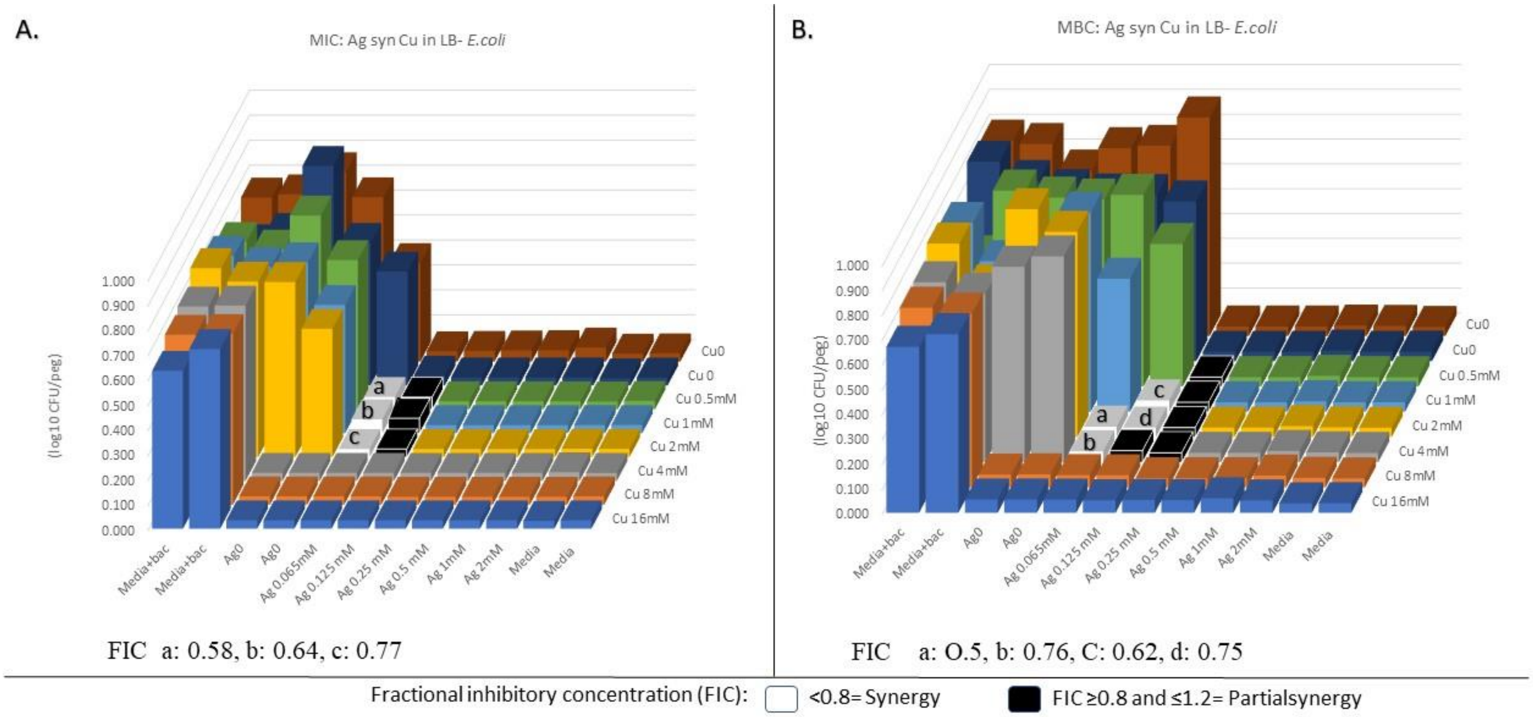

Figure S1. *E. coli* in LB media. A. Minimum inhibitory concentration (MIC) and B. minimum bactericidal concentration (MBC) synergism (syn) effects of silver nitrate ($AgNO_3$) combined with copper (II) sulfate ($CuSO_4$).

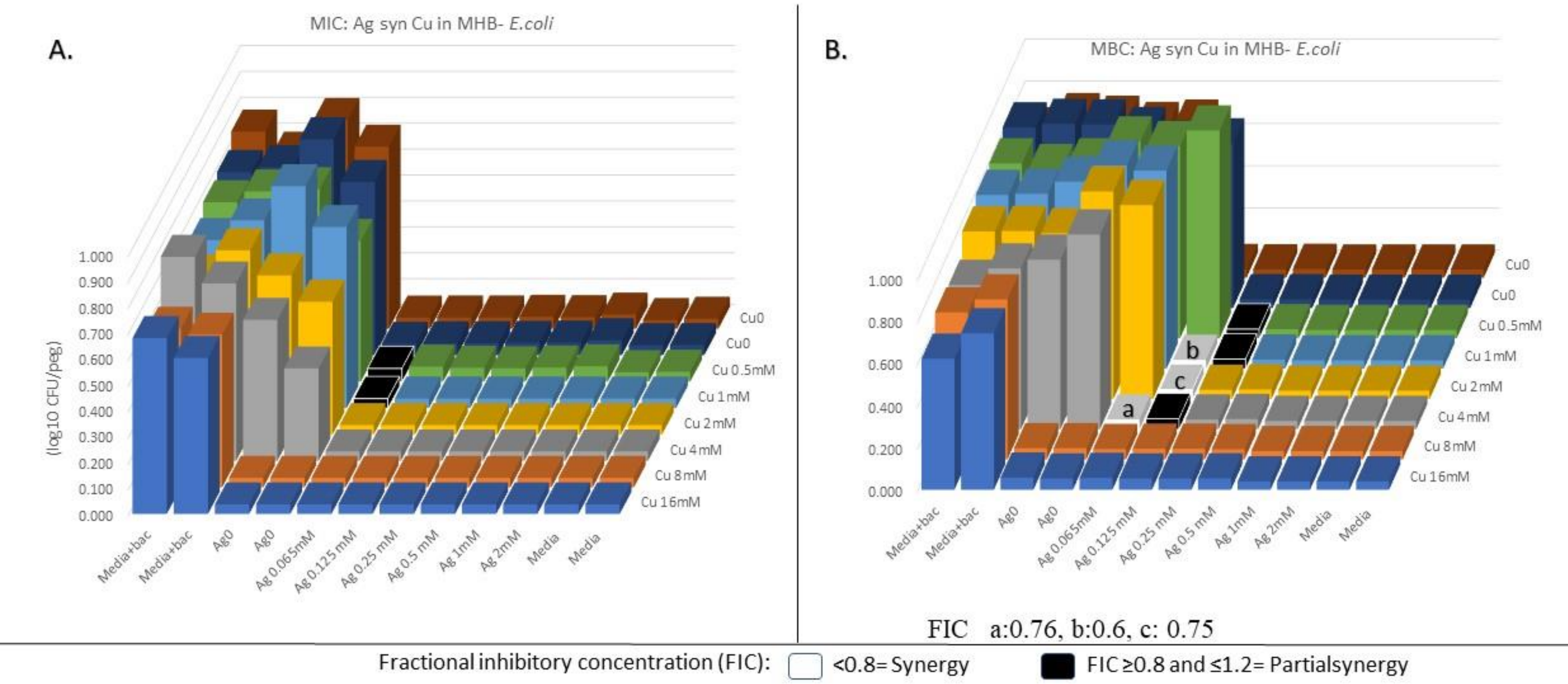

Figure S2. A. Minimum inhibitory concentration (MIC) and B. minimum bactericidal concentration (MBC) synergism (syn) effects of silver nitrate ($AgNO_3$) combined with copper (II) sulfate ($CuSO_4$) against *E. coli* in MHB media.

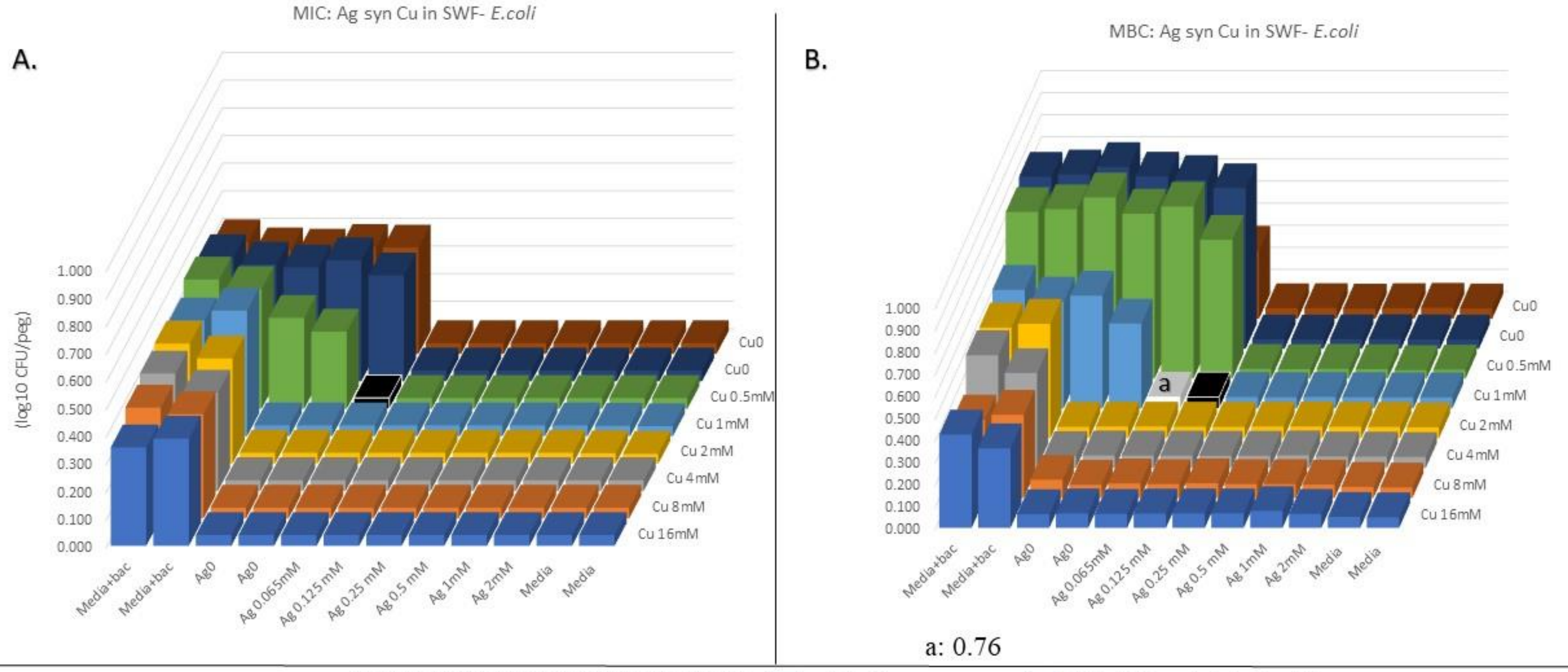

Fractional inhibitory concentration (FIC): <0.8= Synergy FIC ≥0.8 and ≤1.2= Partialsynergy

Figure S3. A. Minimum inhibitory concentration (MIC) and B. minimum bactericidal concentration (MBC) synergism (syn) effects of silver nitrate ($AgNO_3$) combined with copper (II) sulfate ($CuSO_4$) against *E. coli* in simulated wound fluid (SWF) media.

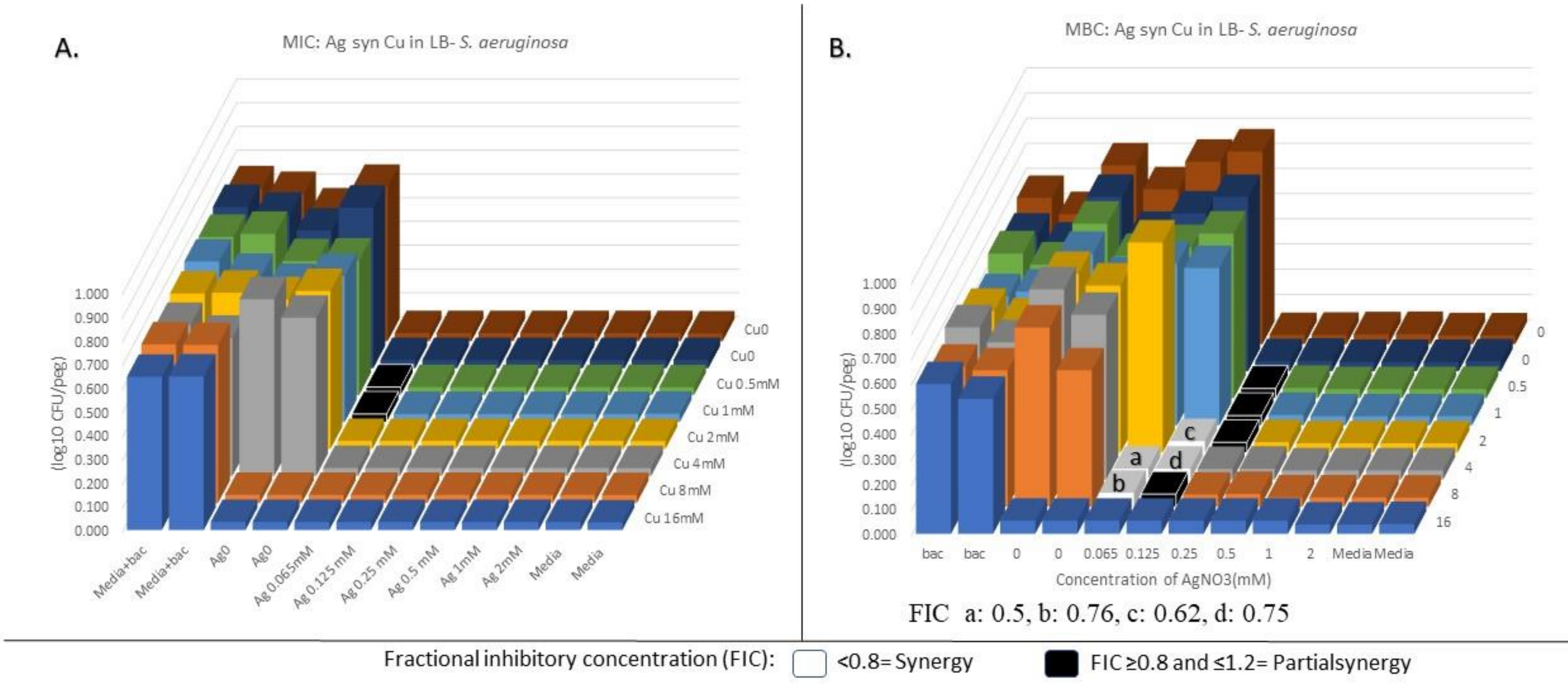

Figure S4. A. Minimum inhibitory concentration (MIC) and B. minimum bactericidal concentration (MBC) synergism (syn) effects of silver nitrate ($AgNO_3$) combined with copper (II) sulfate ($CuSO_4$) against *P. aeruginosa* in LB media

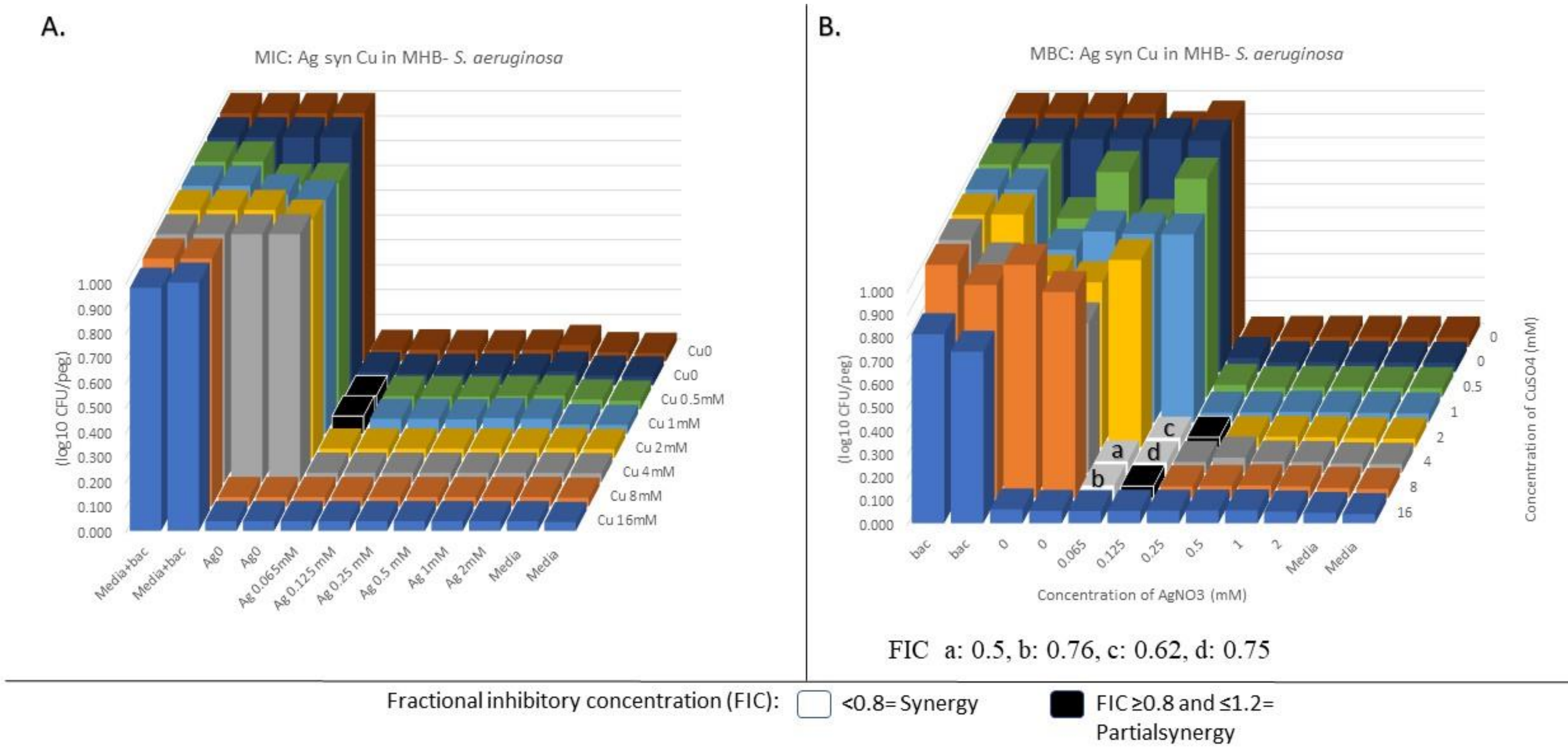

Figure S5. A. Minimum inhibitory concentration (MIC) and B. minimum bactericidal concentration (MBC) synergism (syn) effects of silver nitrate ($AgNO_3$) combined with copper (II) sulfate ($CuSO_4$) against *P. aeruginosa* in MHB media.

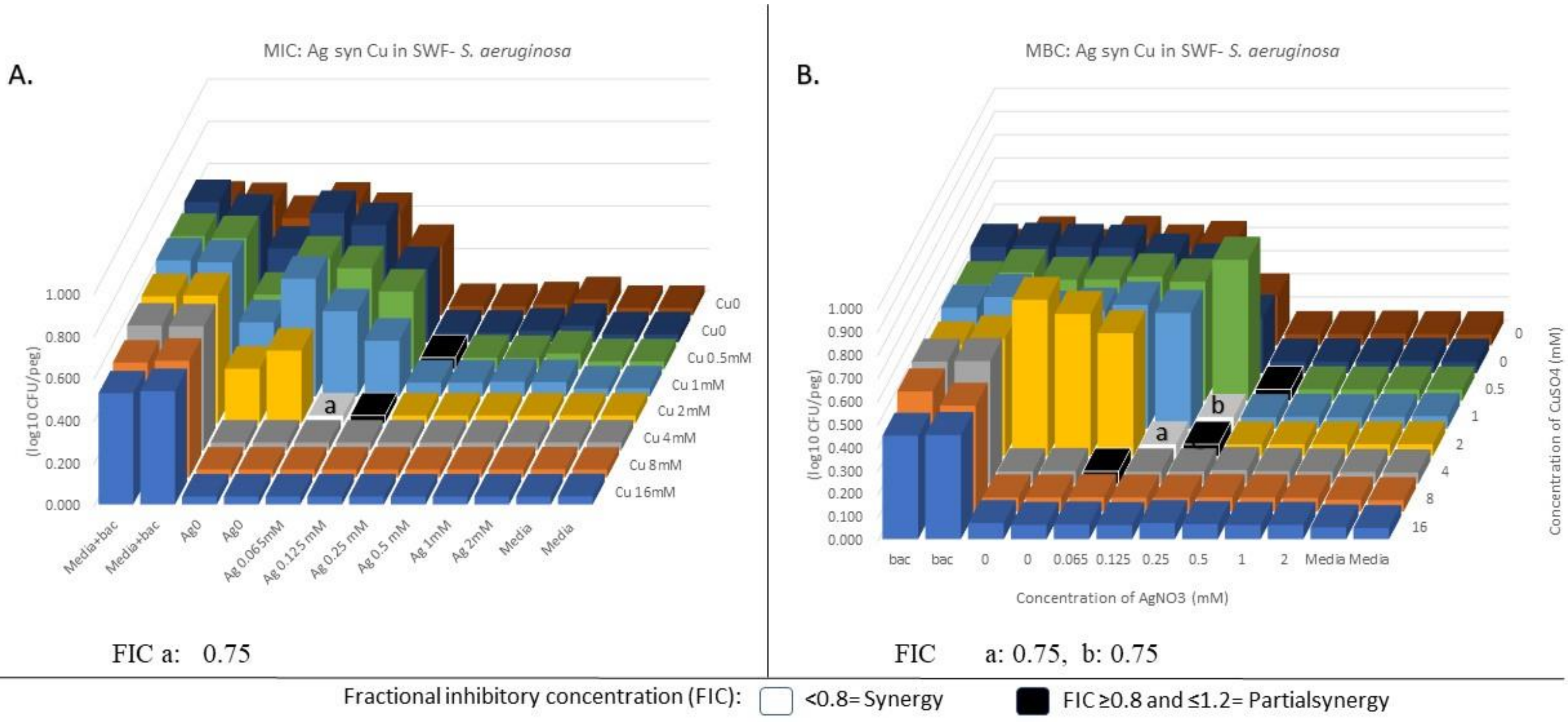

Figure S6. A. Minimum inhibitory concentration (MIC) and B. minimum bactericidal concentration (MBC) synergism (syn) effects of silver nitrate ($AgNO_3$) combined with copper (II) sulfate ($CuSO_4$) against *P. aeruginosa* in SWF media.

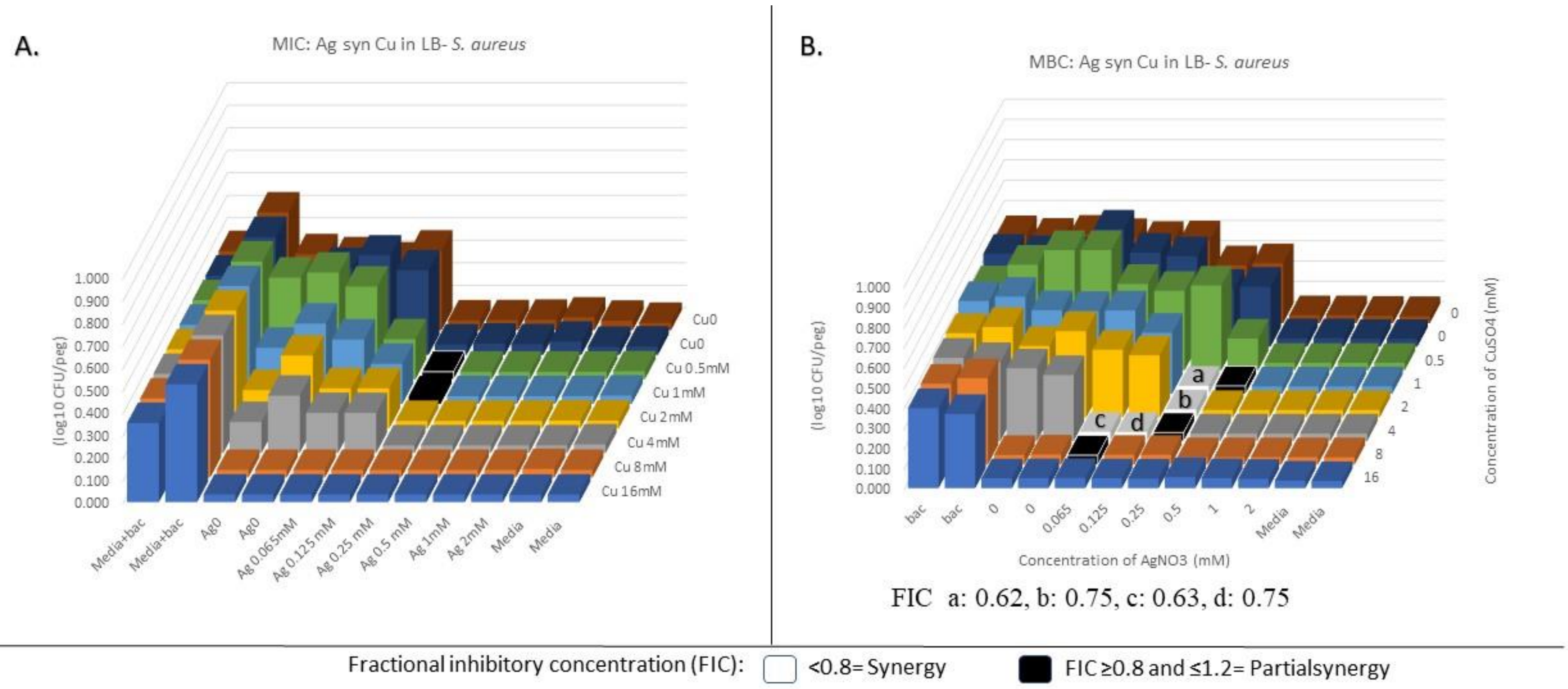

Figure S7. A. Minimum inhibitory concentration (MIC) and B. minimum bactericidal concentration (MBC) synergism (syn) effects of silver nitrate ($AgNO_3$) combined with copper (II) sulfate ($CuSO_4$) against *S. aureus* in LB media.

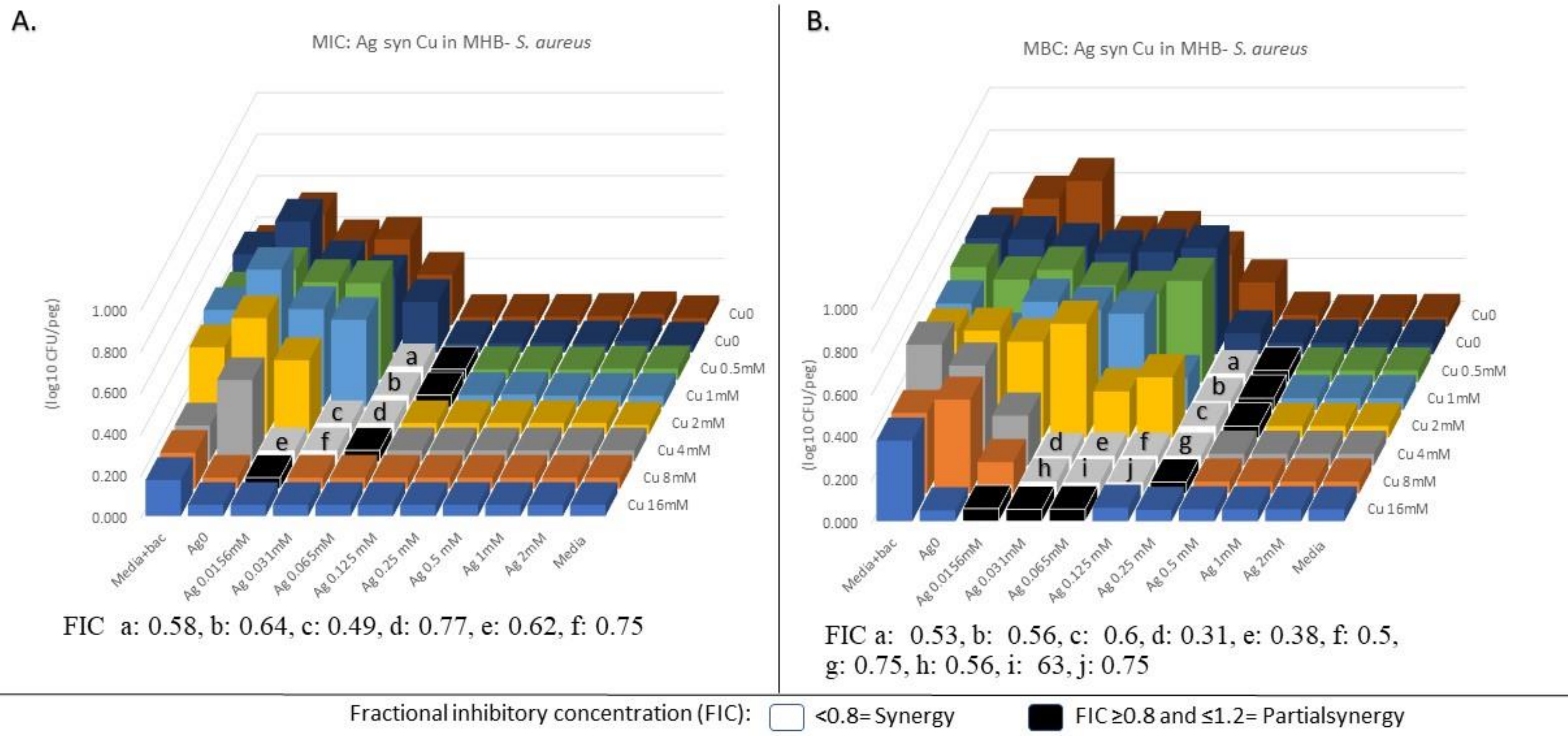

Figure S8. A. Minimum inhibitory concentration (MIC) and B. minimum bactericidal concentration (MBC) synergism (syn) effects of silver nitrate ($AgNO_3$) combined with copper (II) sulfate ($CuSO_4$) against *S. aureus* in MHB media.

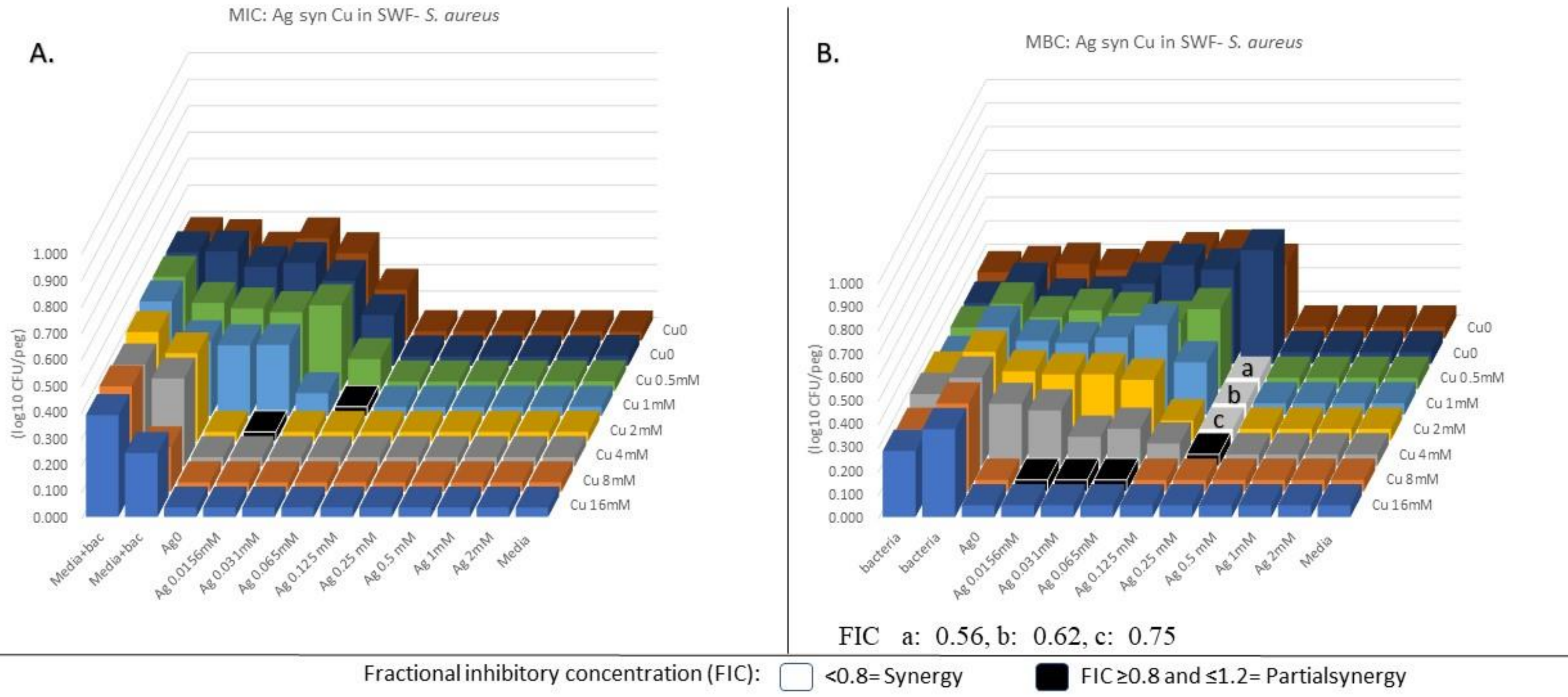

Figure S9. A. Minimum inhibitory concentration (MIC) and B. minimum bactericidal concentration (MBC) synergism (syn) effects of silver nitrate ($AgNO_3$) combined with copper (II) sulfate ($CuSO_4$) against *S. aureus* in SWF media.

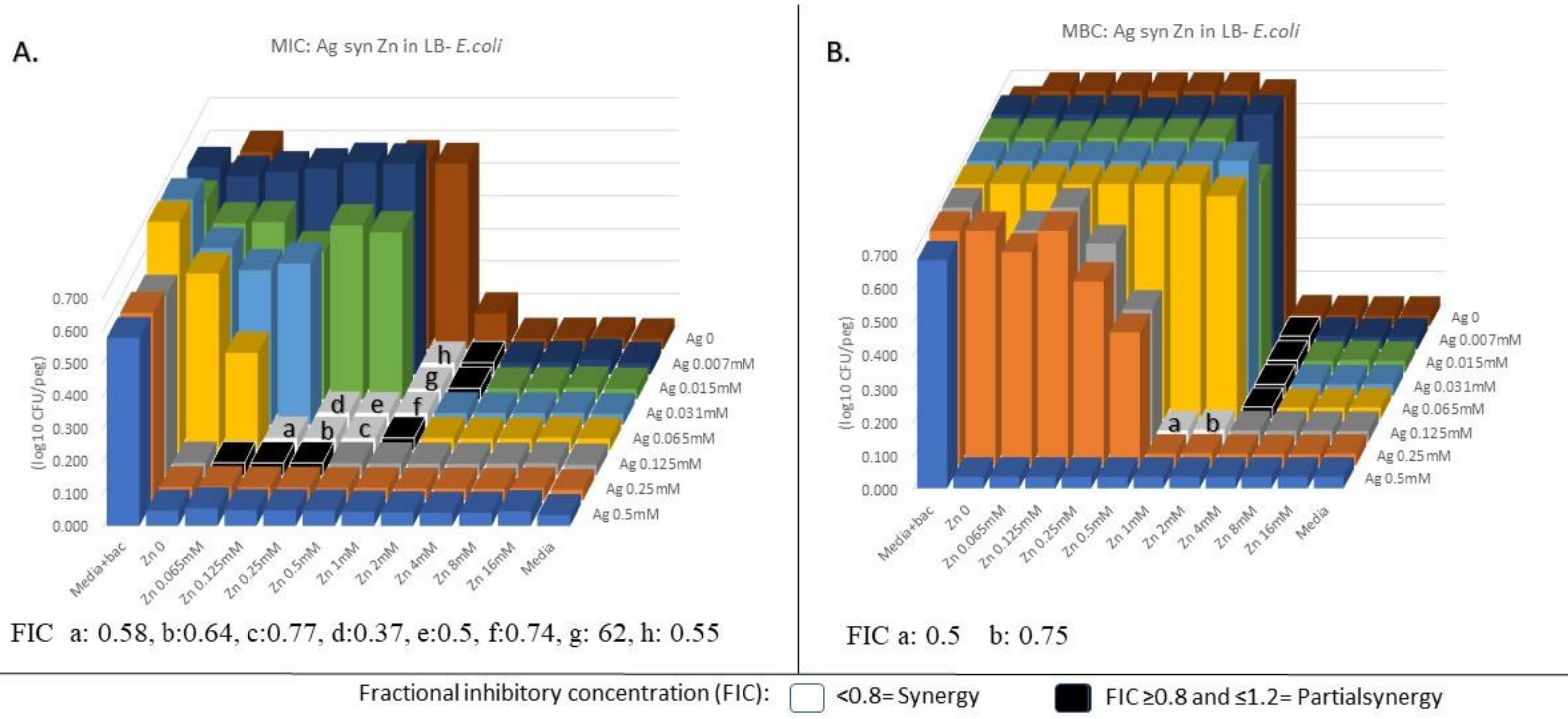

Figure S10. A. Minimum inhibitory concentration (MIC) and B. minimum bactericidal concentration (MBC) synergism (syn) effects of silver nitrate ($AgNO_3$) combined with zinc sulfate ($ZnSO_4$) against *E. coli* in LB media.

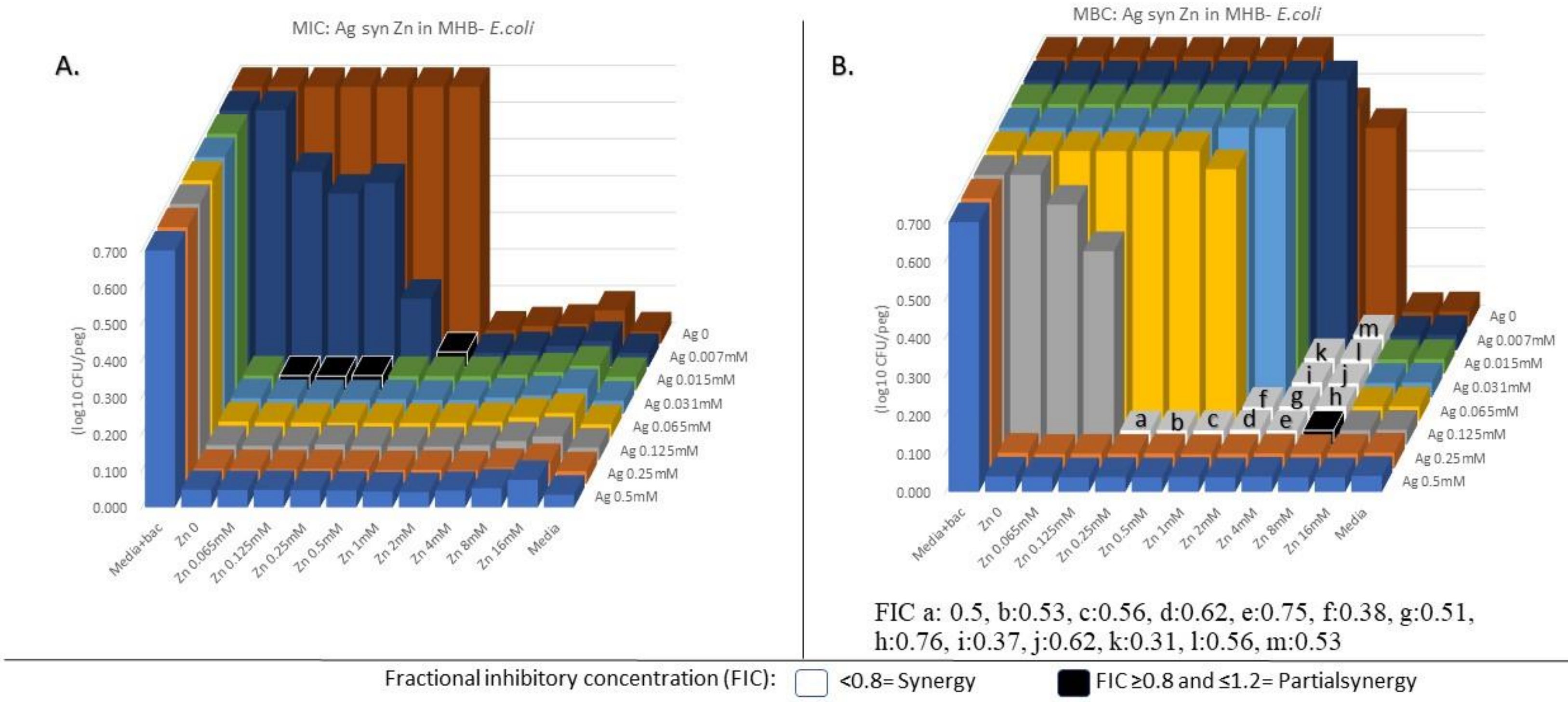

Figure S11. A. Minimum inhibitory concentration (MIC) and B. minimum bactericidal concentration (MBC) synergism (syn) effects of silver nitrate ($AgNO_3$) combined with zinc sulfate ($ZnSO_4$) against *E. coli* in MHB media.

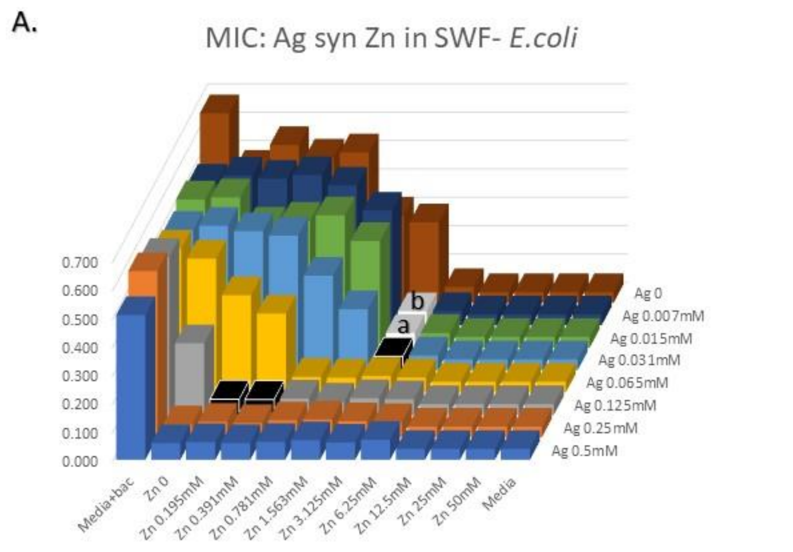

FIC a: 0.51, b:0.54

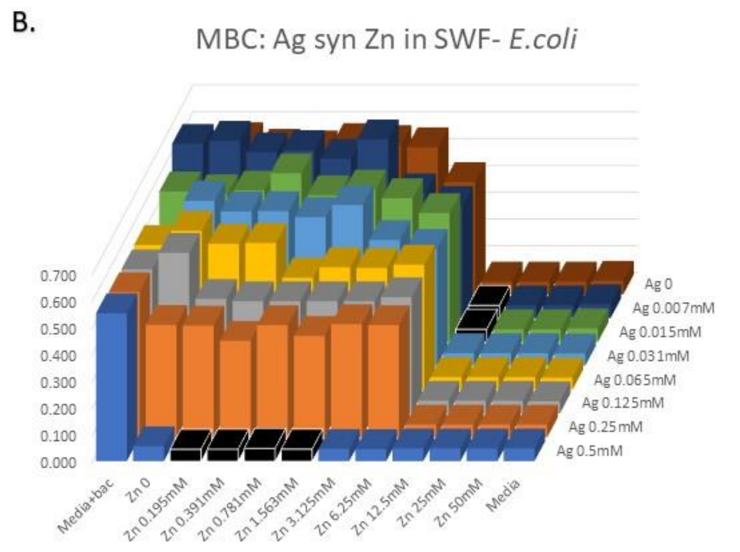

FIC a: 1

Fractional inhibitory concentration (FIC): ☐ <0.8= Synergy ■ FIC ≥0.8 and ≤1.2= Partialsynergy

Figure S12. A. Minimum inhibitory concentration (MIC) and B. minimum bactericidal concentration (MBC) synergism (syn) effects of silver nitrate ($AgNO_3$) combined with zinc sulfate ($ZnSO_4$) against *E. coli* in simulated wound fluid (SWF) media.

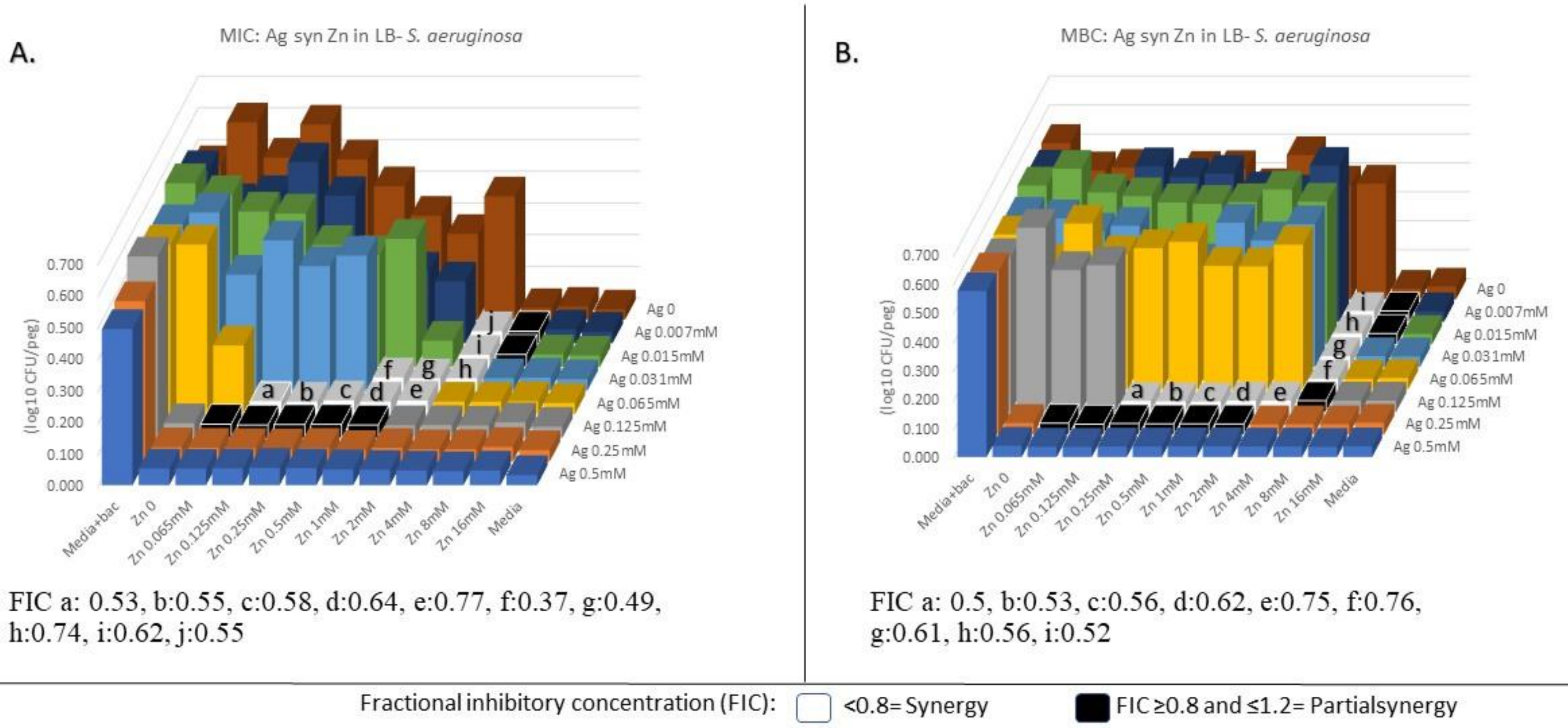

Figure S13. A. Minimum inhibitory concentration (MIC) and B. minimum bactericidal concentration (MBC) synergism (syn) effects of silver nitrate ($AgNO_3$) combined with zinc sulfate ($ZnSO_4$) against *P. aeruginosa* in LB media.

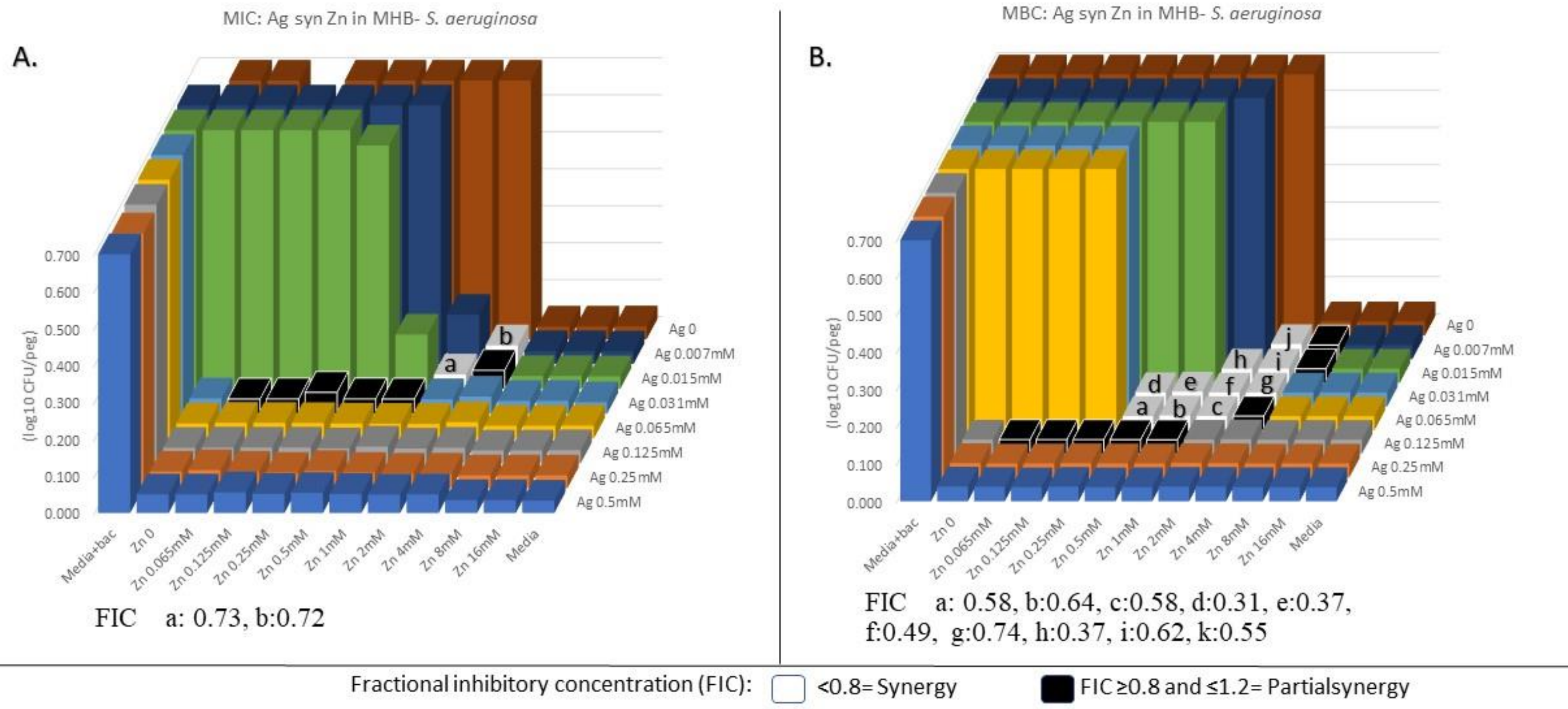

Figure S14. A. Minimum inhibitory concentration (MIC) and B. minimum bactericidal concentration (MBC) synergism (syn) effects of silver nitrate ($AgNO_3$) combined with zinc sulfate ($ZnSO_4$) against *P. aeruginosa* in MHB media.

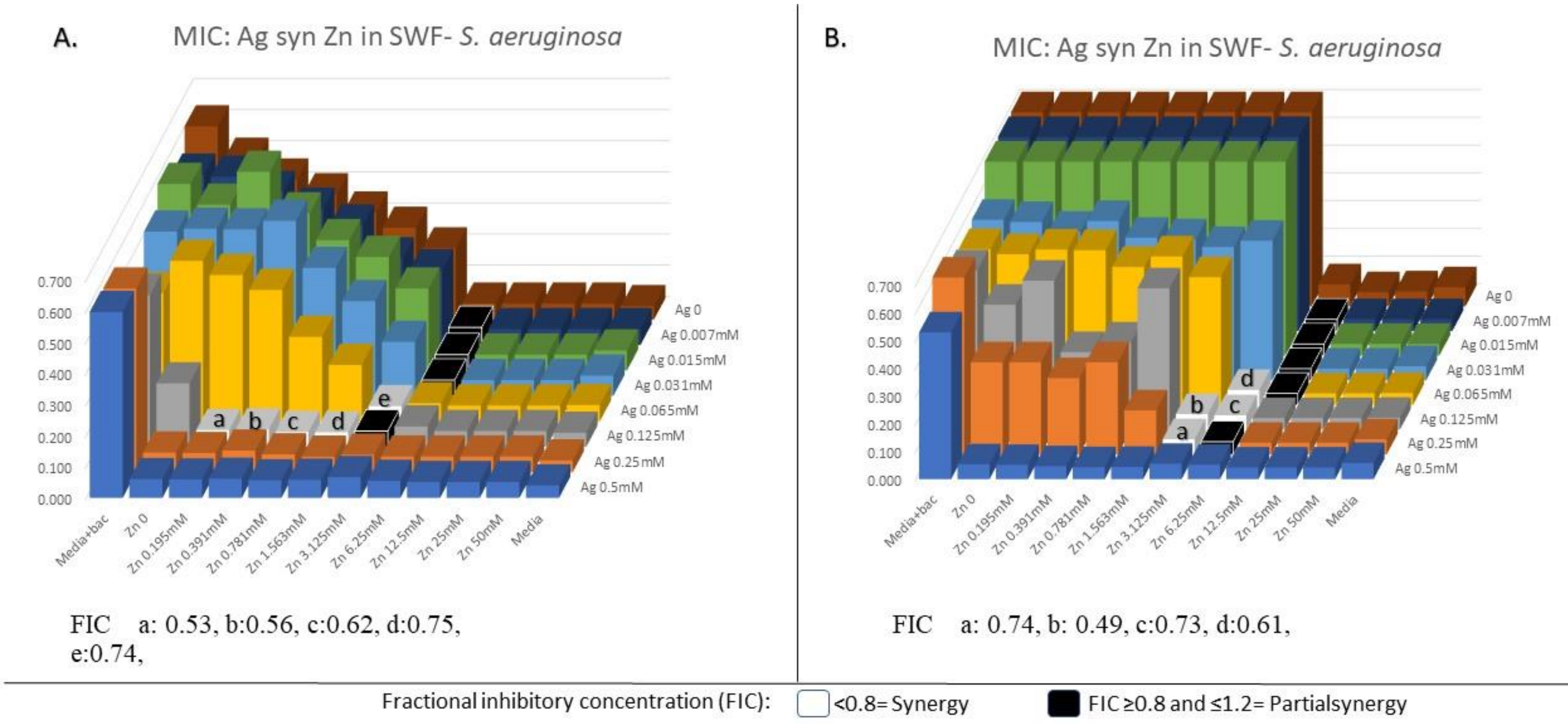

Figure S15. A. Minimum inhibitory concentration (MIC) and B. minimum bactericidal concentration (MBC) synergism (syn) effects of silver nitrate ($AgNO_3$) combined with zinc sulfate ($ZnSO_4$) against *P. aeruginosa* in SWF media.

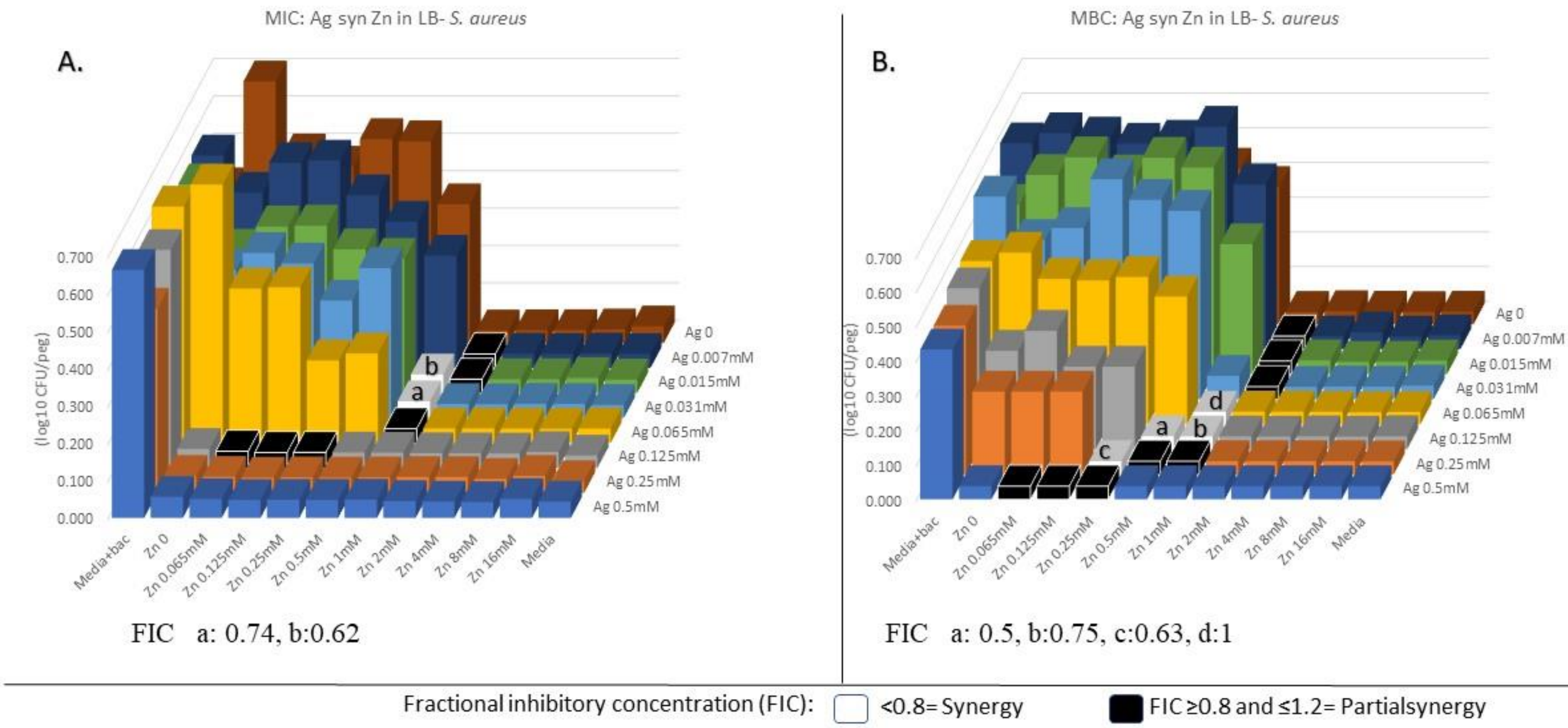

Figure S16. A. Minimum inhibitory concentration (MIC) and B. minimum bactericidal concentration (MBC) synergism (syn) effects of silver nitrate ($AgNO_3$) combined with zinc sulfate ($ZnSO_4$) against *S. aureus* in LB media.

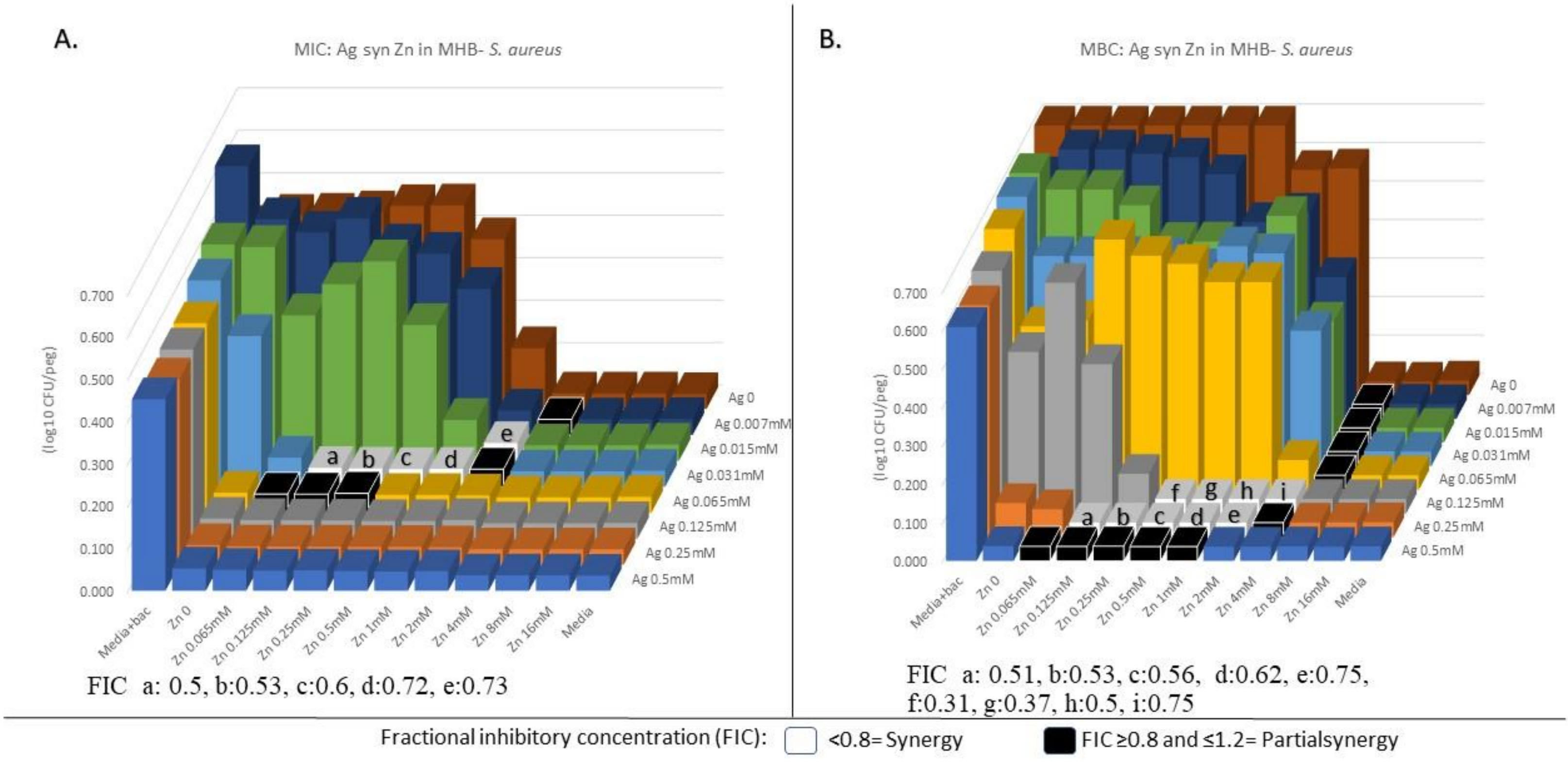

Figure S17. A. Minimum inhibitory concentration (MIC) and B. minimum bactericidal concentration (MBC) synergism (syn) effects of silver nitrate ($AgNO_3$) combined with zinc sulfate ($ZnSO_4$) against *S. aureus* in MHB media.

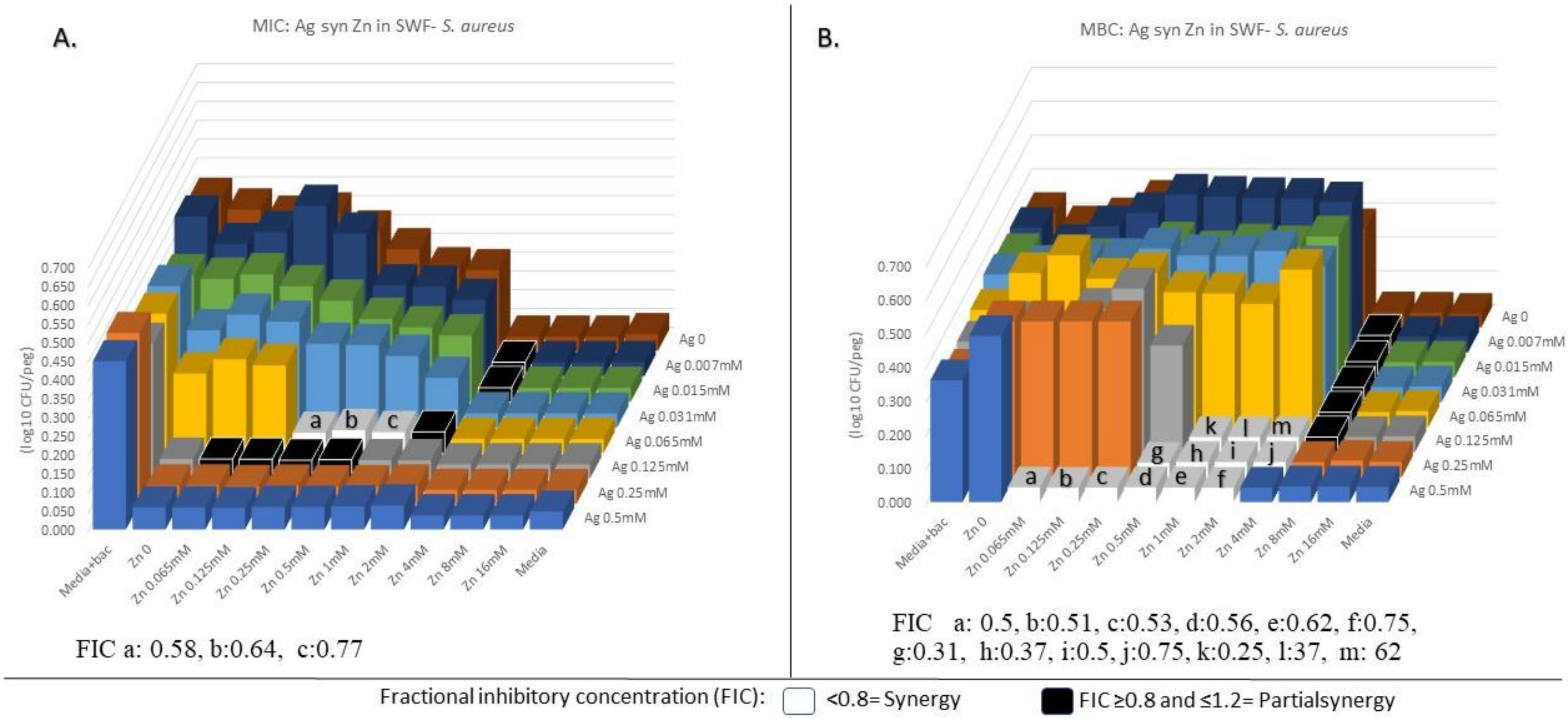

Figure S18. A. Minimum inhibitory concentration (MIC) and B. minimum bactericidal concentration (MBC) synergism (syn) effects of silver nitrate ($AgNO_3$) combined with zinc sulfate ($ZnSO_4$•) against *S. aureus* in SWF media.

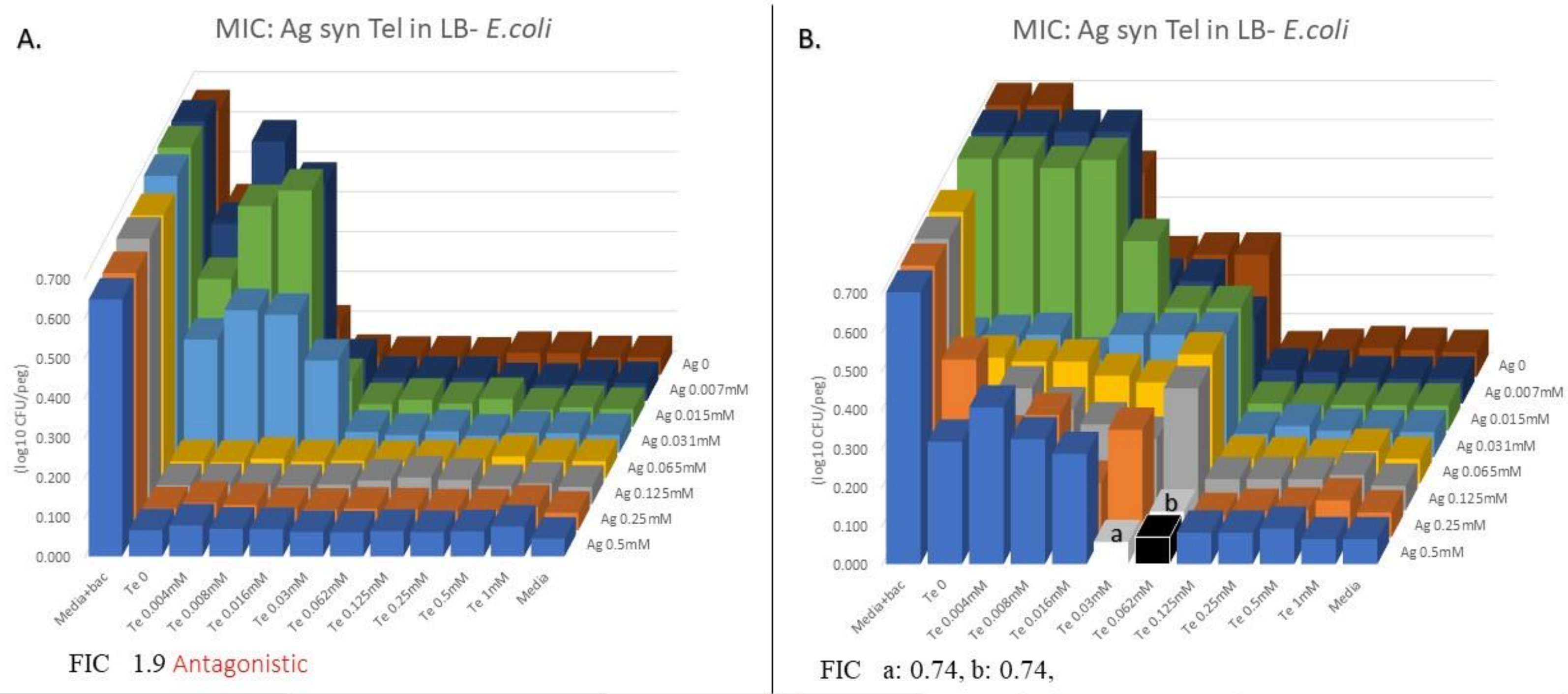

FIC 1.9 Antagonistic

FIC a: 0.74, b: 0.74,

Fractional inhibitory concentration (FIC): <0.8= Synergy FIC ≥0.8 and ≤1.2= Partialsynergy

Figure S19. A. Minimum inhibitory concentration (MIC) and B. minimum bactericidal concentration (MBC) synergism (syn) effects of silver nitrate ($AgNO_3$) combined with potassium tellurite (K_2TeO_3) against *E. coli* in LB media.

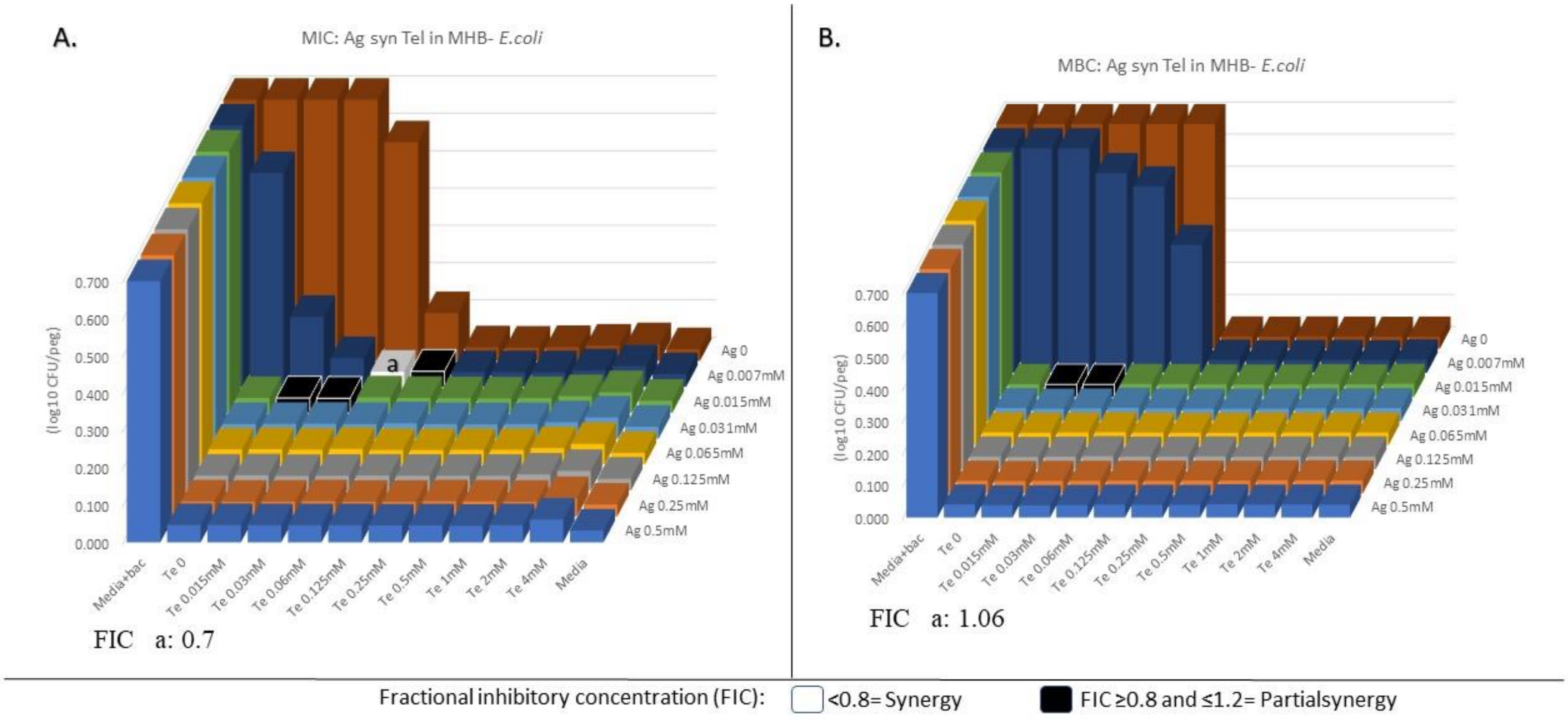

Figure S20. A. Minimum inhibitory concentration (MIC) and B. minimum bactericidal concentration (MBC) synergism (syn) effects of silver nitrate ($AgNO_3$) combined with potassium tellurite (K_2TeO_3) against *E. coli* in MHB media.

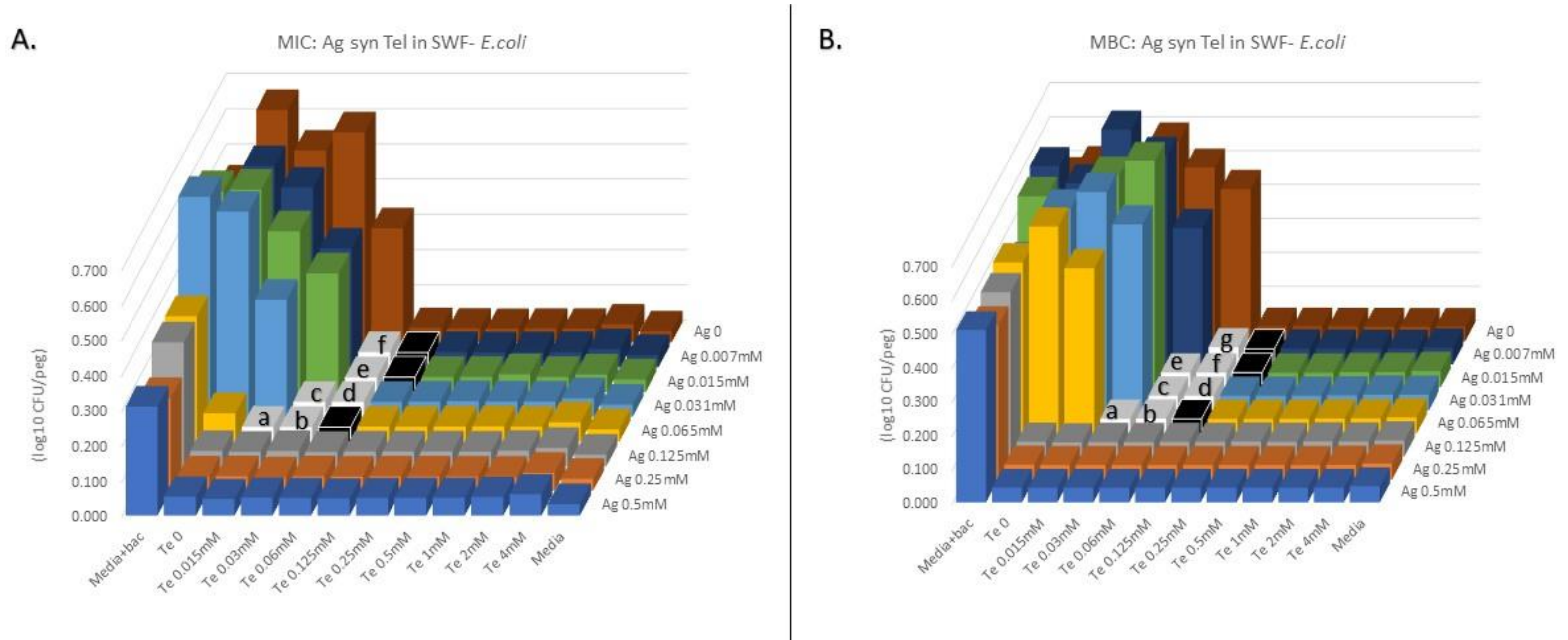

FIC a: 0.65, b:0.76, c:0.48, d:0.72, e:0.62, f:0.53

FIC a: 0.64, b:0.48, c:0.75, d:0.36, e:0.62, f:0.75, g:0.55

Fractional inhibitory concentration (FIC): <0.8= Synergy FIC ≥0.8 and ≤1.2= Partialsynergy

Figure S21. A. Minimum inhibitory concentration (MIC) and B. minimum bactericidal concentration (MBC) synergism (syn) effects of silver nitrate ($AgNO_3$) combined with tellurite (K_2TeO_3) against *E. coli* in simulated wound fluid (SWF) media.

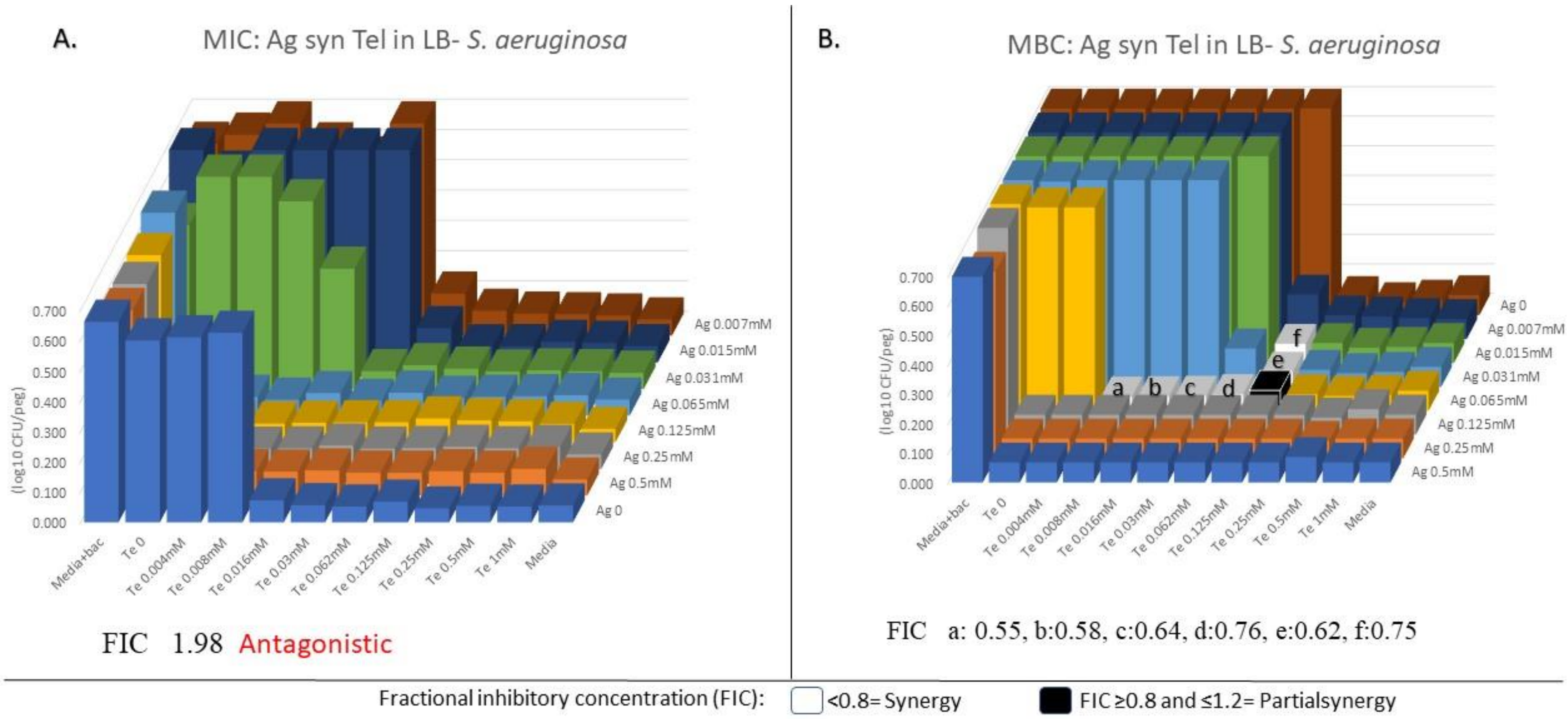

Figure S22. A. Minimum inhibitory concentration (MIC) and B. minimum bactericidal concentration (MBC) synergism (syn) effects of silver nitrate ($AgNO_3$) combined with tellurite (K_2TeO_3) against *P. aeruginosa* in LB media.

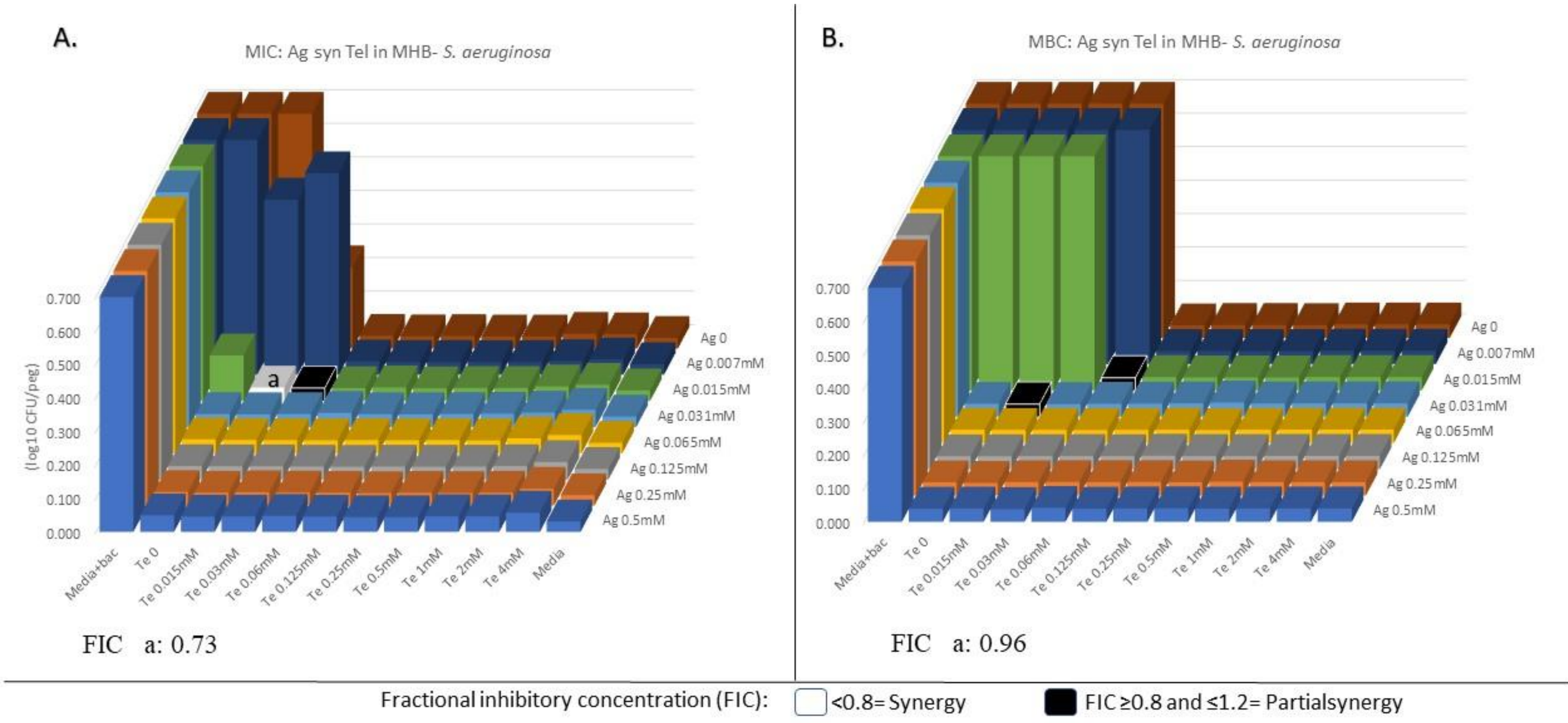

Figure S23. A. Minimum inhibitory concentration (MIC) and B. minimum bactericidal concentration (MBC) synergism (syn) effects of silver nitrate ($AgNO_3$) combined with tellurite (K_2TeO_3) against *P. aeruginosa* in MHB media.

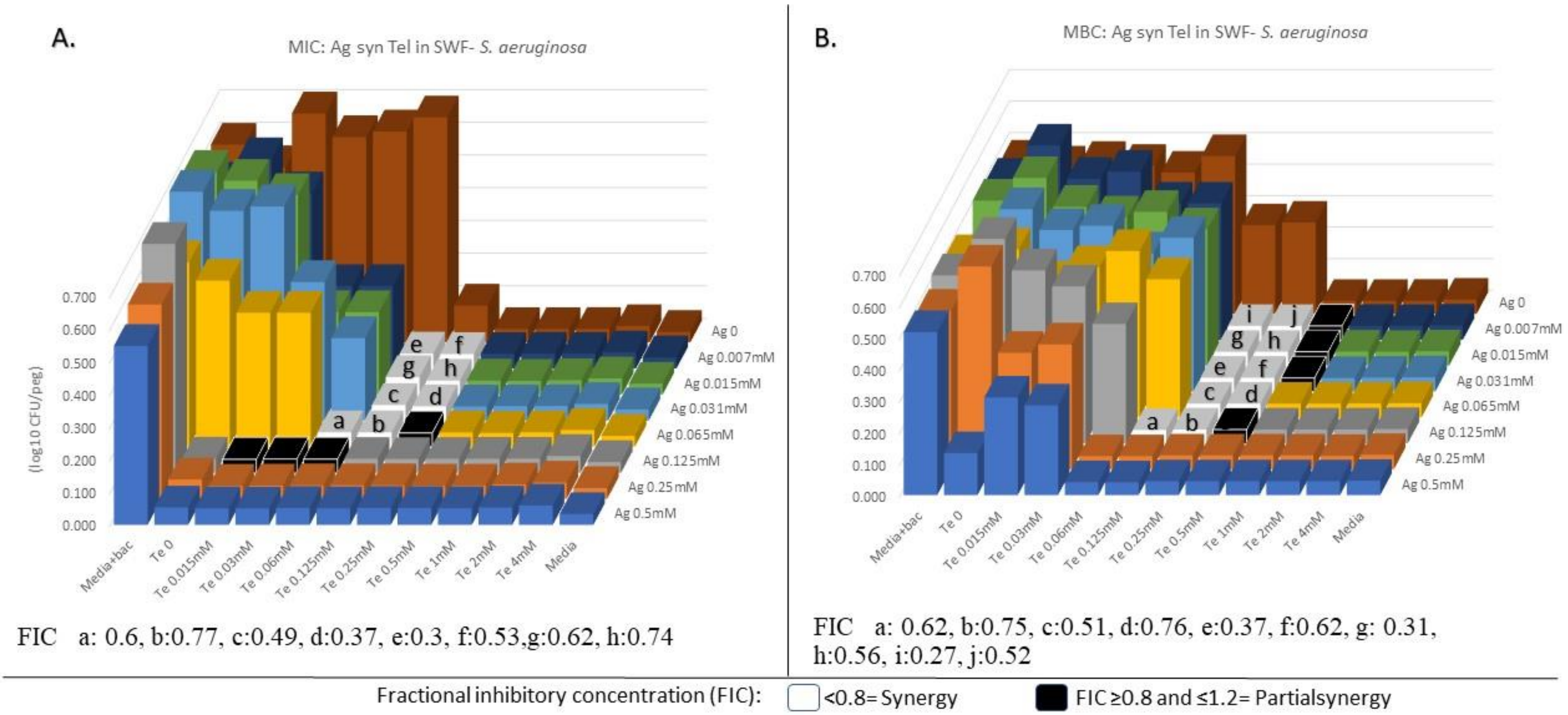

FIC a: 0.6, b:0.77, c:0.49, d:0.37, e:0.3, f:0.53,g:0.62, h:0.74

FIC a: 0.62, b:0.75, c:0.51, d:0.76, e:0.37, f:0.62, g: 0.31, h:0.56, i:0.27, j:0.52

Fractional inhibitory concentration (FIC): <0.8= Synergy FIC ≥0.8 and ≤1.2= Partialsynergy

Figure S24. A. Minimum inhibitory concentration (MIC) and B. minimum bactericidal concentration (MBC) synergism (syn) effects of silver nitrate ($AgNO_3$) combined with tellurite (K_2TeO_3) against *P. aeruginosa* in SWF media.

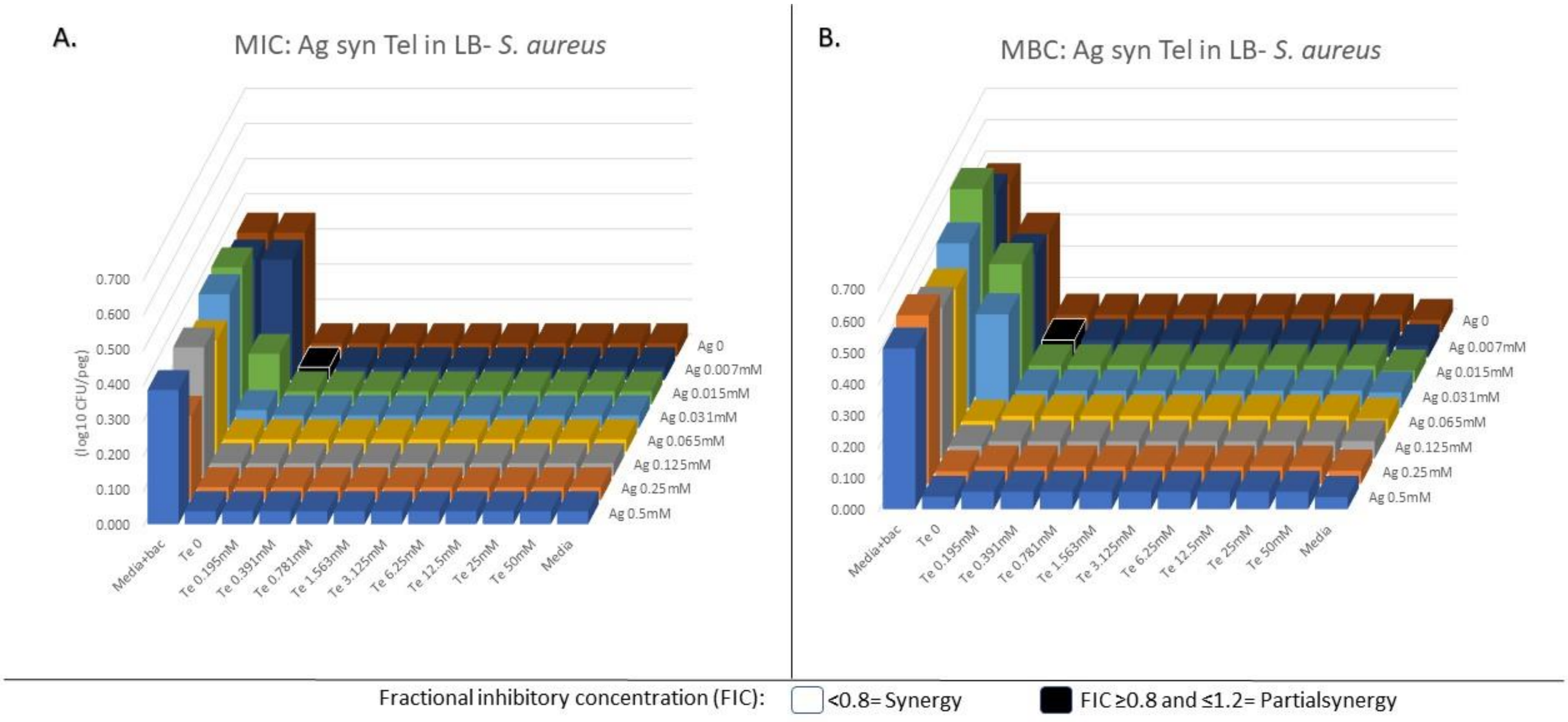

Figure S25. A. Minimum inhibitory concentration (MIC) and B. minimum bactericidal concentration (MBC) synergism (syn) effects of silver nitrate ($AgNO_3$) combined with tellurite (K_2TeO_3) against *S. aureus* in LB media.

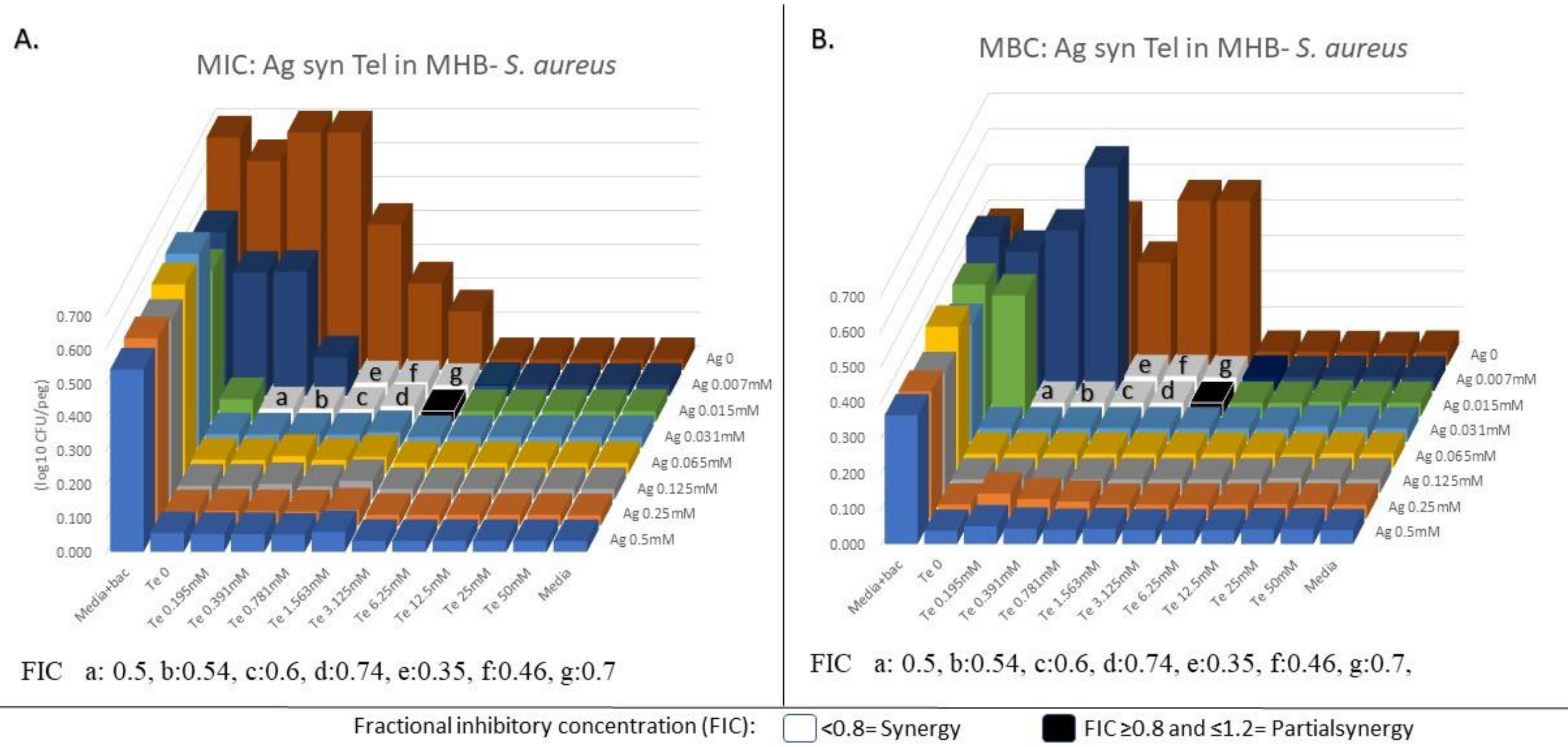

Figure S26. A. Minimum inhibitory concentration (MIC) and B. minimum bactericidal concentration (MBC) synergism (syn) effects of silver nitrate ($AgNO_3$) combined with tellurite (K_2TeO_3) against *S. aureus* in MHB media.

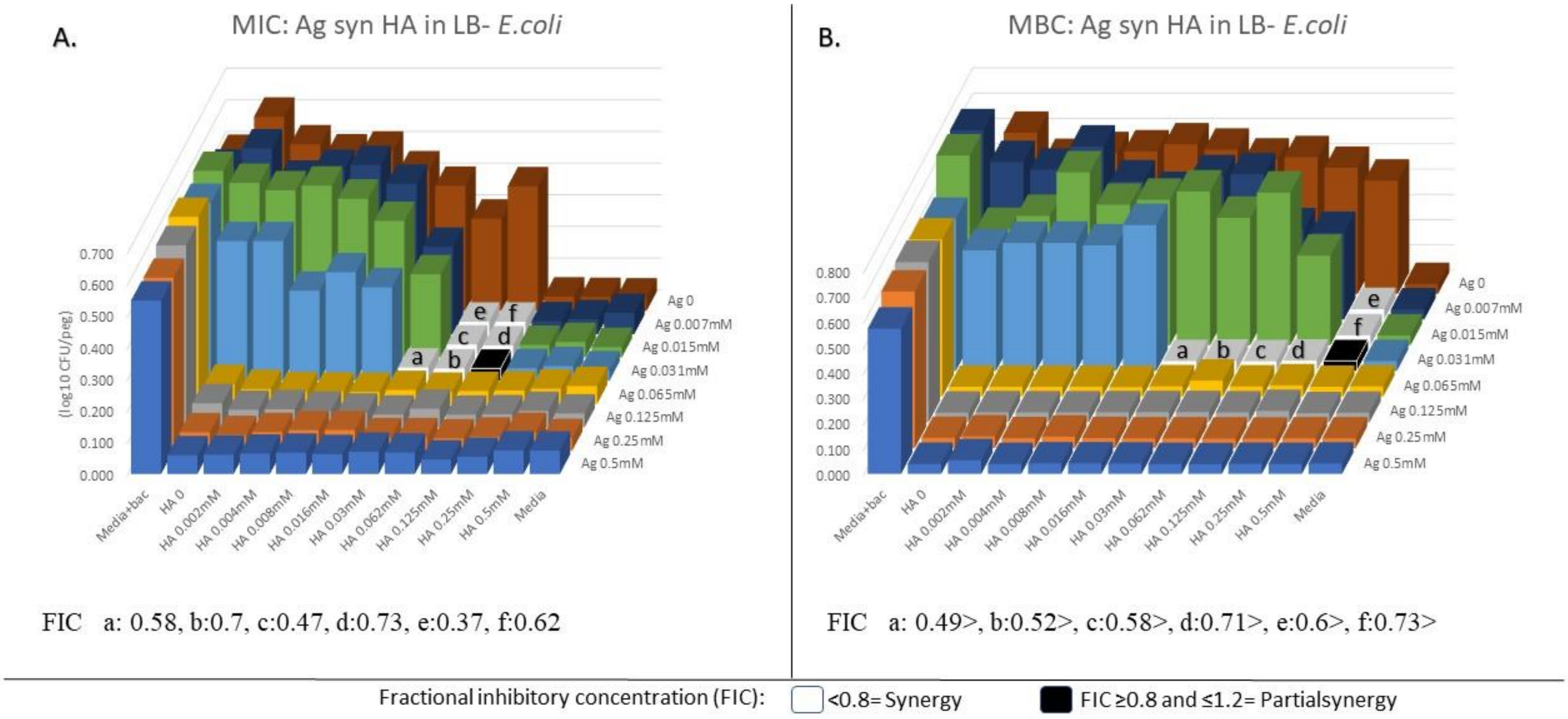

Figure S27. A. Minimum inhibitory concentration (MIC) and B. minimum bactericidal concentration (MBC) synergism (syn) effects of silver nitrate ($AgNO_3$) combined with tetrachloroaurate (iii) (HA, $AuCl_4$) against *E. coli* in LB media.

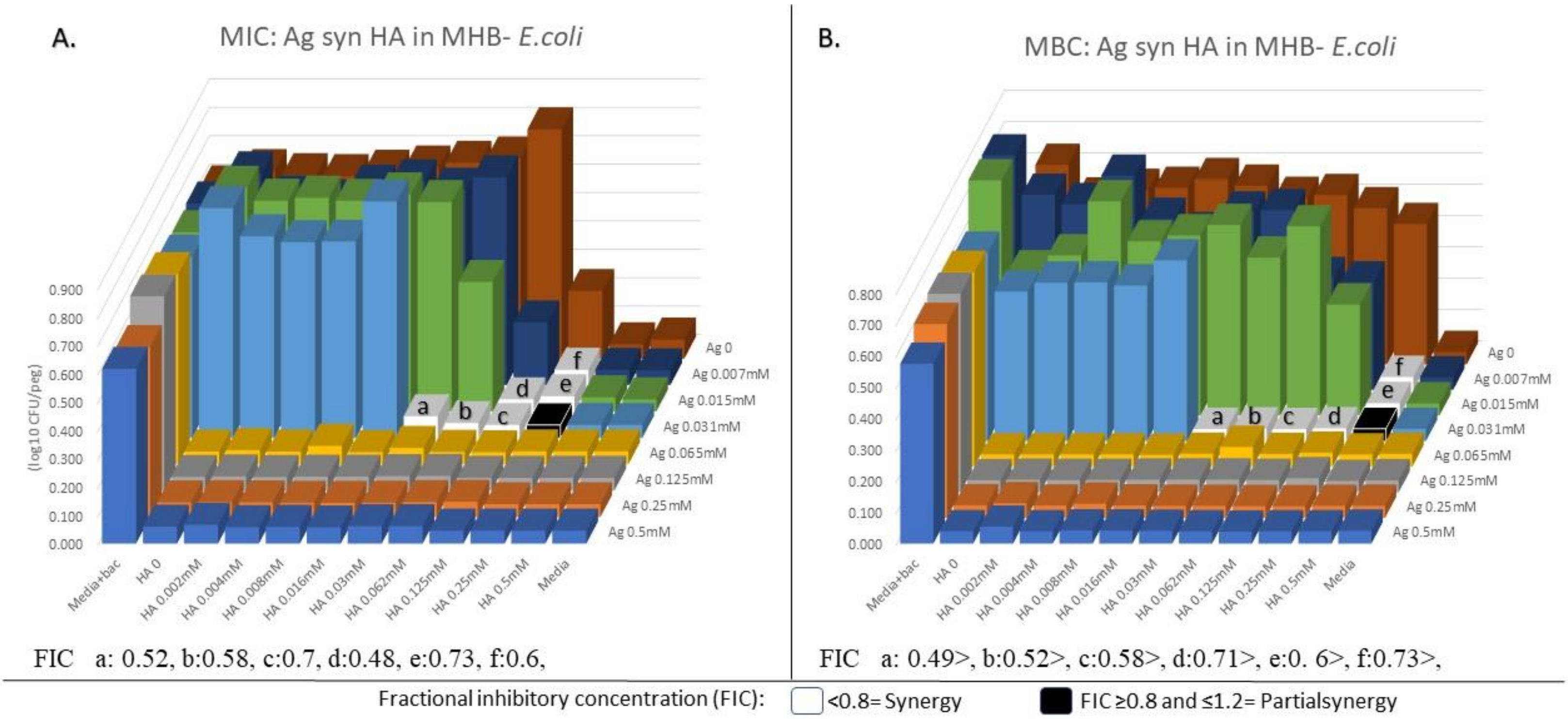

FIC a: 0.52, b:0.58, c:0.7, d:0.48, e:0.73, f:0.6,

FIC a: 0.49>, b:0.52>, c:0.58>, d:0.71>, e:0. 6>, f:0.73>,

Fractional inhibitory concentration (FIC): <0.8= Synergy FIC ≥0.8 and ≤1.2= Partialsynergy

Figure S28. A. Minimum inhibitory concentration (MIC) and B. minimum bactericidal concentration (MBC) synergism (syn) effects of silver nitrate ($AgNO_3$) combined with tetrachloroaurate (iii) (HA, $AuCl_4$) against *E. coli* in MHB media.

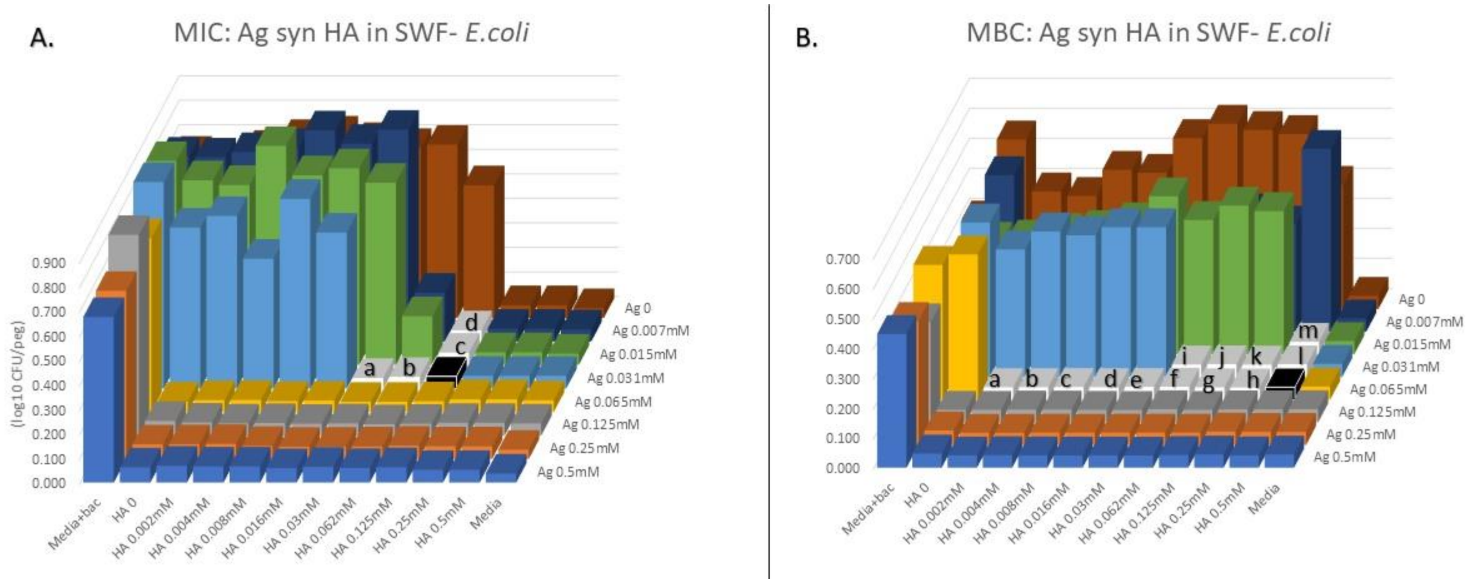

FIC a: 0.58, b:0.7, c:0.7, d:0.48, e:0.73,

FIC a: 0.522>, b:0.524>, c:0.528>, d:0.53>, e:0.55>, f:0.58>, g:0.64>, h:0.77>, i:0.3>, j:0.36>, k:0.49>, l:74>, m: 0.62>,

Fractional inhibitory concentration (FIC): □ <0.8= Synergy ■ FIC ≥0.8 and ≤1.2= Partialsynergy

Figure S29. A. Minimum inhibitory concentration (MIC) and B. minimum bactericidal concentration (MBC) synergism (syn) effects of silver nitrate ($AgNO_3$) combined with tetrachloroaurate (iii) (HA, $AuCl_4$) against *E. coli* in simulated wound fluid (SWF) media.

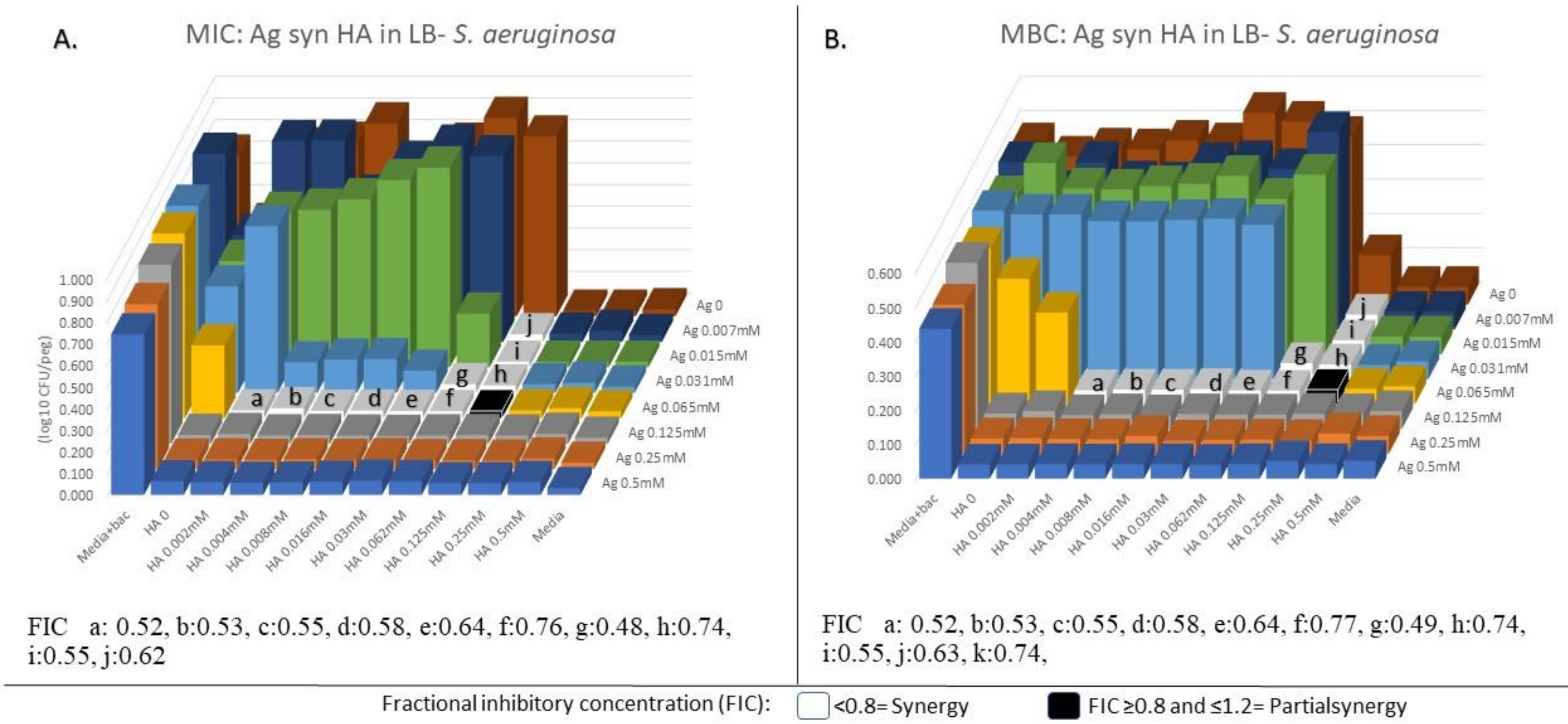

Figure S30. A. Minimum inhibitory concentration (MIC) and B. minimum bactericidal concentration (MBC) synergism (syn) effects of silver nitrate ($AgNO_3$) combined with tetrachloroaurate (iii) (HA, $AuCl_4$) against *P. aeruginosa* in LB media.

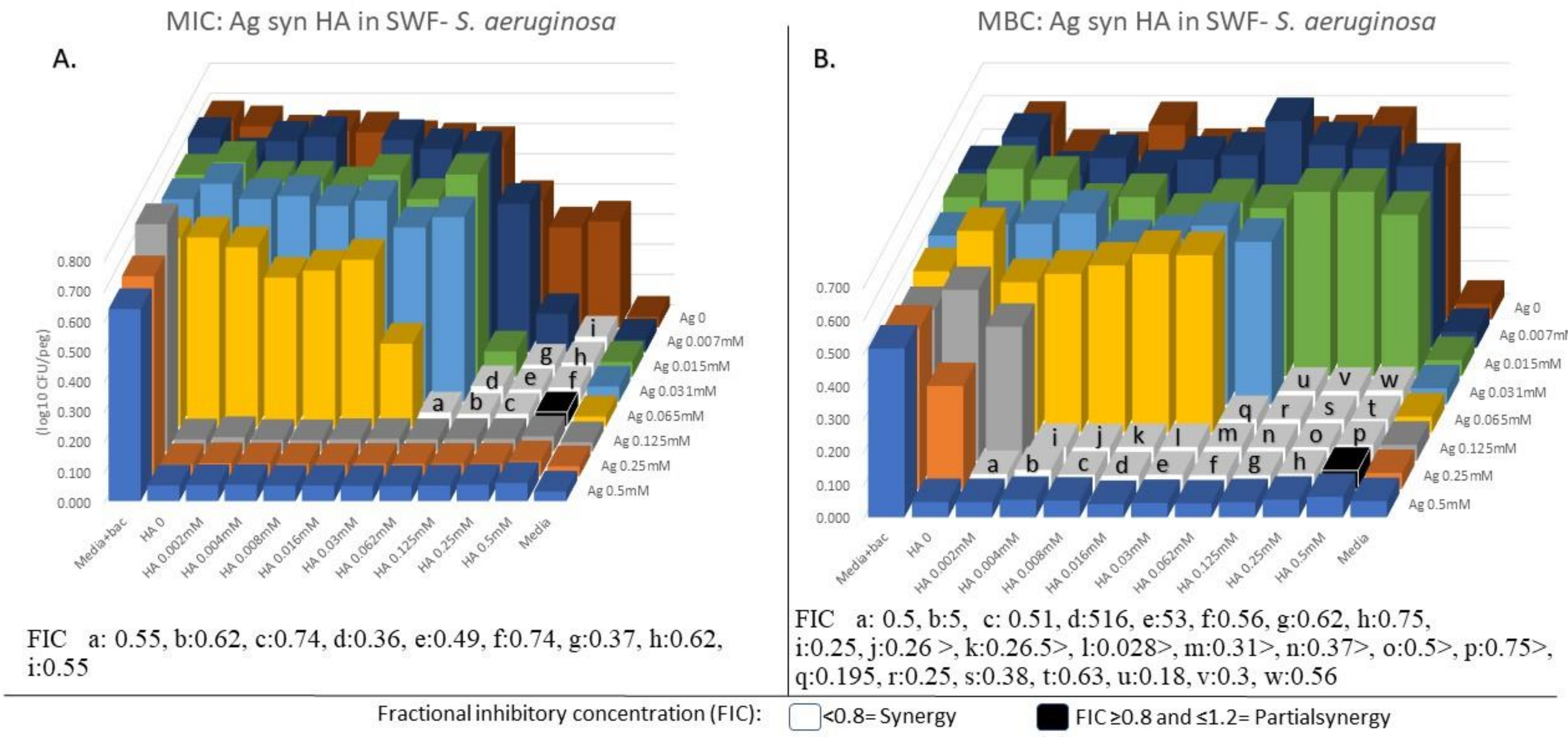

Figure S31. A. Minimum inhibitory concentration (MIC) and B. minimum bactericidal concentration (MBC) synergism (syn) effects of silver nitrate ($AgNO_3$) combined with tetrachloroaurate (iii) (HA, $AuCl_4$) against *P. aeruginosa* in SWF media.

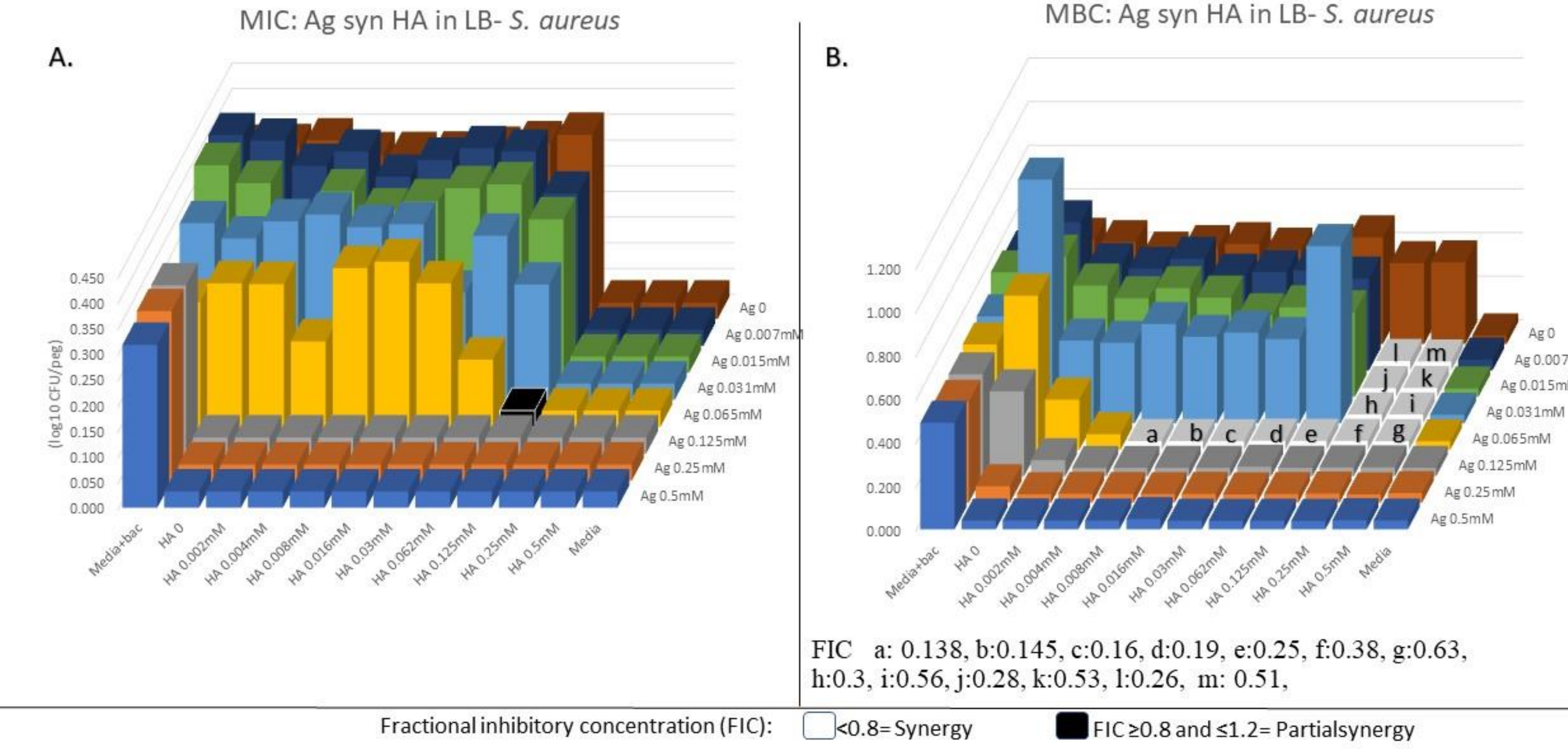

Figure S32. A. Minimum inhibitory concentration (MIC) and B. minimum bactericidal concentration (MBC) synergism (syn) effects of silver nitrate ($AgNO_3$) combined with tetrachloroaurate (iii) (HA, $AuCl_4$) against *S. aureus* in LB media.

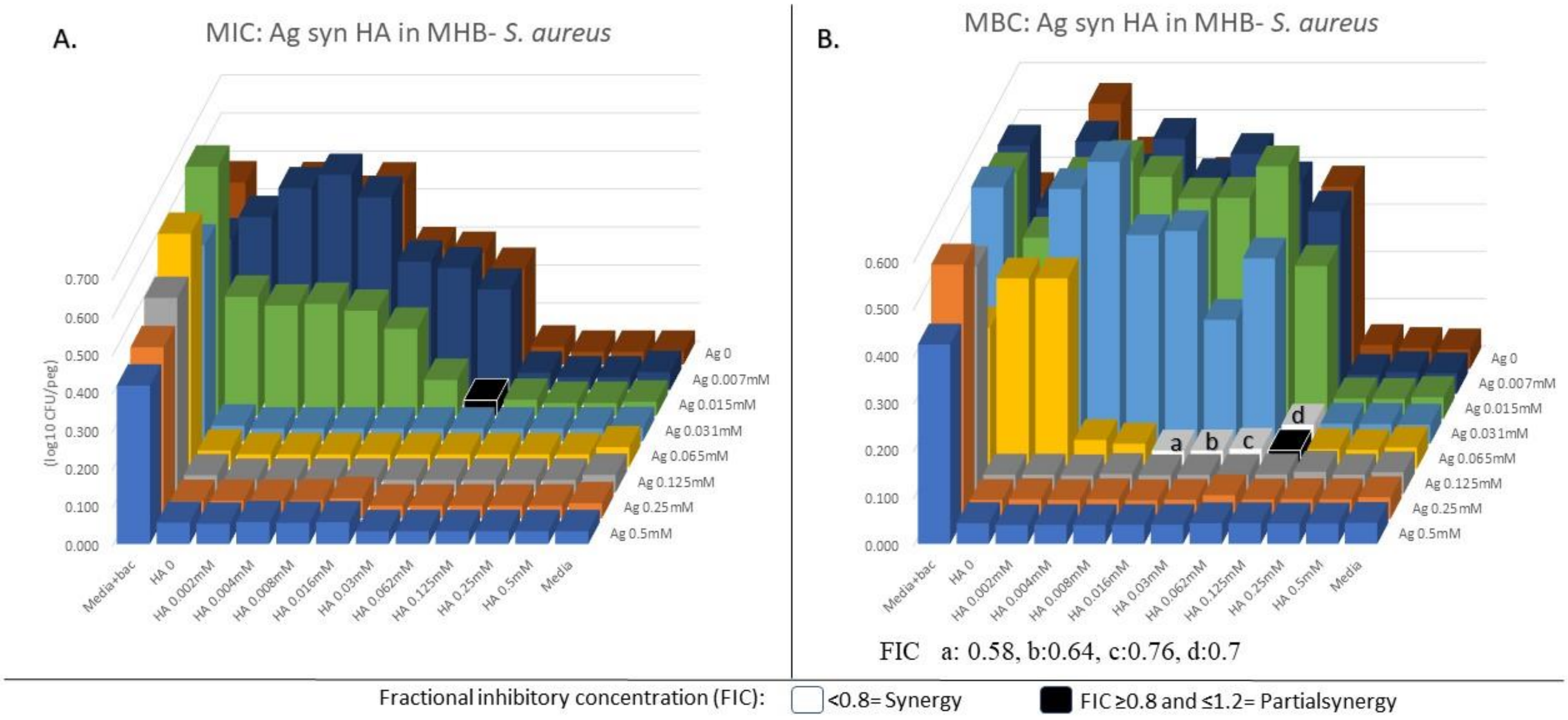

Figure S33. A. Minimum inhibitory concentration (MIC) and B. minimum bactericidal concentration (MBC) synergism (syn) effects of silver nitrate ($AgNO_3$) combined with tetrachloroaurate (iii) (HA, $AuCl_4$) against *S. aureus* in MHB media.

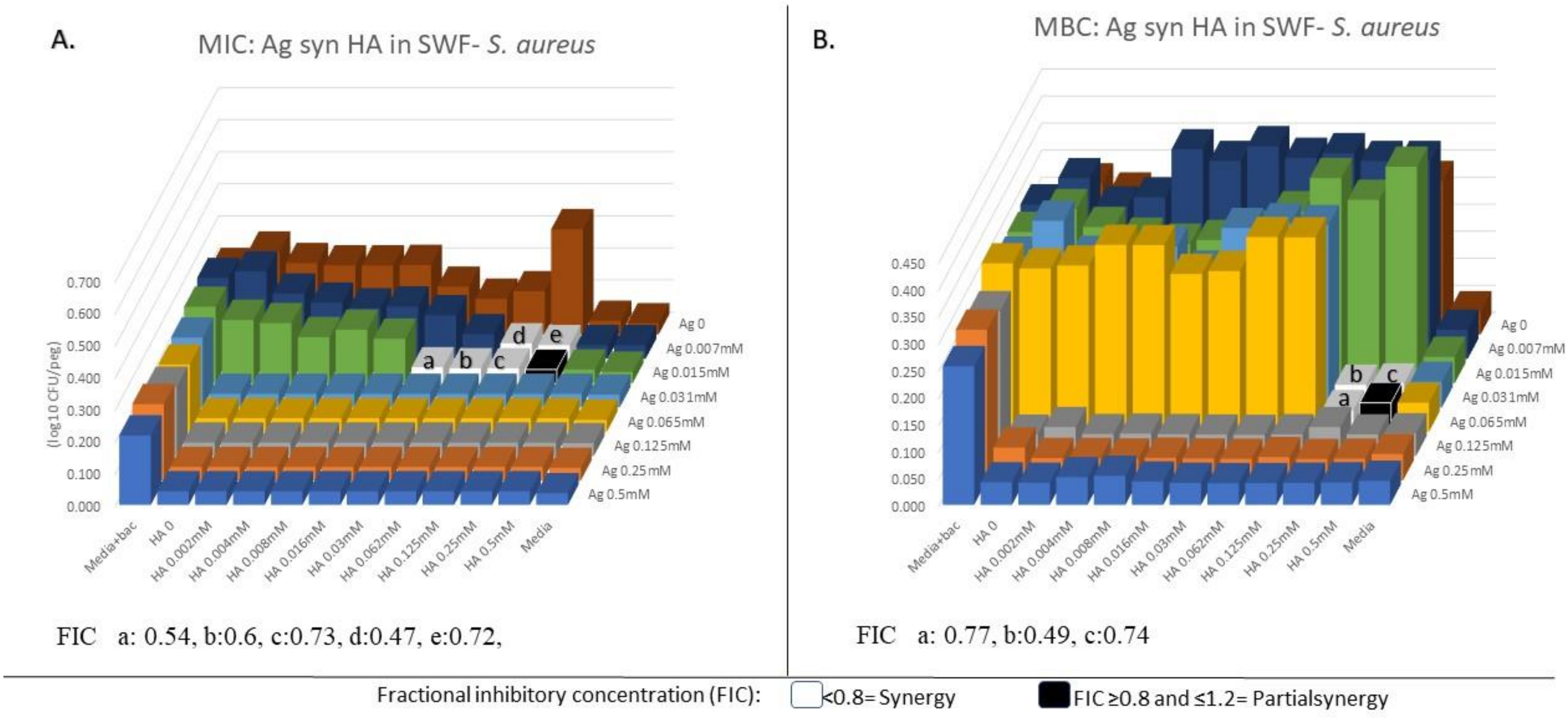

Figure S34. A. Minimum inhibitory concentration (MIC) and B. minimum bactericidal concentration (MBC) synergism (syn) effects of silver nitrate ($AgNO_3$) combined with tetrachloroaurate (iii) (HA, $AuCl_4$) against *S. aureus* in SWF media.

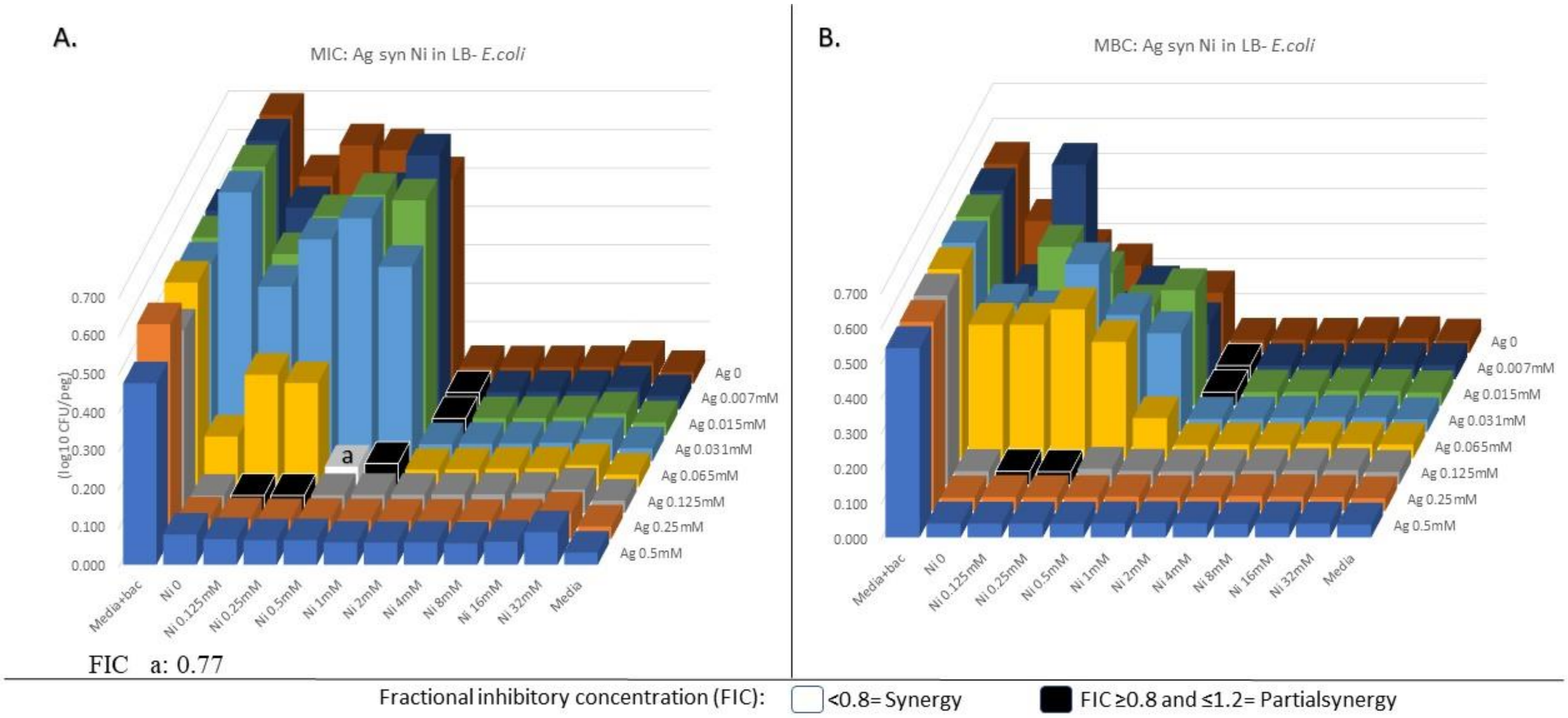

Figure S35. A. Minimum inhibitory concentration (MIC) and B. minimum bactericidal concentration (MBC) synergism (syn) effects of silver nitrate ($AgNO_3$) combined with nickel sulfate ($NiSO_4$) against *E. coli* in LB media.

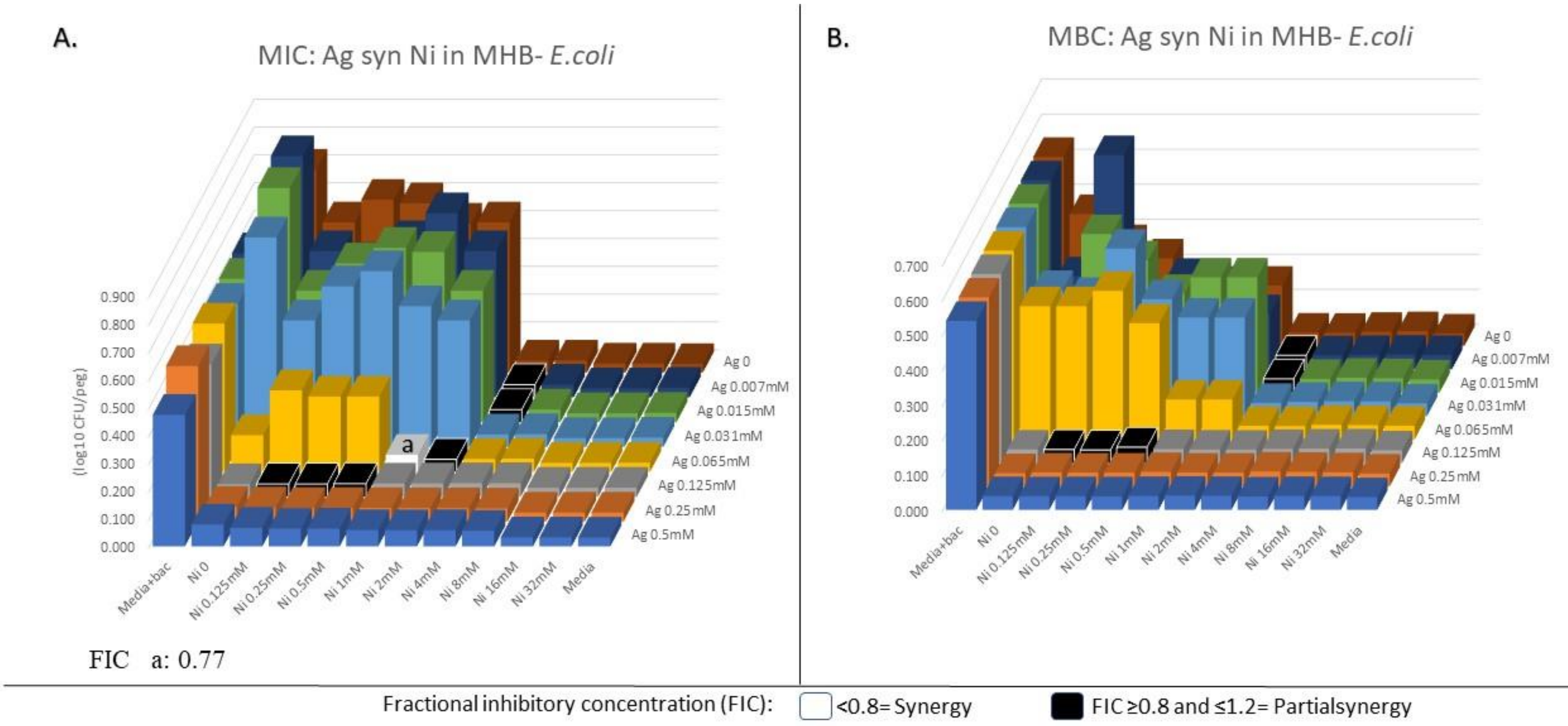

Figure S36. A. Minimum inhibitory concentration (MIC) and B. minimum bactericidal concentration (MBC) synergism (syn) effects of silver nitrate ($AgNO_3$) combined with nickel sulfate ($NiSO_4$) against *E. coli* in MHB media.

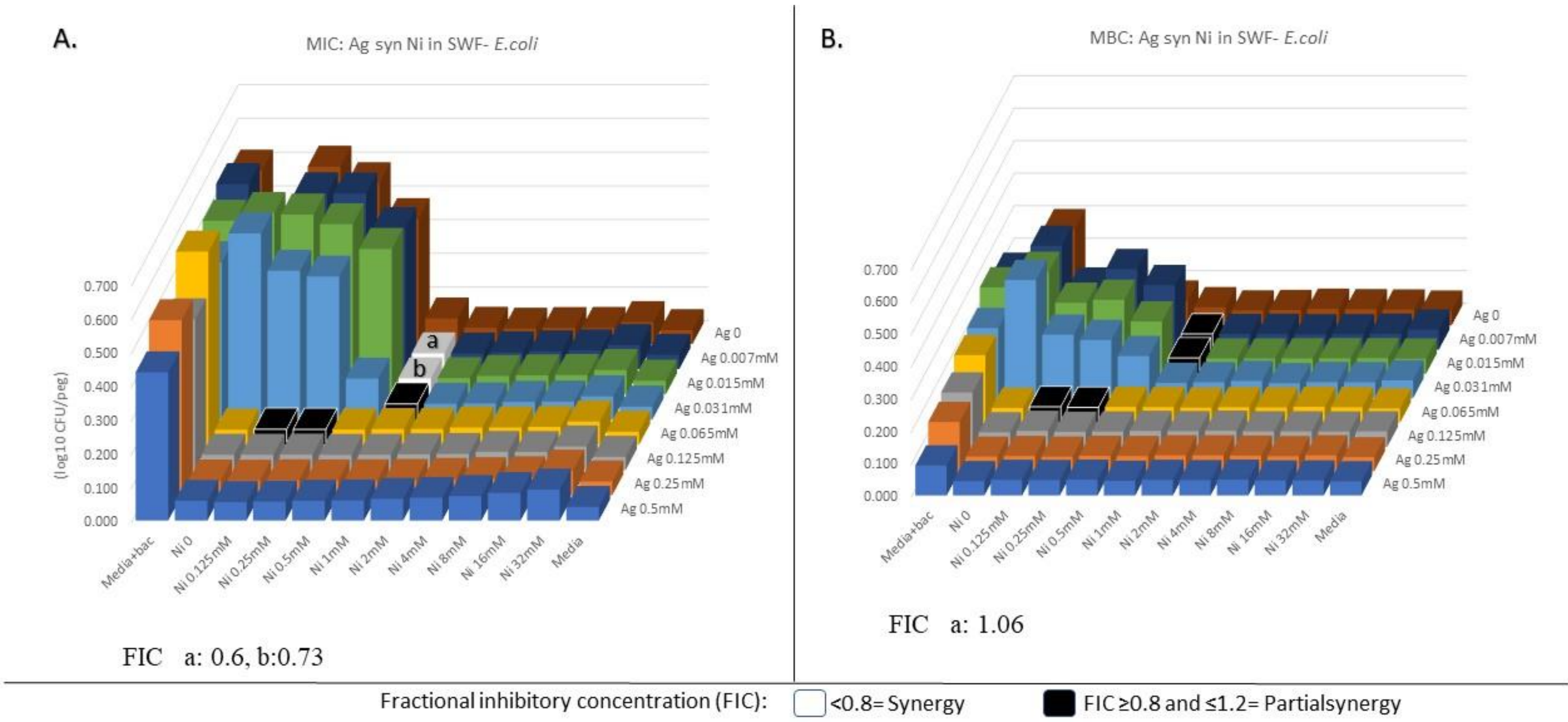

Figure S37. A. Minimum inhibitory concentration (MIC) and B. minimum bactericidal concentration (MBC) synergism (syn) effects of silver nitrate ($AgNO_3$) combined with nickel sulfate ($NiSO_4$) against *E. coli* in simulated wound fluid (SWF) media.

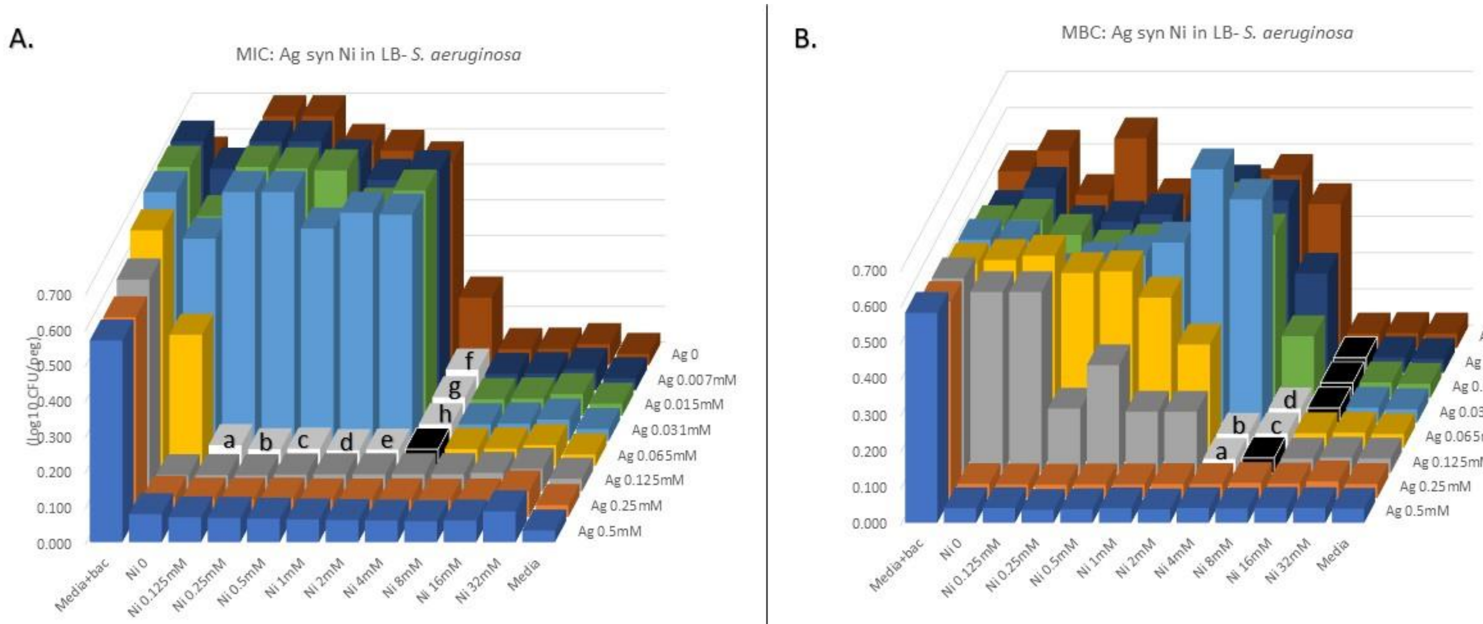

FIC a: 0.55, b:0.58, c:0.64, d:0.77, e:79, f:0.55, g:0.62, h:0.74

FIC a: 0.75, b:0.51, c:0.76, d:0.62

Fractional inhibitory concentration (FIC): <0.8= Synergy FIC ≥0.8 and ≤1.2= Partialsynergy

Figure S38. A. Minimum inhibitory concentration (MIC) and B. minimum bactericidal concentration (MBC) synergism (syn) effects of silver nitrate ($AgNO_3$) combined with nickel sulfate ($NiSO_4$) against *P. aeruginosa* in LB media.

A.

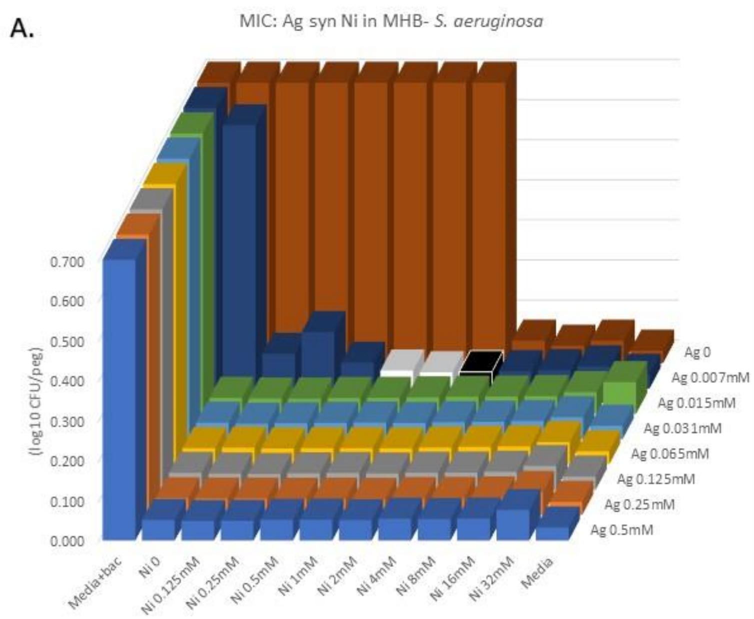

FIC a: 0.50, b:0.71,

B.

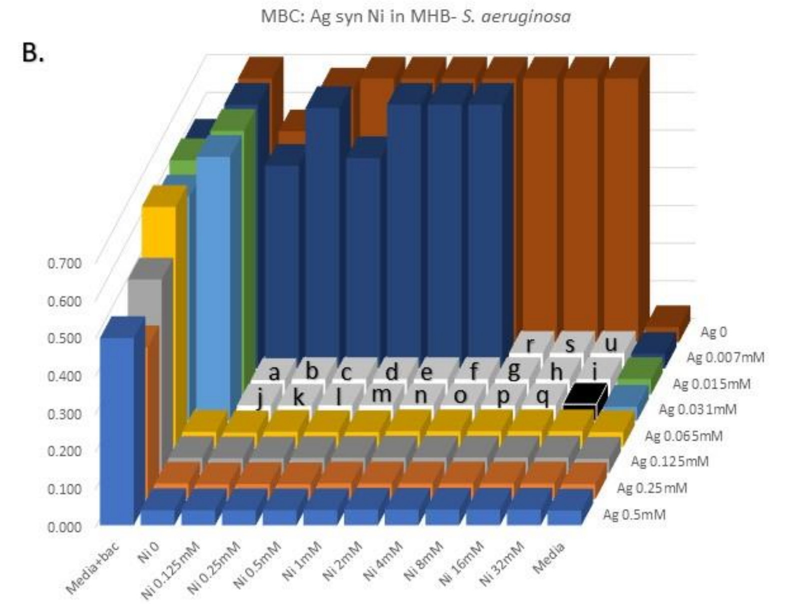

FIC a: 0.23, b:0.23, c:0.24, d:0.246, e:0.26, f:0.29, g:0.35, h:0.48, i:0.73, j:0.47, k:0.48, l:484, m: 0.49, n:50, o:54, p:0.6, q:0.72, r:0.23, s:0.35, u:0.6

Fractional inhibitory concentration (FIC): <0.8= Synergy FIC ≥0.8 and ≤1.2= Partialsynergy

Figure S39. A. Minimum inhibitory concentration (MIC) and B. minimum bactericidal concentration (MBC) synergism (syn) effects of silver nitrate ($AgNO_3$) combined with nickel sulfate ($NiSO_4$) against *P. aeruginosa* in MHB media.

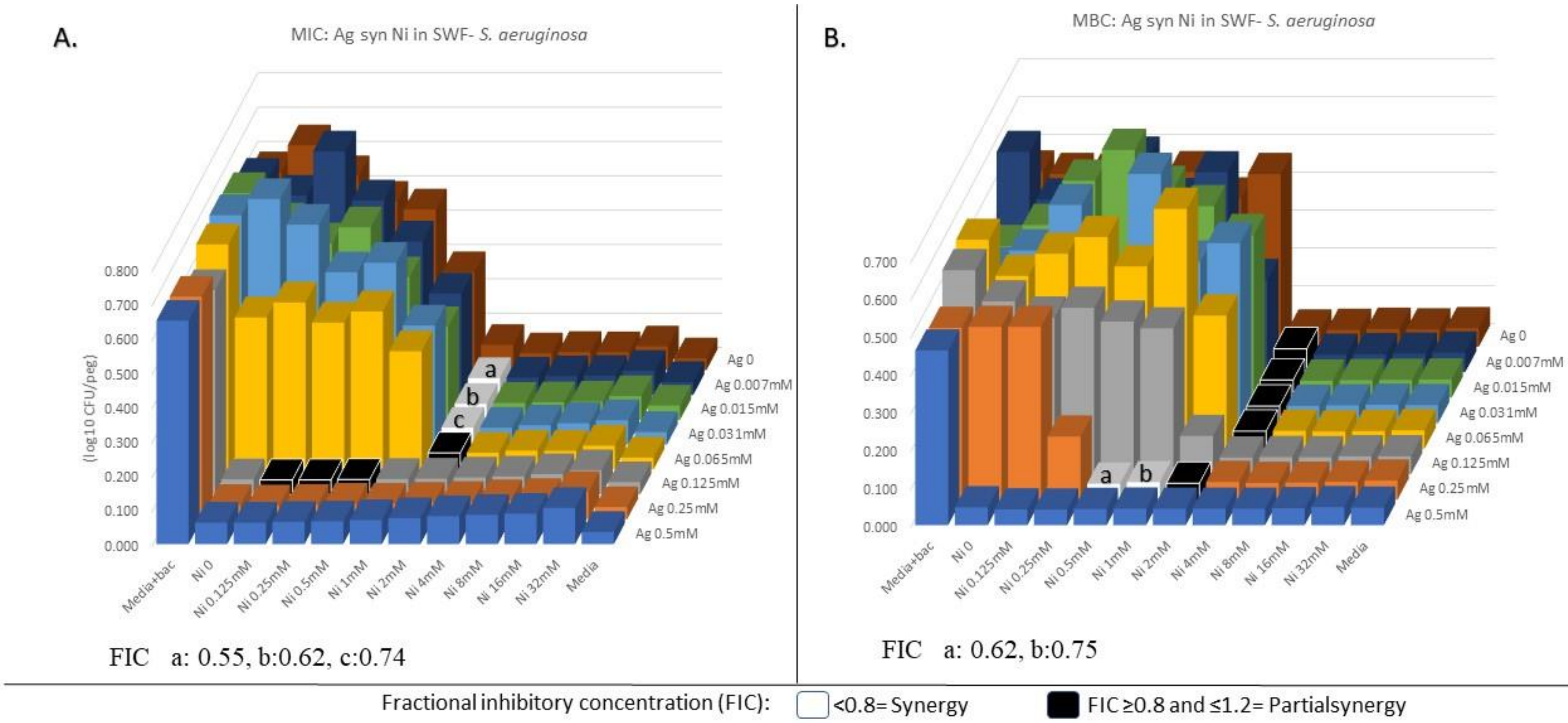

Figure S40. A. Minimum inhibitory concentration (MIC) and B. minimum bactericidal concentration (MBC) synergism (syn) effects of silver nitrate ($AgNO_3$) combined with nickel sulfate ($NiSO_4$) against *P. aeruginosa* in SWF media.

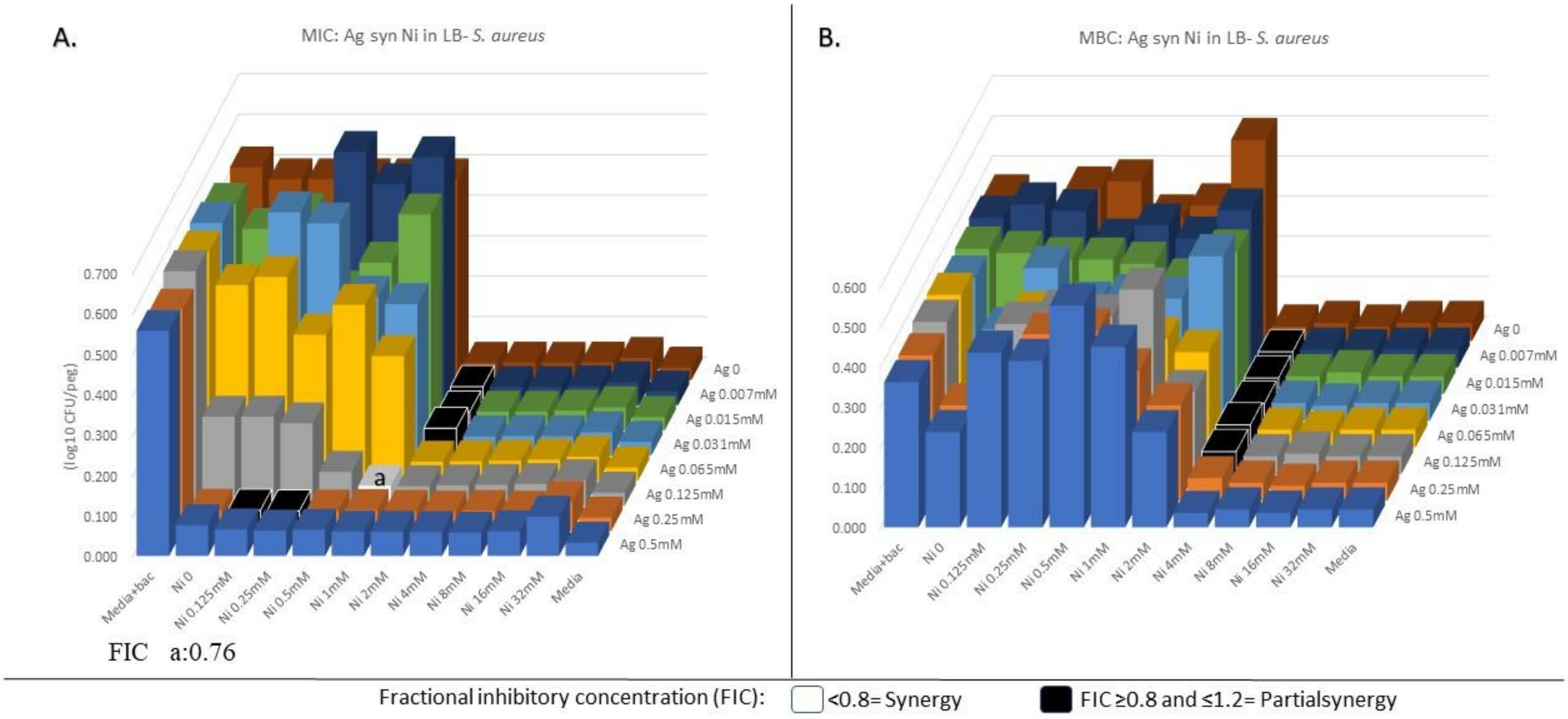

Figure S41. A. Minimum inhibitory concentration (MIC) and B. minimum bactericidal concentration (MBC) synergism (syn) effects of silver nitrate ($AgNO_3$) combined with nickel sulfate ($NiSO_4$) against *S. aureus* in LB media.

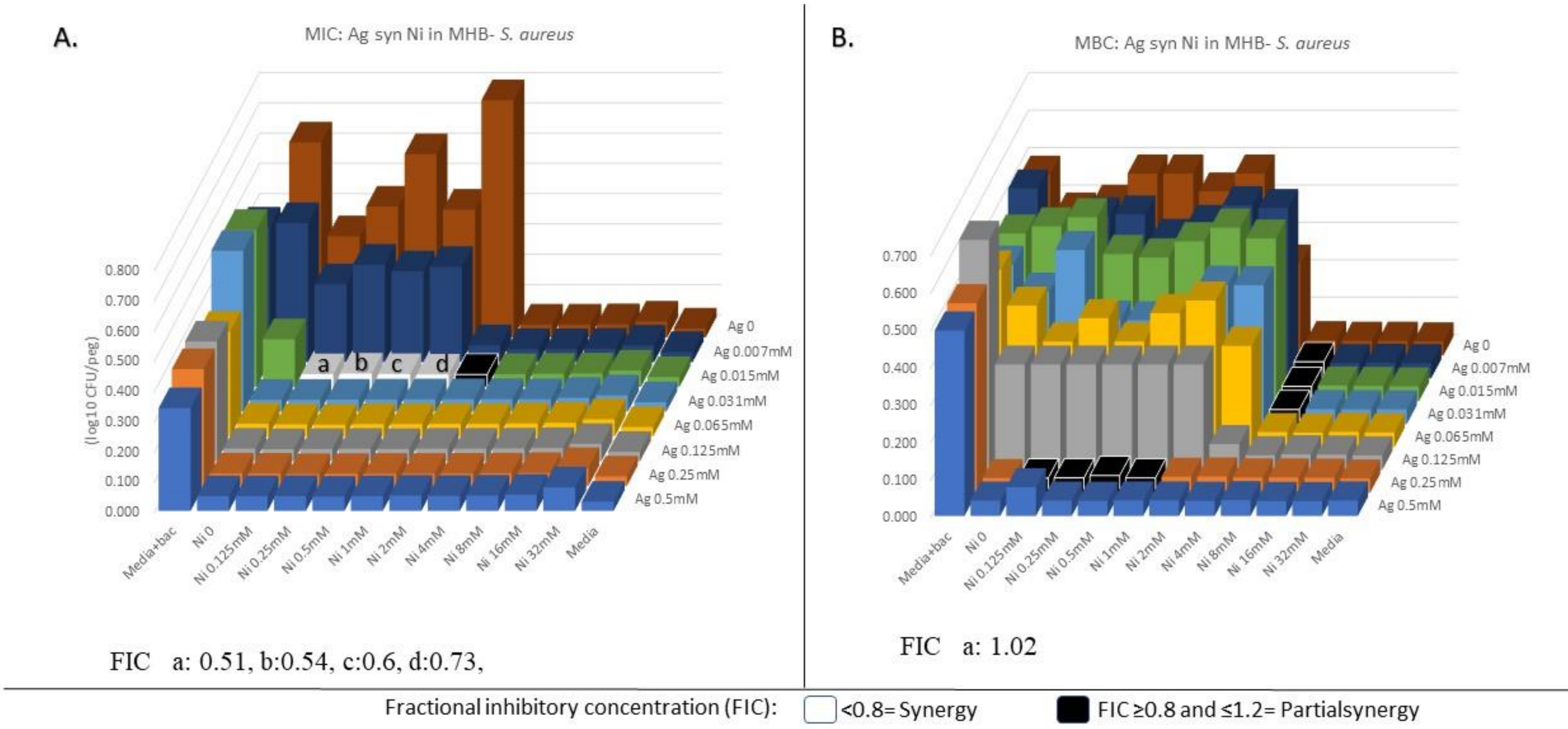

Figure S42. A. Minimum inhibitory concentration (MIC) and B. minimum bactericidal concentration (MBC) synergism (syn) effects of silver nitrate ($AgNO_3$) combined with nickel sulfate ($NiSO_4$) against *S. aureus* in MHB media.

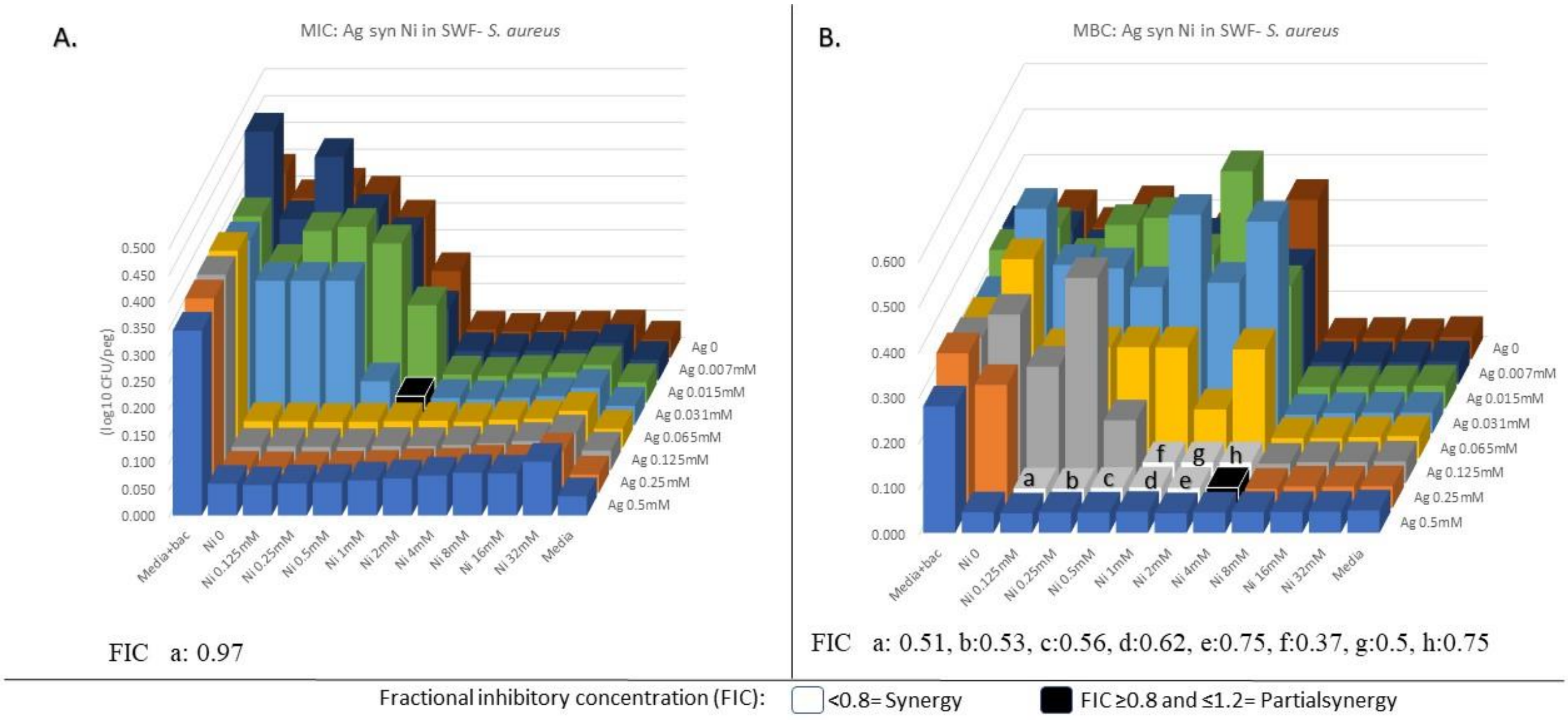

Figure S43. A. Minimum inhibitory concentration (MIC) and B. minimum bactericidal concentration (MBC) synergism (syn) effects of silver nitrate ($AgNO_3$) combined with nickel sulfate ($NiSO_4$) against *S. aureus* in SWF media.

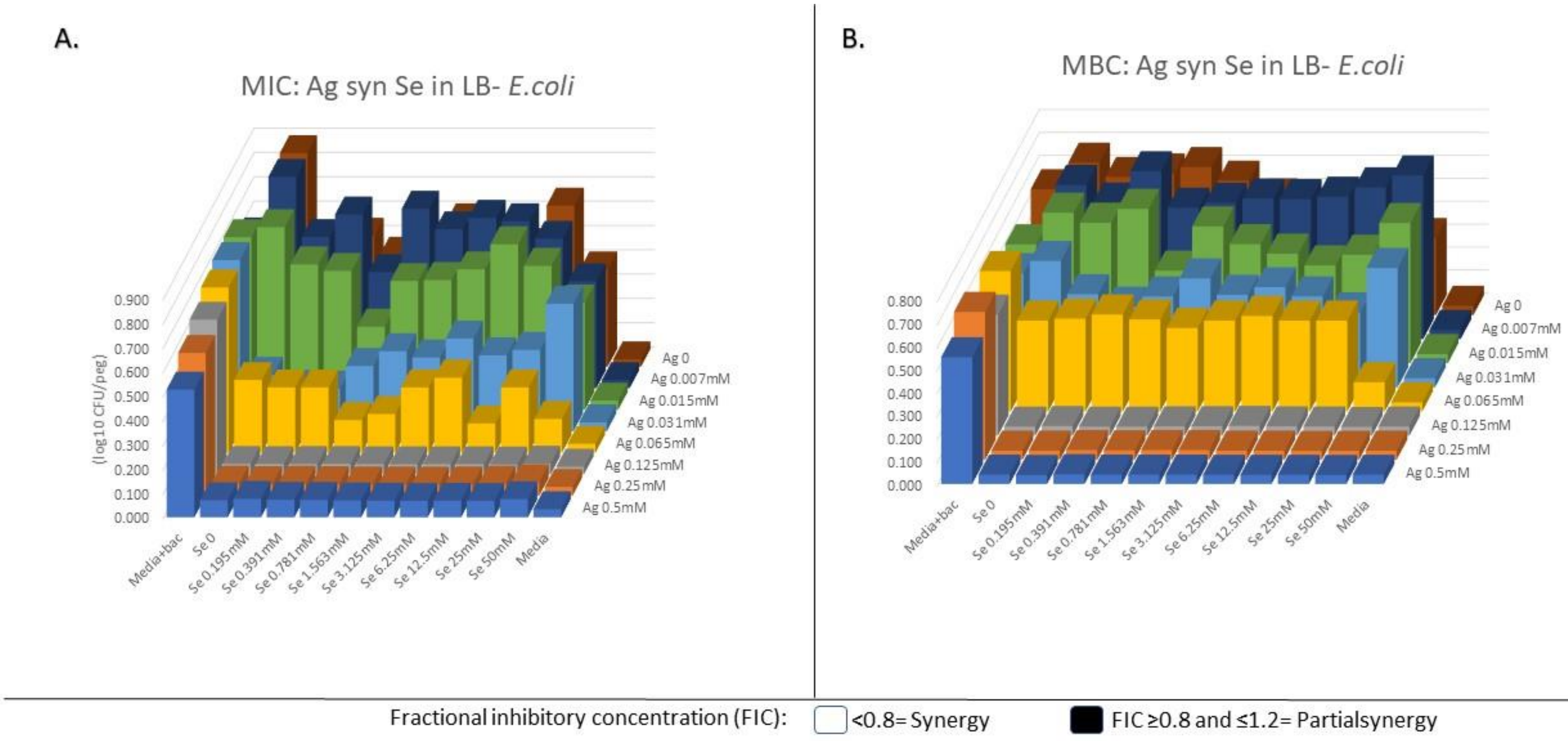

Figure S44. A. Minimum inhibitory concentration (MIC) and B. minimum bactericidal concentration (MBC) synergism (syn) effects of silver nitrate ($AgNO_3$) combined with sodium selenite (Na_2SeO_3) against *E. coli* in LB media.

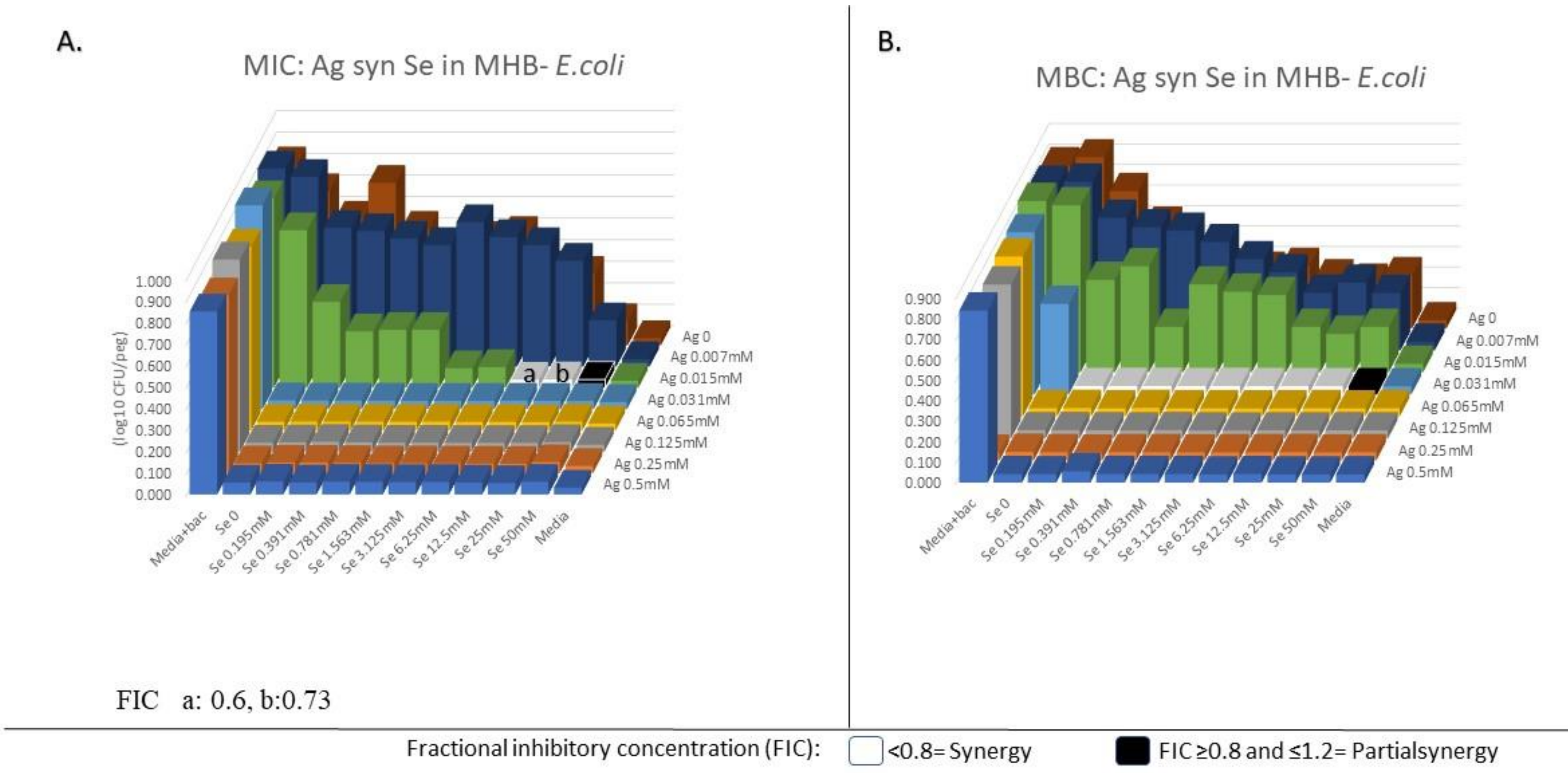

Figure S45. A. Minimum inhibitory concentration (MIC) and B. minimum bactericidal concentration (MBC) synergism (syn) effects of silver nitrate ($AgNO_3$) combined with sodium selenite (Na_2SeO_3) against *E. coli* in MHB media.

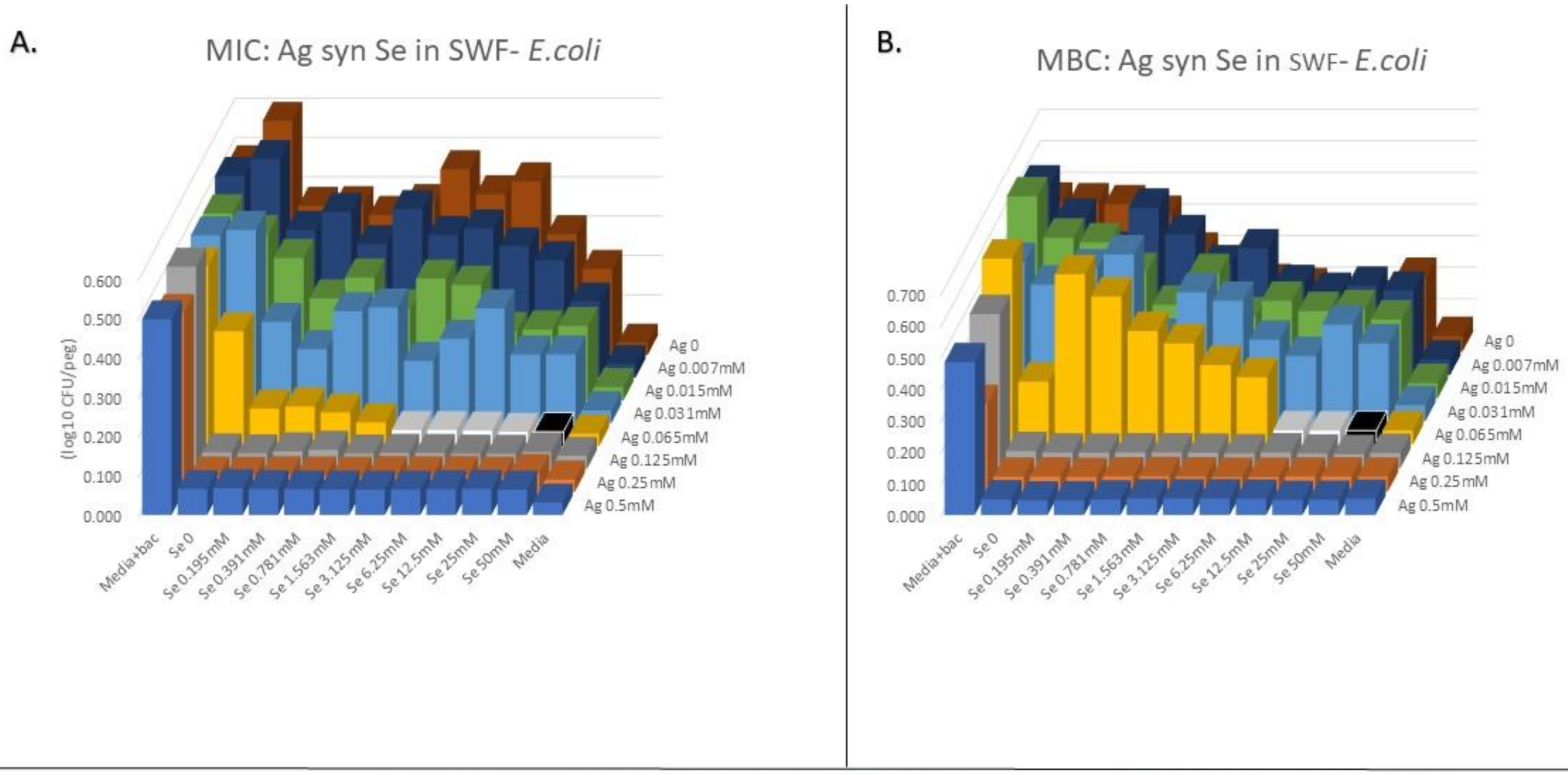

Fractional inhibitory concentration (FIC): <0.8= Synergy FIC ≥0.8 and ≤1.2= Partialsynergy

Figure S46. A. Minimum inhibitory concentration (MIC) and B. minimum bactericidal concentration (MBC) synergism (syn) effects of silver nitrate ($AgNO_3$) combined with sodium selenite (Na_2SeO_3) against *E. coli* in simulated wound fluid (SWF) media.

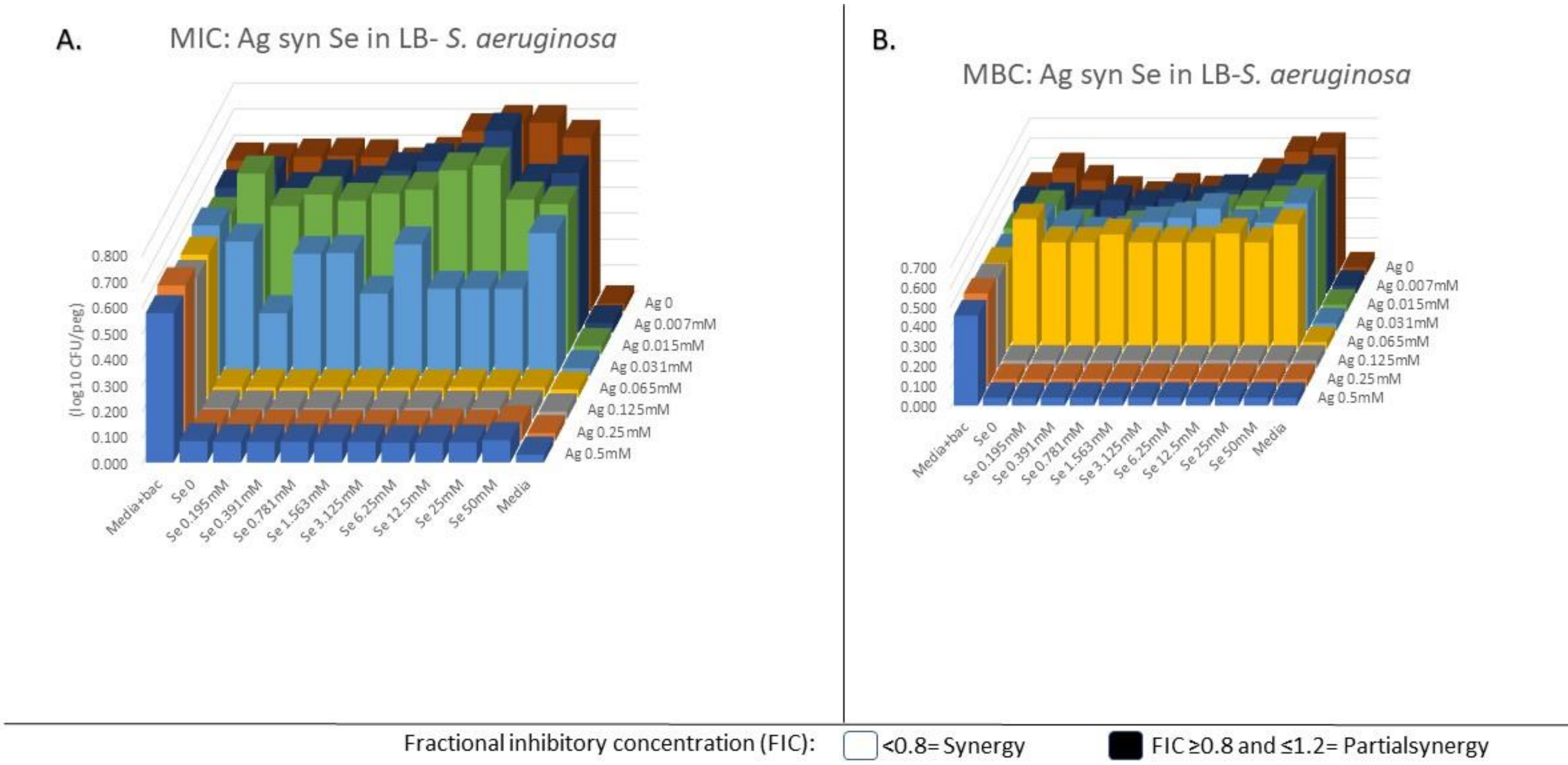

Figure S47. A. Minimum inhibitory concentration (MIC) and B. minimum bactericidal concentration (MBC) synergism (syn) effects of silver nitrate ($AgNO_3$) combined with sodium selenite (Na_2SeO_3) against *P. aeruginosa* in LB media.

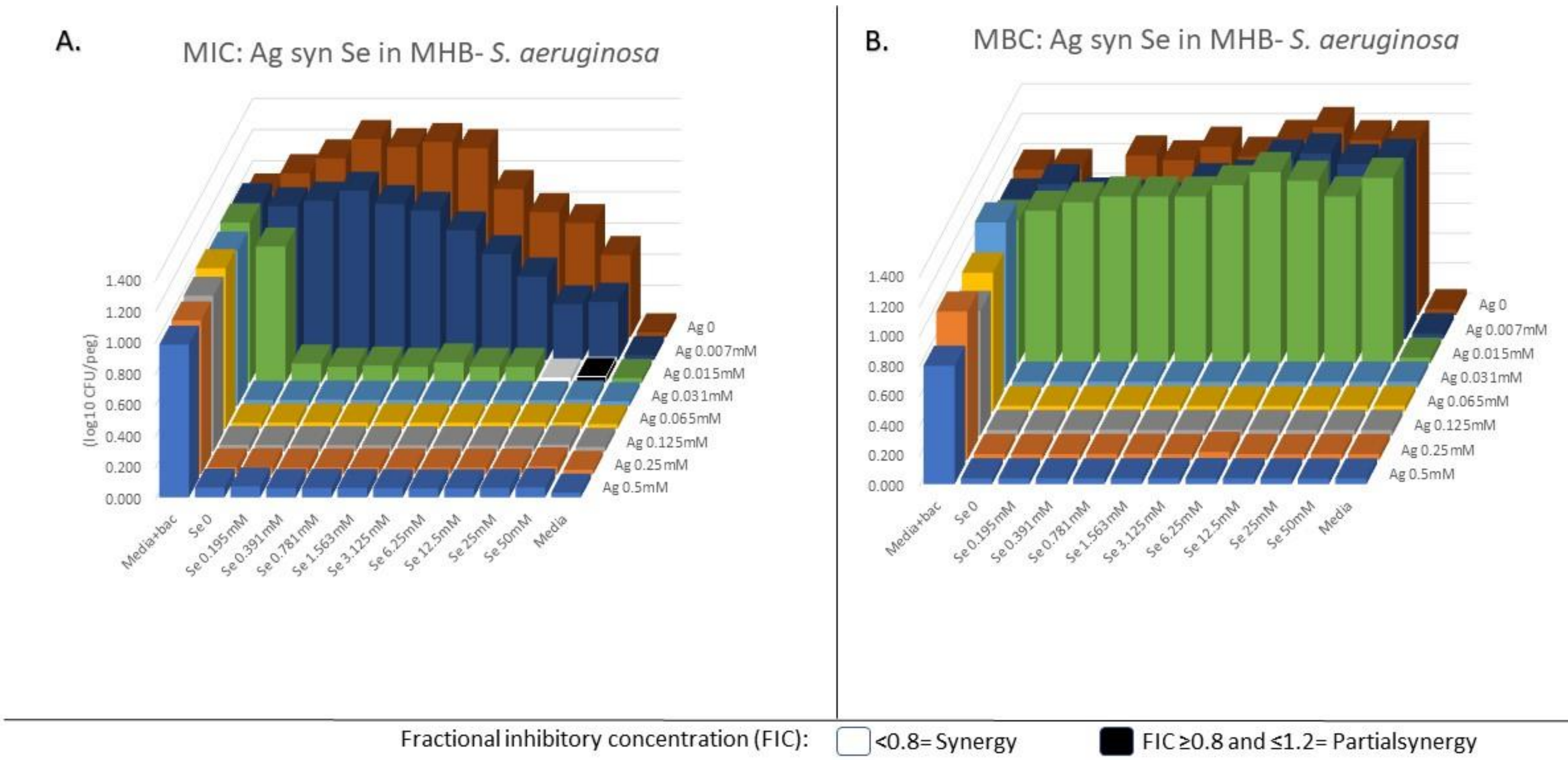

Figure S48. A. Minimum inhibitory concentration (MIC) and B. minimum bactericidal concentration (MBC) synergism (syn) effects of silver nitrate ($AgNO_3$) combined with sodium selenite (Na_2SeO_3) against *P. aeruginosa* in MHB media.

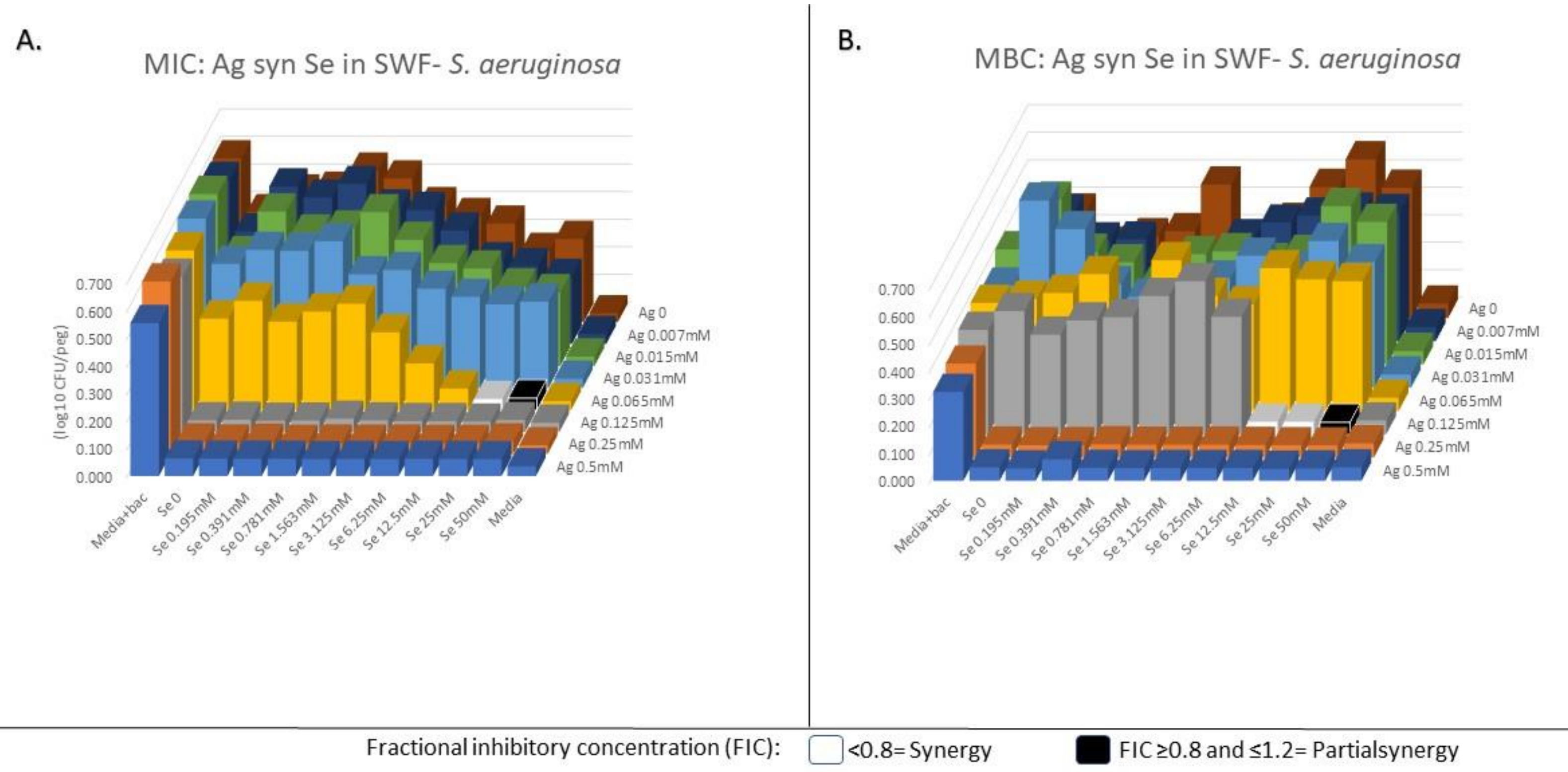

Figure S49. A. Minimum inhibitory concentration (MIC) and B. minimum bactericidal concentration (MBC) synergism (syn) effects of silver nitrate ($AgNO_3$) combined with sodium selenite (Na_2SeO_3) against *P. aeruginosa* in SWF media.

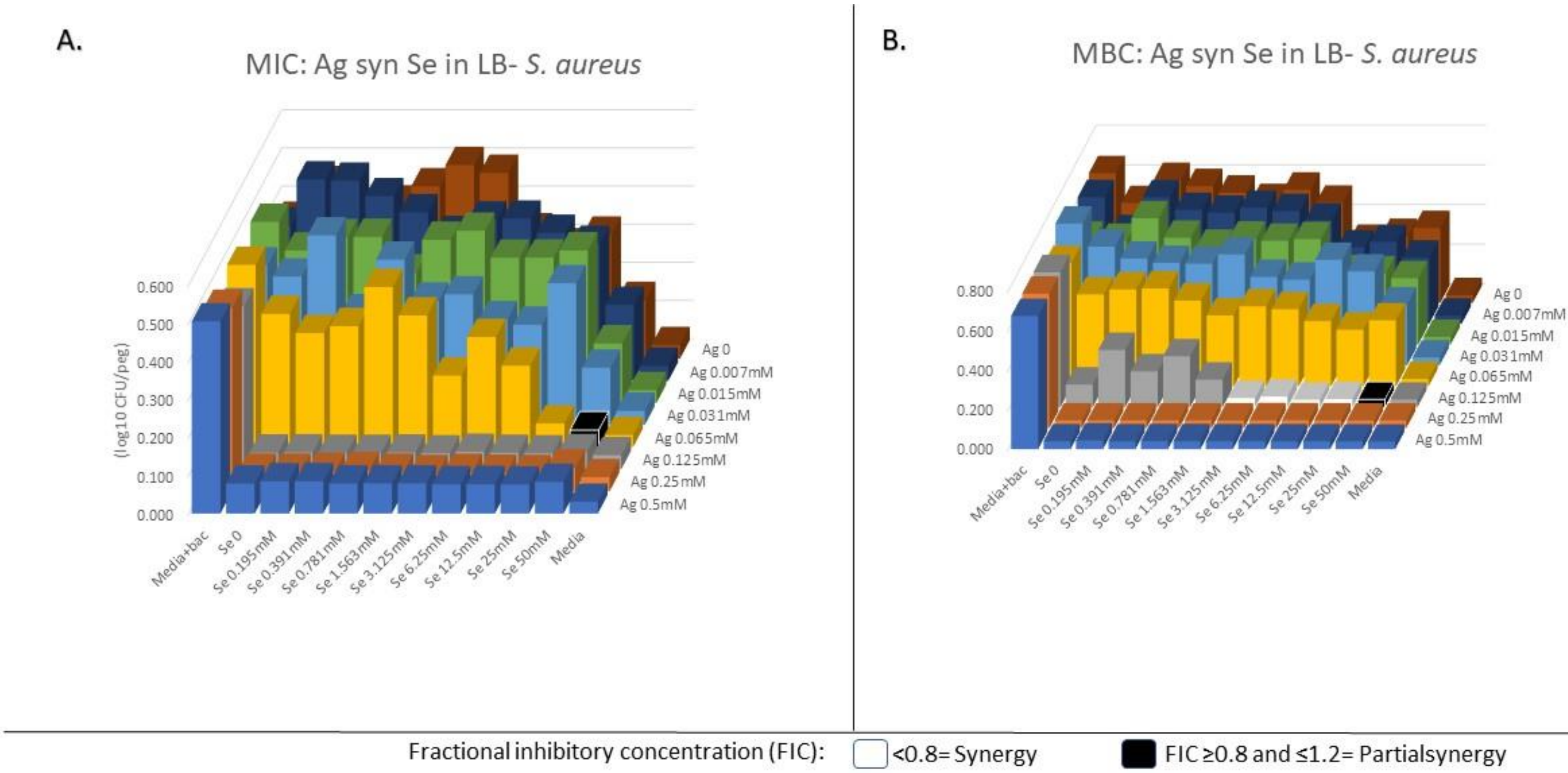

Figure S50. A. Minimum inhibitory concentration (MIC) and B. minimum bactericidal concentration (MBC) synergism (syn) effects of silver nitrate ($AgNO_3$) combined with sodium selenite (Na_2SeO_3) against *S. aureus* in LB media.

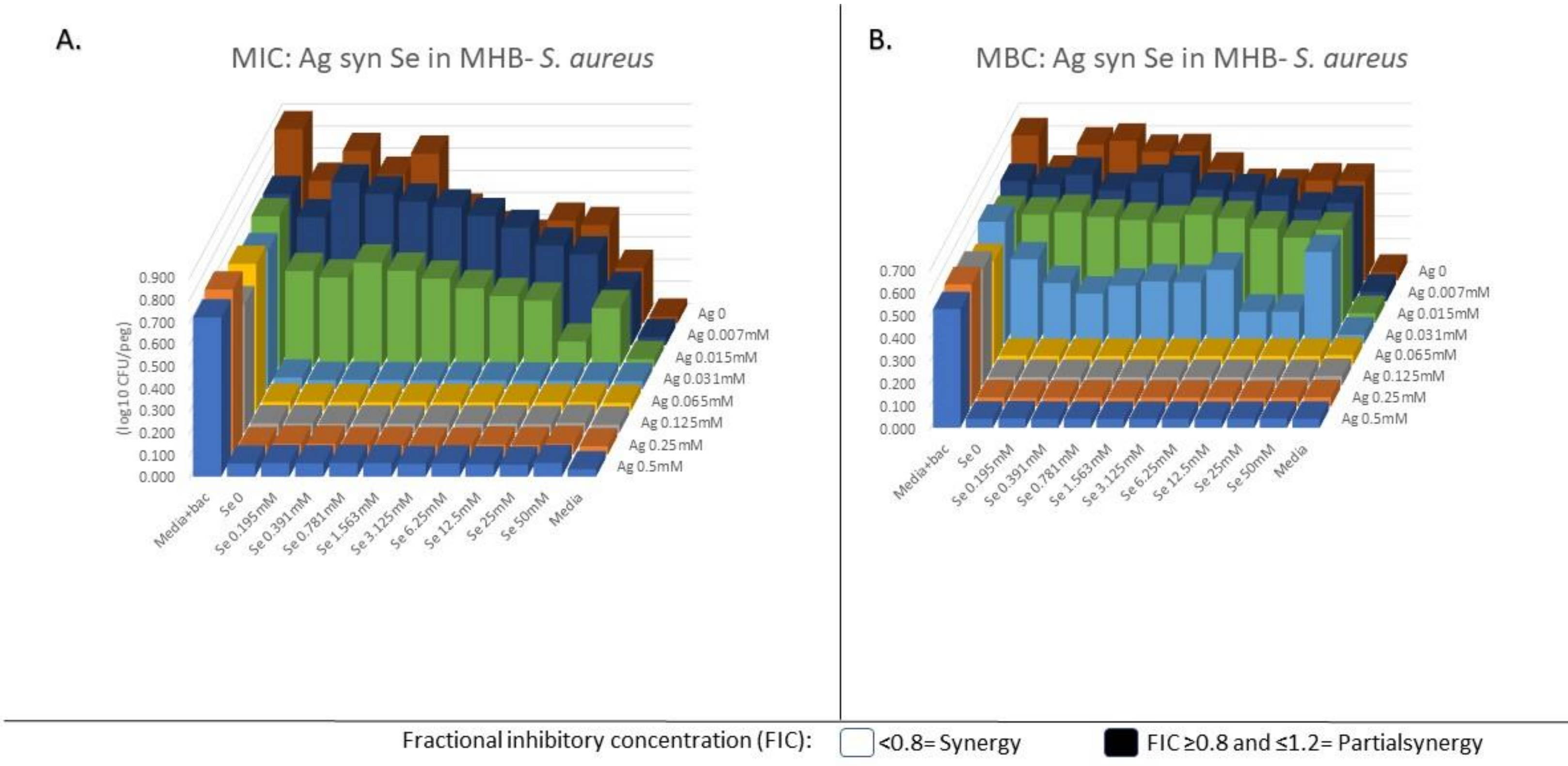

Figure S51. A. Minimum inhibitory concentration (MIC) and B. minimum bactericidal concentration (MBC) synergism (syn) effects of silver nitrate ($AgNO_3$) combined with sodium selenite (Na_2SeO_3) against *S. aureus* in MHB media.

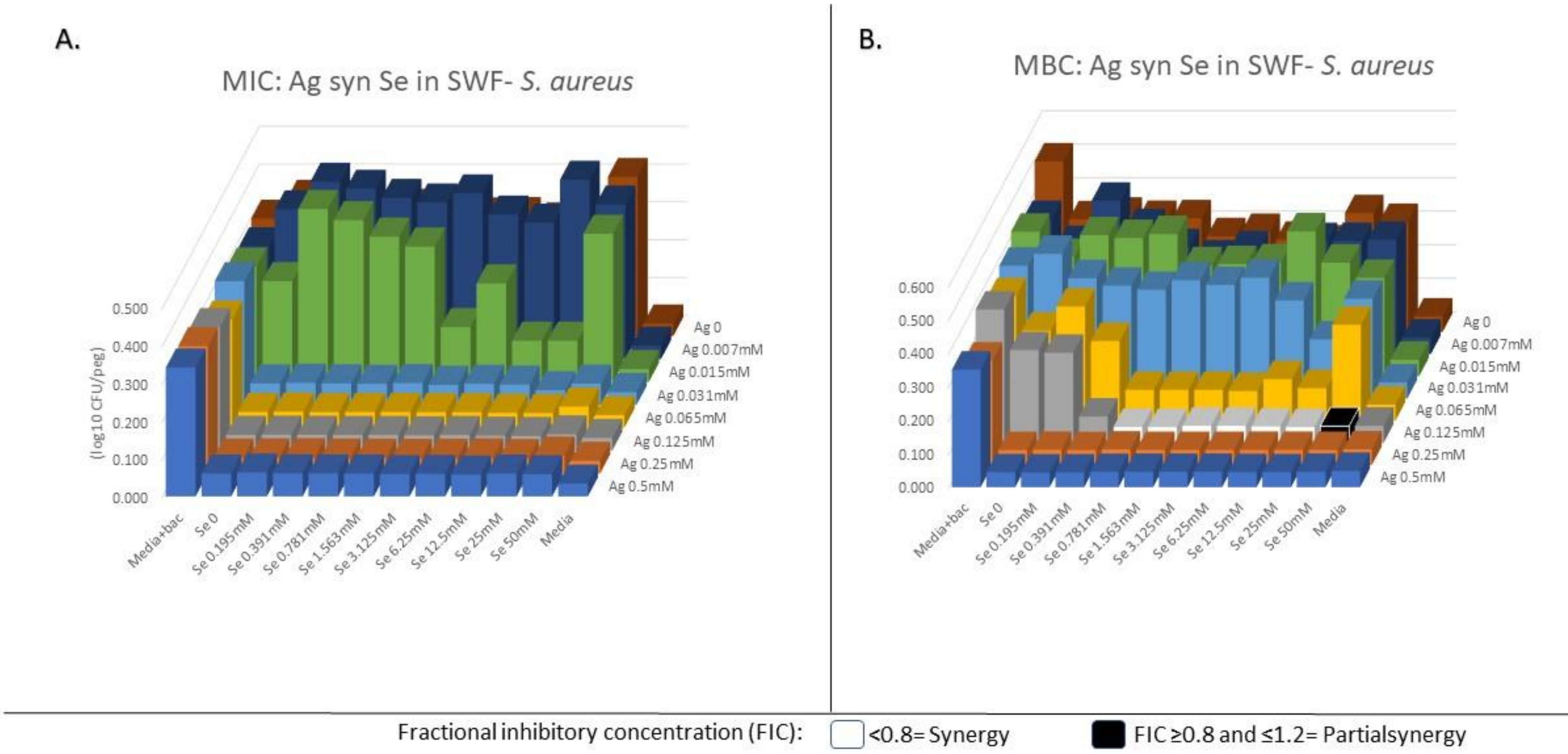

Figure S52. A. Minimum inhibitory concentration (MIC) and B. minimum bactericidal concentration (MBC) synergism (syn) effects of silver nitrate ($AgNO_3$) combined with sodium selenite (Na_2SeO_3) against *S. aureus* in SWF media.

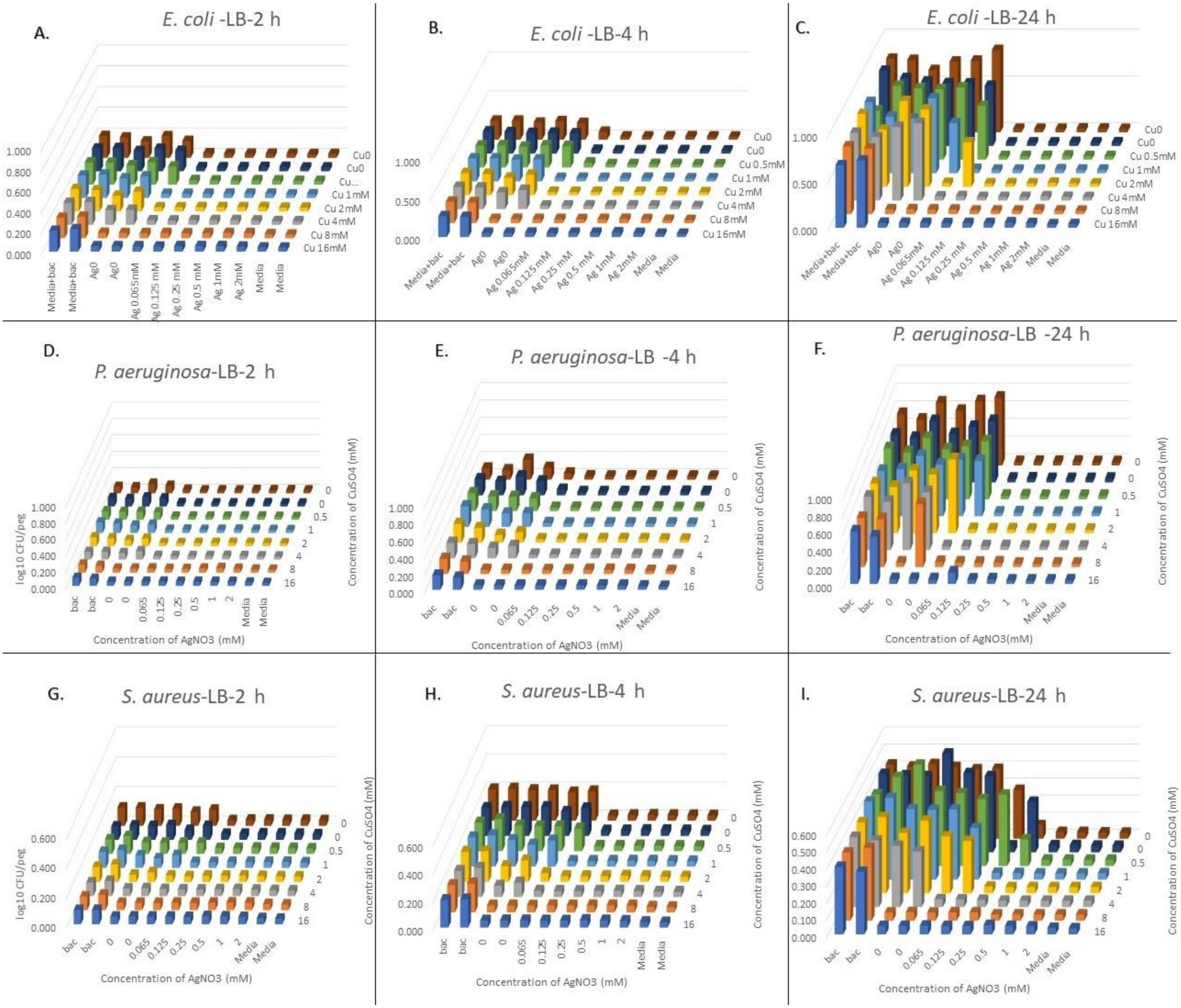
A.
E. coli -LB-2 h
B.
E. coli -LB-4 h
C.
E. coli -LB-24 h
Cu0
Cu0
Cu 0.5mM
Cu 1mM
Cu 2mM
Cu 4mM
Cu 8mM
Cu 16mM
Media+bac
Media+bac
Ag0
Ag0
Ag 0.065mM
Ag 0.125 mM
Ag 0.25 mM
Ag 0.5 mM
Ag 1mM
Ag 2mM
Media
Media
D.
P. aeruginosa-LB-2 h
E.
P. aeruginosa-LB -4 h
F.
P. aeruginosa-LB -24 h
log10 CFU/peg
Concentration of CuSO4 (mM)
Concentration of AgNO3 (mM)
G.
S. aureus-LB-2 h
H.
S. aureus-LB-4 h
I.
S. aureus-LB-24 h
bac
bac
0
0
0.065
0.125
0.25
0.5
1
2
Media
Media
0
0
0.5
1
2
4
8
16

Figure S53. Recovery potency of *E. coli* ATCC 259 against synergism effects of silver nitrate ($AgNO_3$) combined with copper (II) sulfate ($CuSO_4$) after 2 h (A), 4 h (B), and 24 h (C); *P. aeruginosa* after 2 h (D), 4 h (E), and 24 h (F); and *S. aureus* after 2 h (G), 4 h (H), and 24 h (I) in LB..

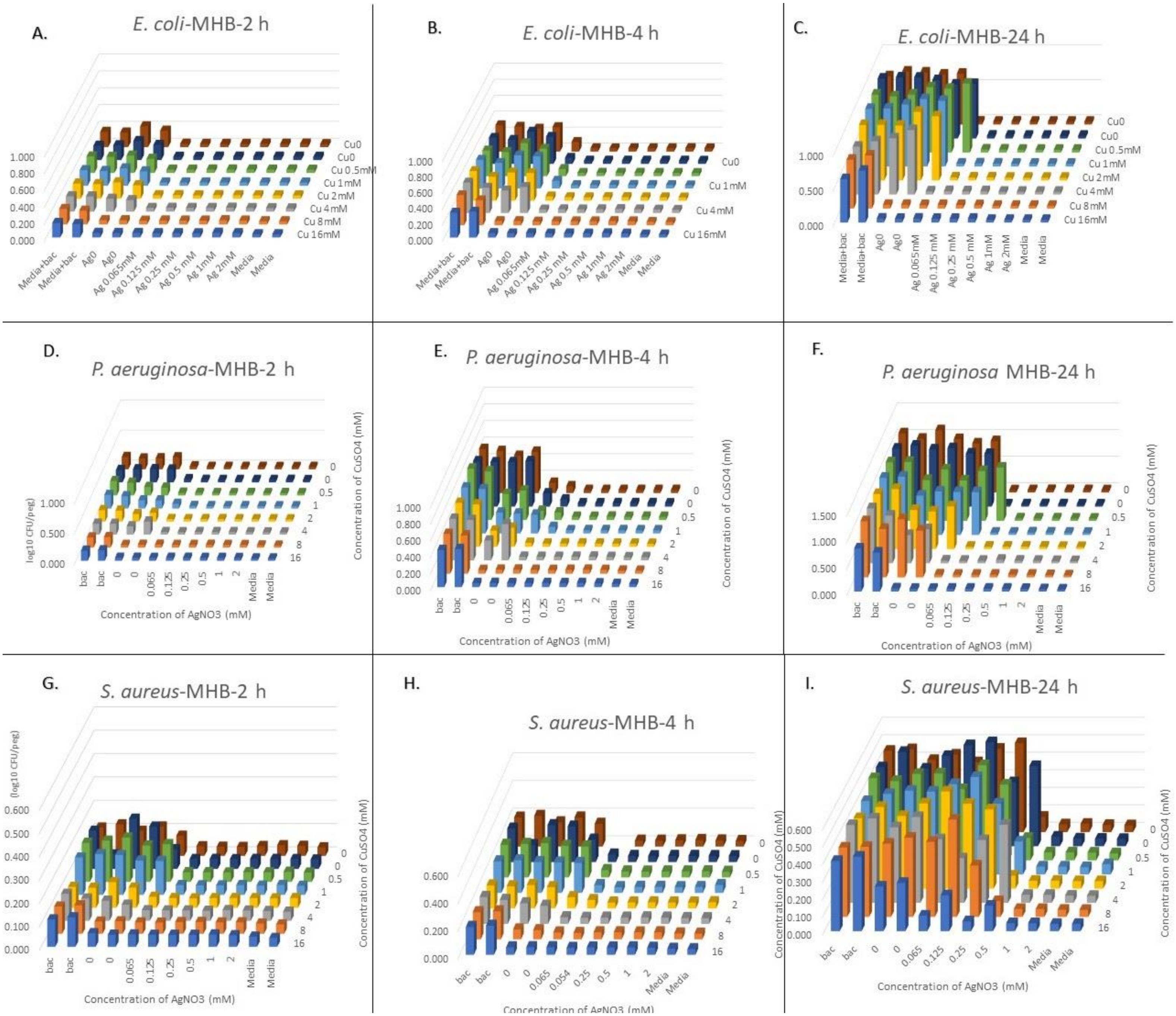

Figure S54. Recovery potency of *E. coli* ATCC 259 against synergism effects of silver nitrate ($AgNO_3$) combined with copper (II) sulfate ($CuSO_4$) after 2 h (A), 4 h (B), and 24 h (C); *P. aeruginosa* after 2 h

(D), 4 h (E), and 24 h (F); and *S. aureus* after 2 h (G), 4 h (H), and 24 h (I) in Mueller–Hinton broth (MHB).

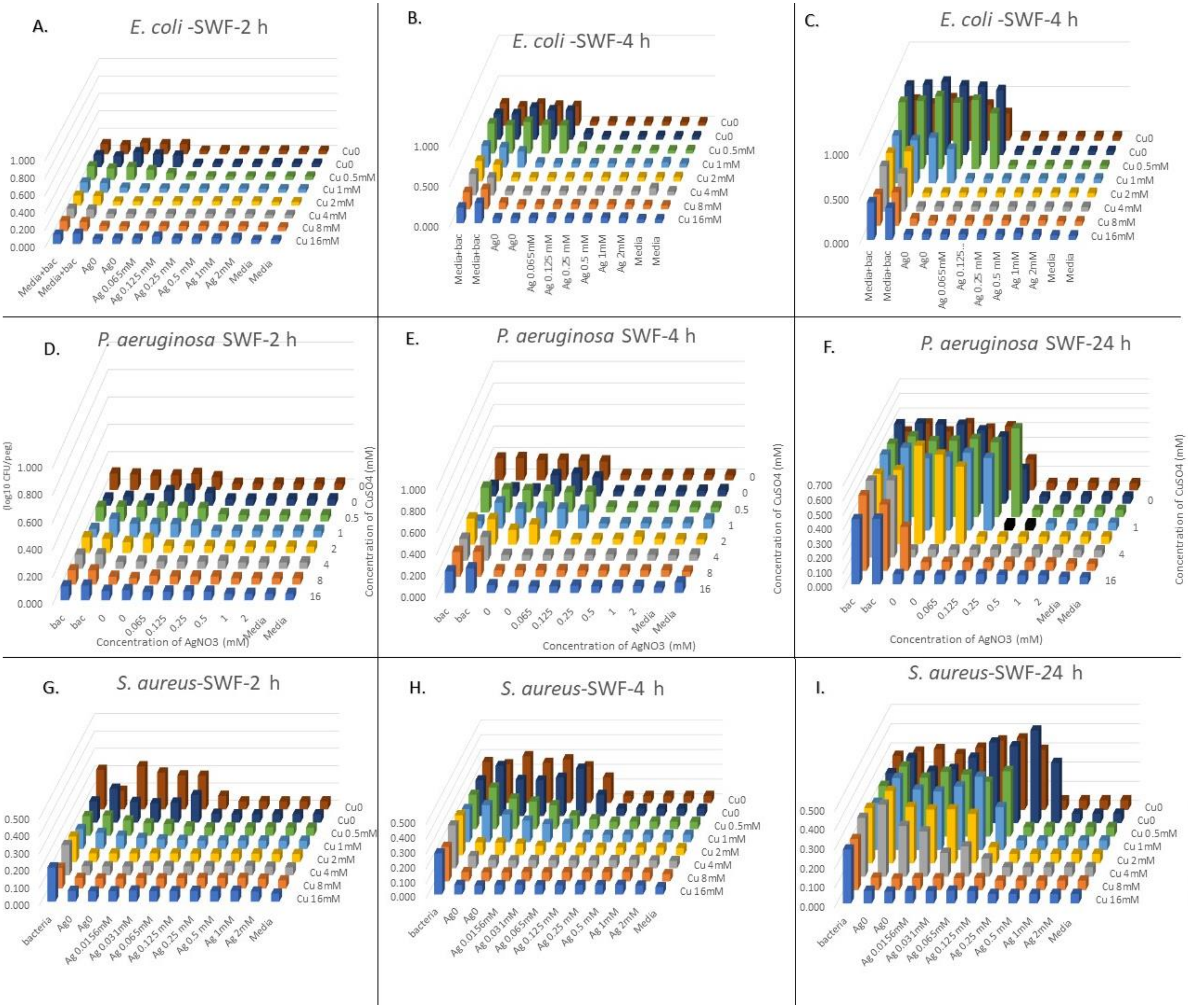

Figure S55. Recovery potency of *E. coli* ATCC 259 against synergism effects of silver nitrate ($AgNO_3$) combined with copper (II) sulfate ($CuSO_4$) after 2 h (A), 4 h (B), and 24 h (C); *P. aeruginosa* after 2 h

(D), 4 h (E), and 24 h (F); and *S. aureus* after 2 h (G), 4 h (H), and 24 h (I) in simulated wound fluid (SWF).

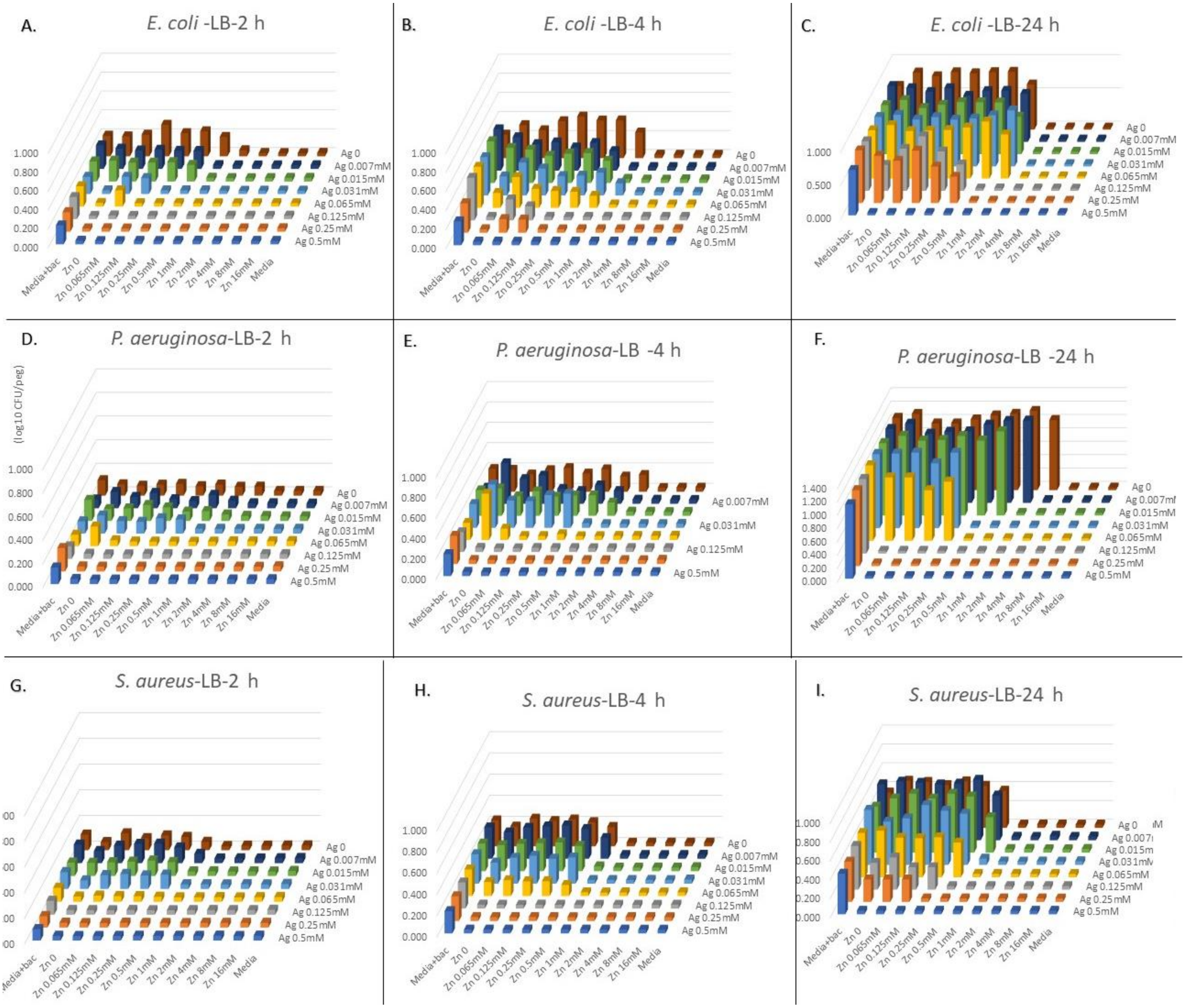

Figure S56. Recovery potency of *E. coli* ATCC 259 against synergism effects of silver nitrate ($AgNO_3$) combined with zinc sulfate ($ZnSO_4$) after 2 h (A), 4 h (B), and 24 h (C); *P. aeruginosa* after 2 h (D), 4 h (E), and 24 h (F); and *S. aureus* after 2 h (G), 4 h (H), and 24 h (I) in LB..

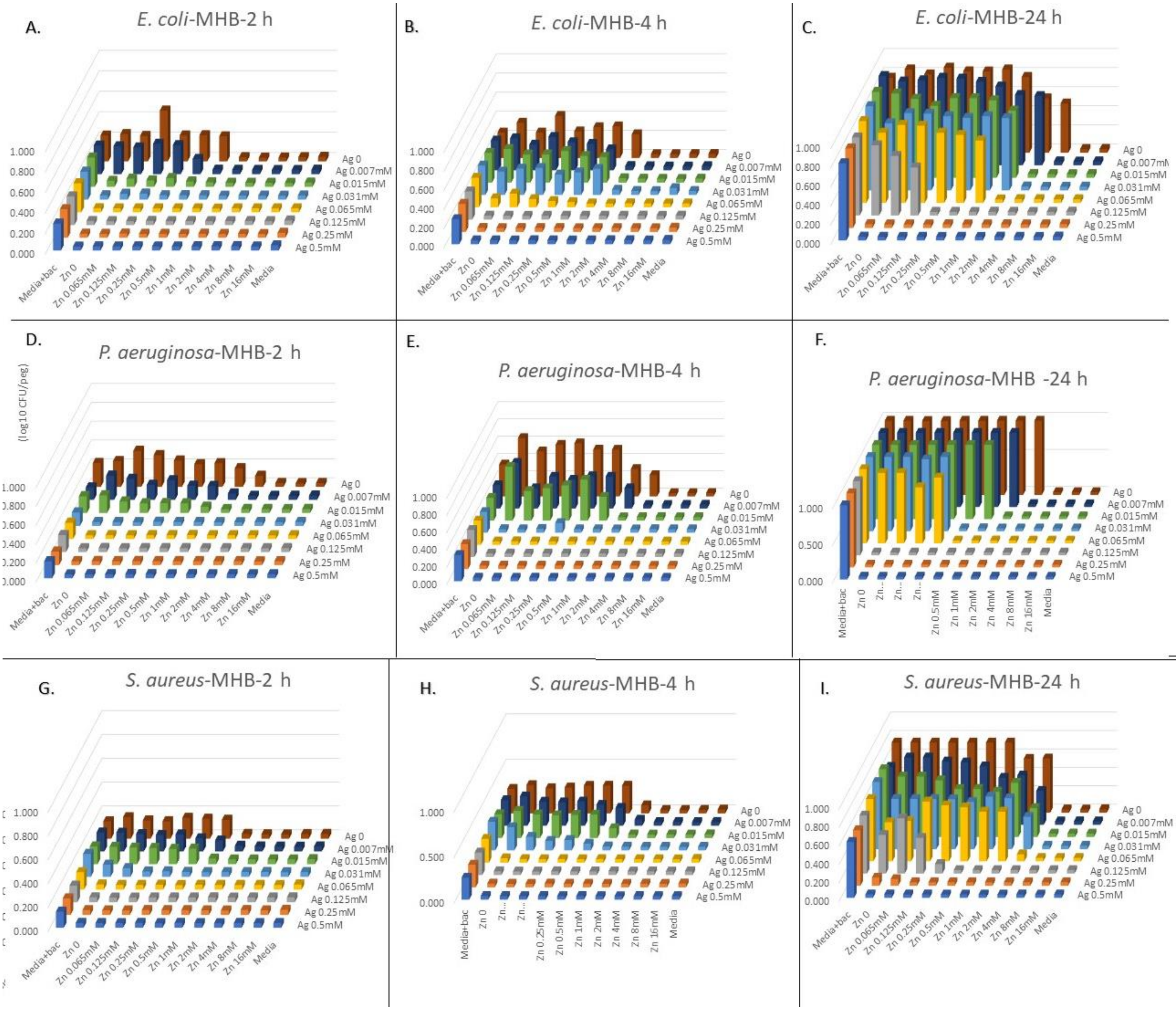

Figure S57. Recovery potency of *E. coli* ATCC 259 against synergism effects of silver nitrate ($AgNO_3$) combined with zinc sulfate ($ZnSO_4$) after 2 h (A), 4 h (B), and 24 h (C); *P. aeruginosa* after 2 h (D), 4 h (E), and 24 h (F); and *S. aureus* after 2 h (G), 4 h (H), and 24 h (I) in MHB**.**

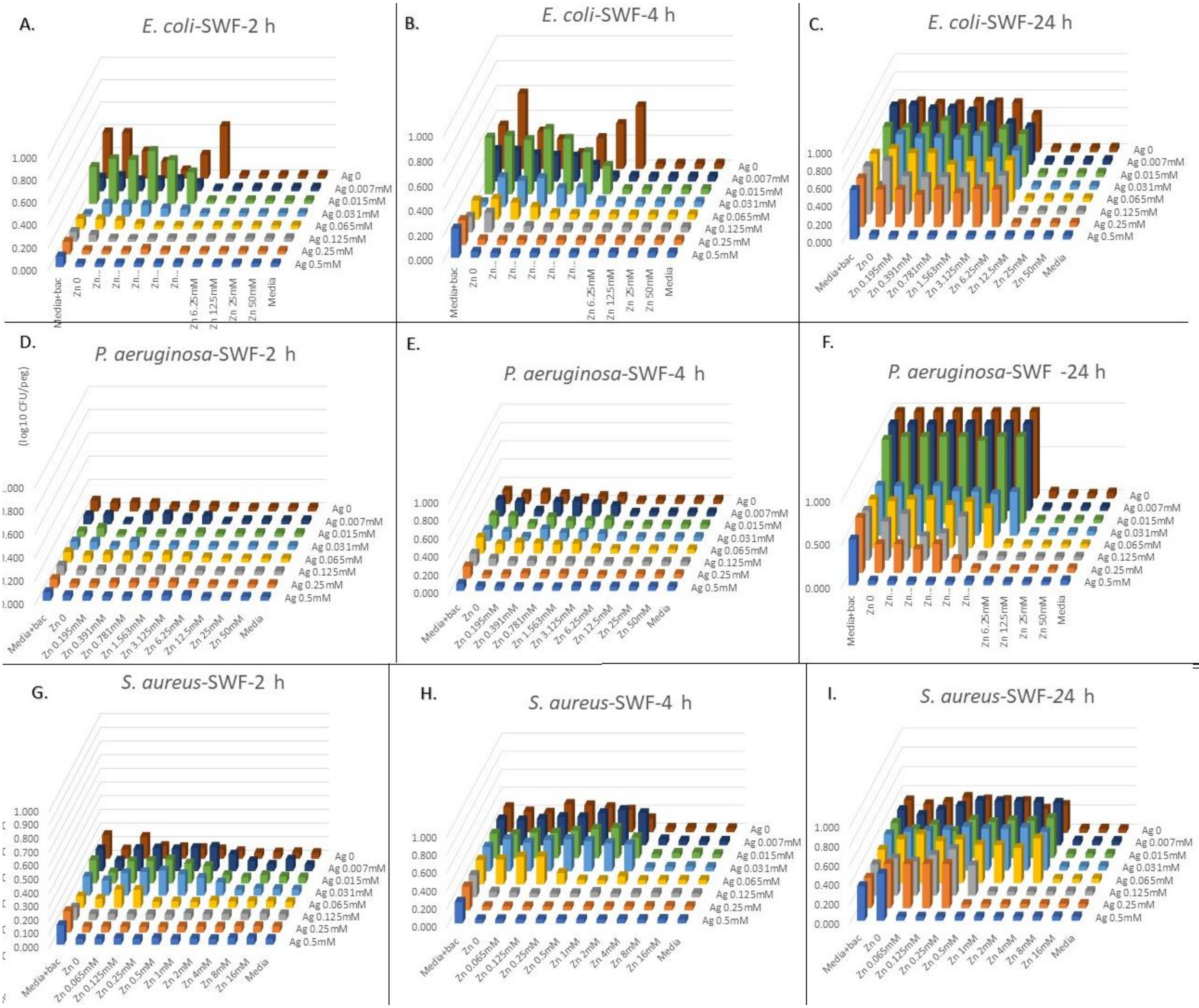

Figure S58. Recovery potency of *E. coli* ATCC 259 against synergism effects of silver nitrate ($AgNO_3$) combined with zinc sulfate ($ZnSO_4$) after 2 h (A), 4 h (B), and 24 h (C); *P. aeruginosa* after 2 h (D), 4 h (E), and 24 h (F); and *S. aureus* after 2 h (G), 4 h (H), and 24 h (I) in SWF**.**

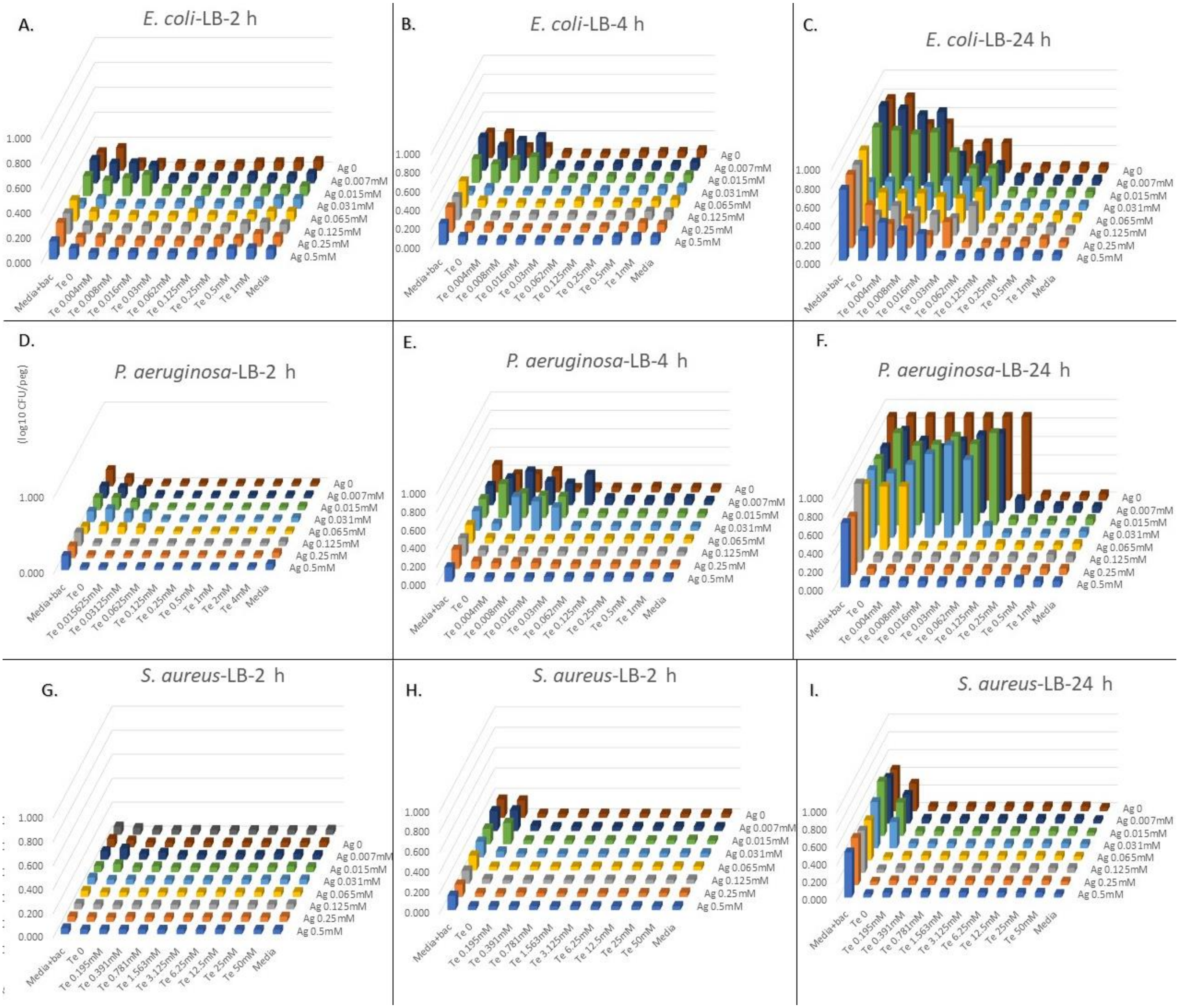

Figure S59. Recovery potency of *E. coli* ATCC 259 against synergism effects of silver nitrate ($AgNO_3$) combined with tellurite (K_2TeO_3) after 2 h (A), 4 h (B), and 24 h (C); *P. aeruginosa* after 2 h (D), 4 h (E), and 24 h (F); and *S. aureus* after 2 h (G), 4 h (H), and 24 h (I) in LB.

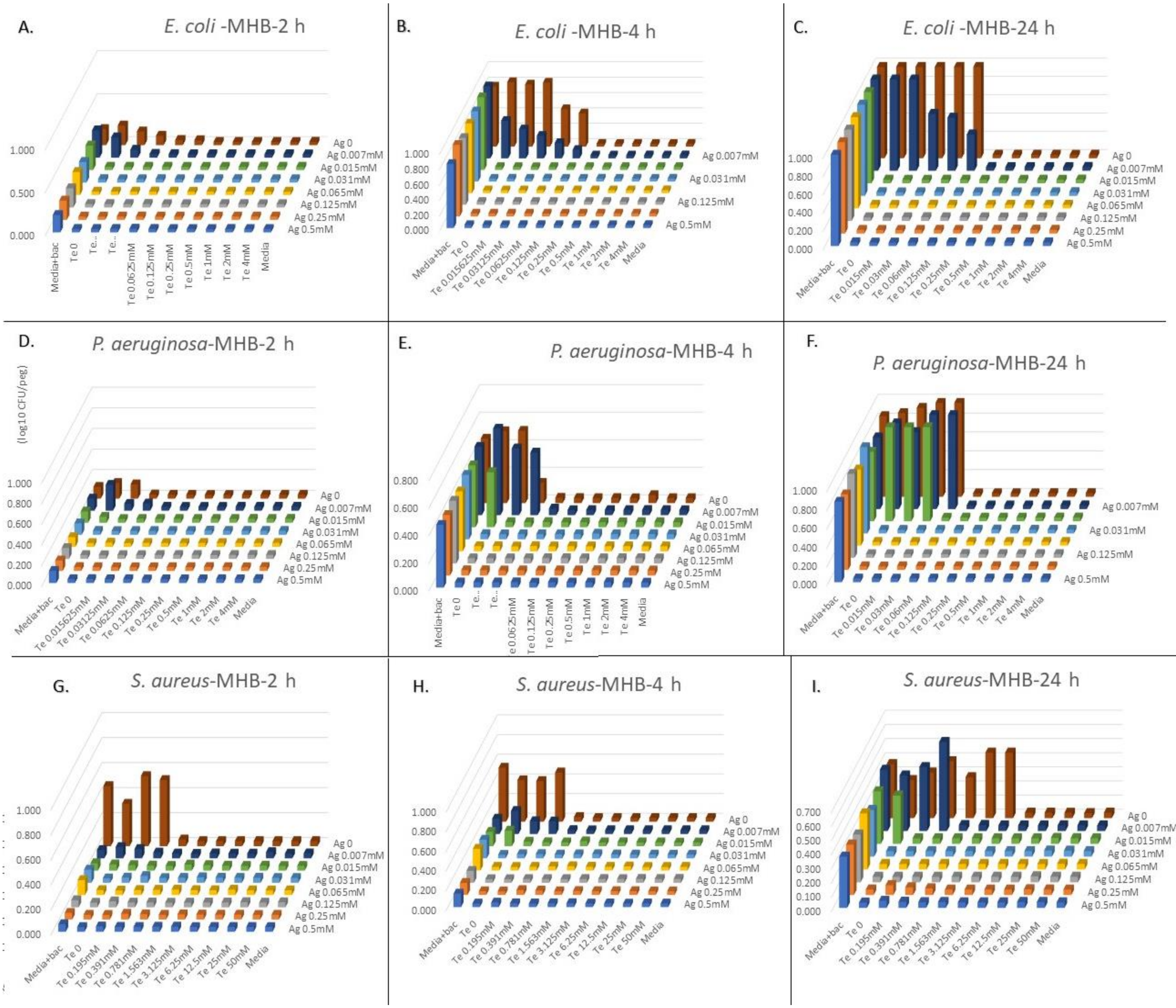

Figure S60. Recovery potency of *E. coli* ATCC 259 against synergism effects of silver nitrate ($AgNO_3$) combined with tellurite (K_2TeO_3) after 2 h (A), 4 h (B), and 24 h (C); *P. aeruginosa* after 2 h (D), 4 h (E), and 24 h (F); and *S. aureus* after 2 h (G), 4 h (H), and 24 h (I) in MHB.

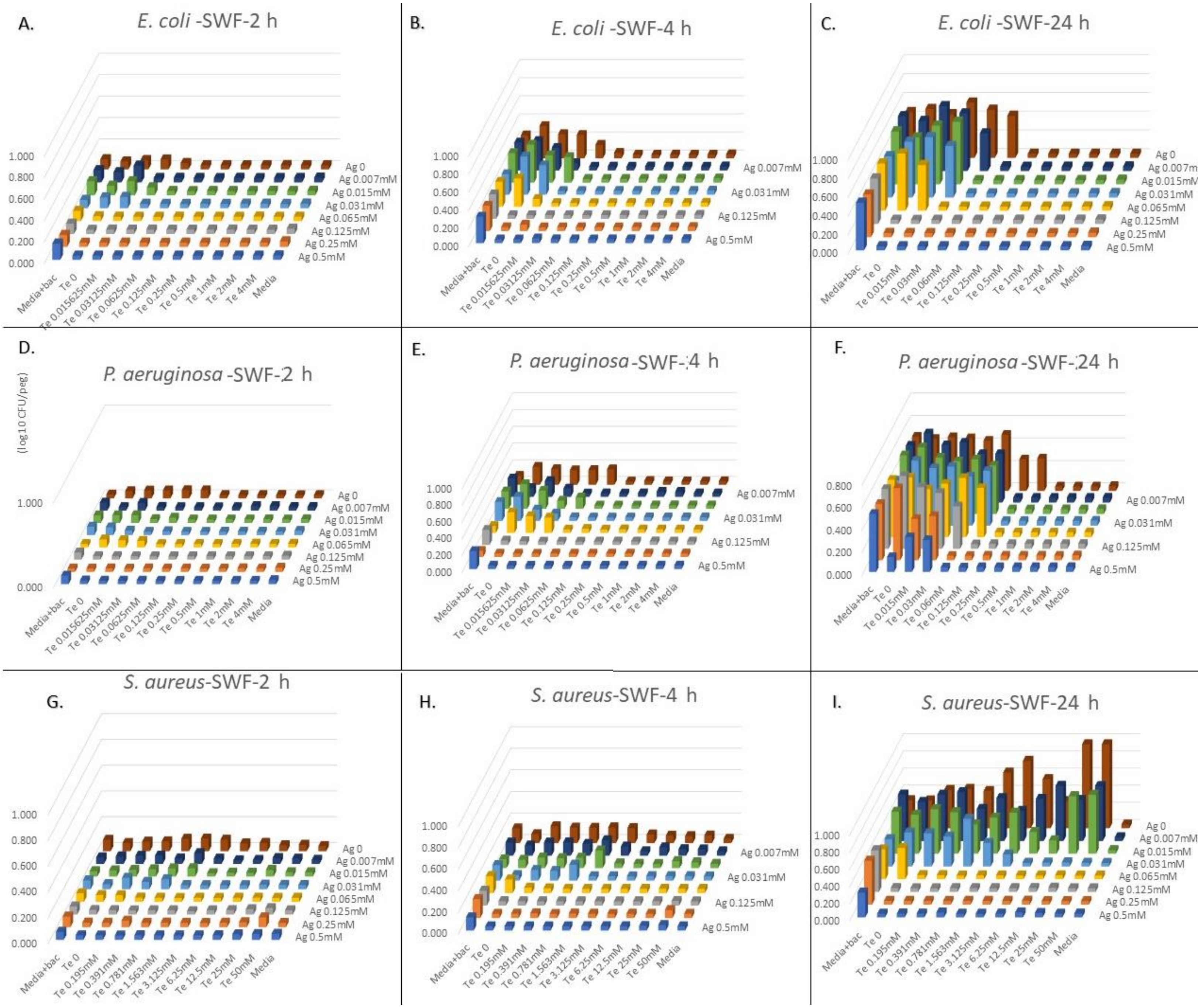

Figure S61. Recovery potency of *E. coli* ATCC 259 against synergism effects of silver nitrate ($AgNO_3$) combined with tellurite (K_2TeO_3) after 2 h (A), 4 h (B), and 24 h (C); *P. aeruginosa* after 2 h (D), 4 h (E), and 24 h (F); and *S. aureus* after 2 h (G), 4 h (H), and 24 h (I) in SWF.

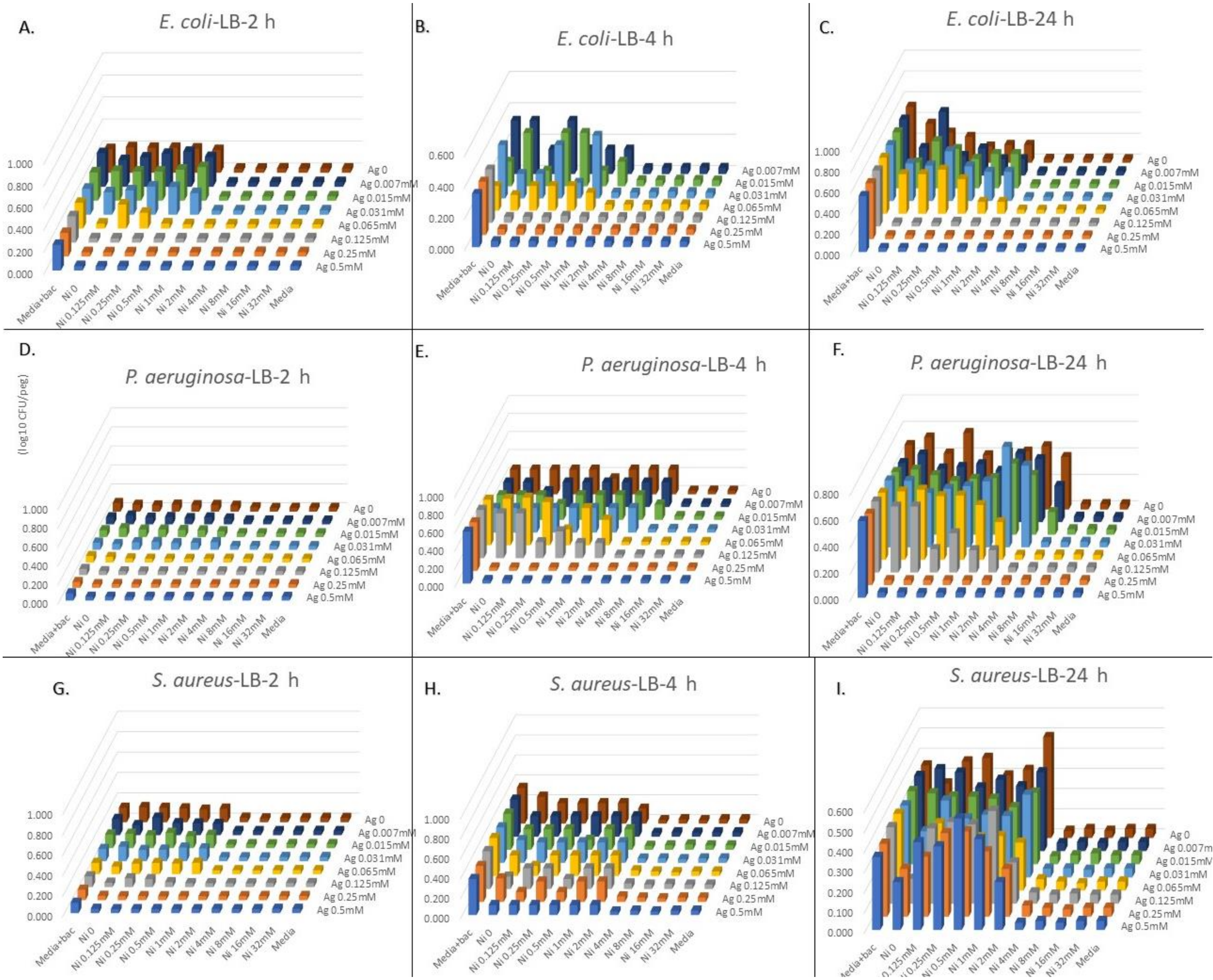

Figure S62. Recovery potency of *E. coli* ATCC 259 against synergism effects of silver nitrate ($AgNO_3$) combined with nickel sulfate ($NiSO_4$) after 2 h (A), 4 h (B), and 24 h (C); *P. aeruginosa* after 2 h (D), 4 h (E), and 24 h (F); and *S. aureus* after 2 h (G), 4 h (H), and 24 h (I) in LB.

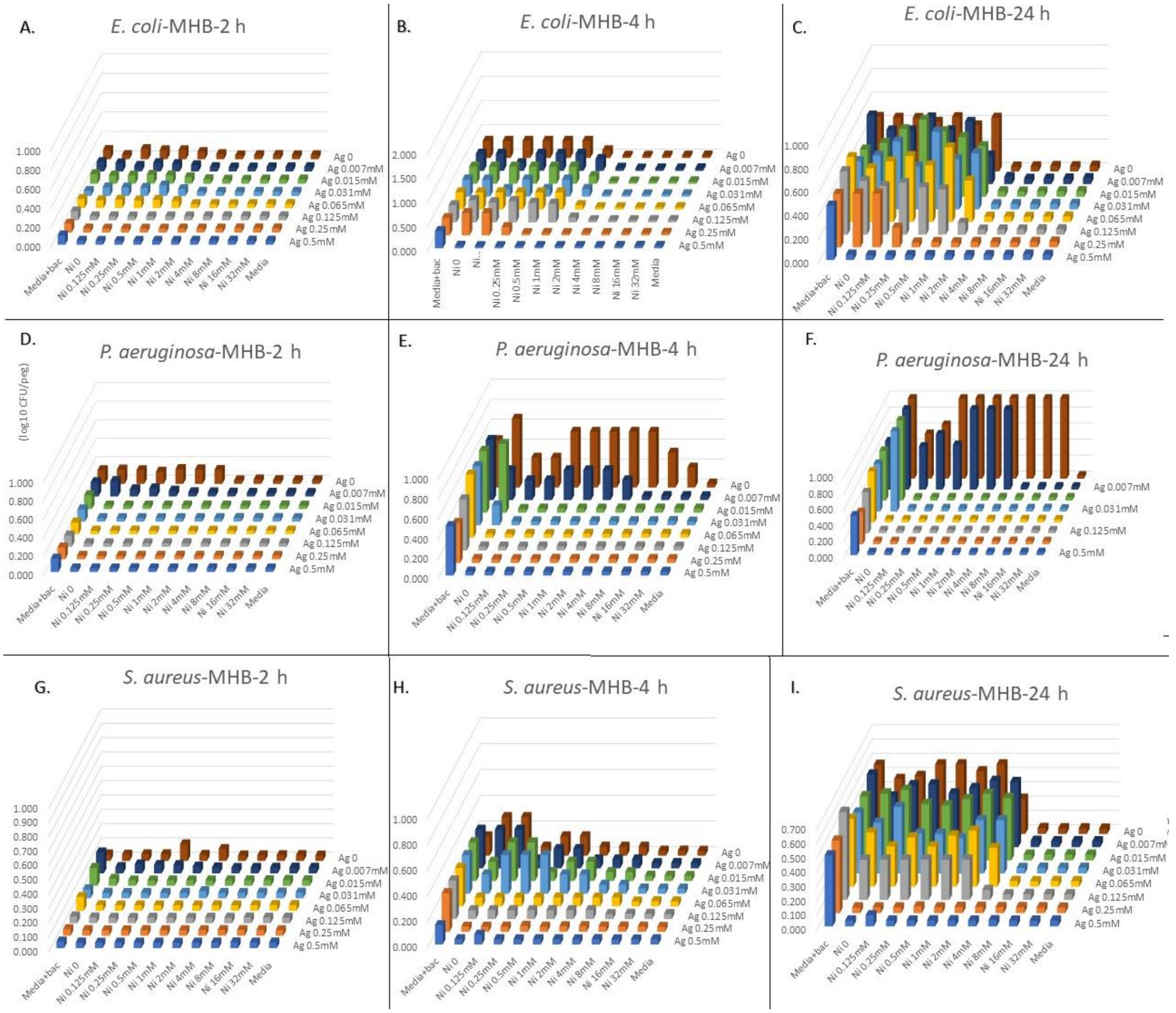

Figure S63. Recovery potency of *E. coli* ATCC 259 against synergism effects of silver nitrate ($AgNO_3$) combined with nickel sulfate ($NiSO_4$) after 2 h (A), 4 h (B), and 24 h (C); *P. aeruginosa* after 2 h (D), 4 h (E), and 24 h (F); and *S. aureus* after 2 h (G), 4 h (H), and 24 h (I) in MHB.

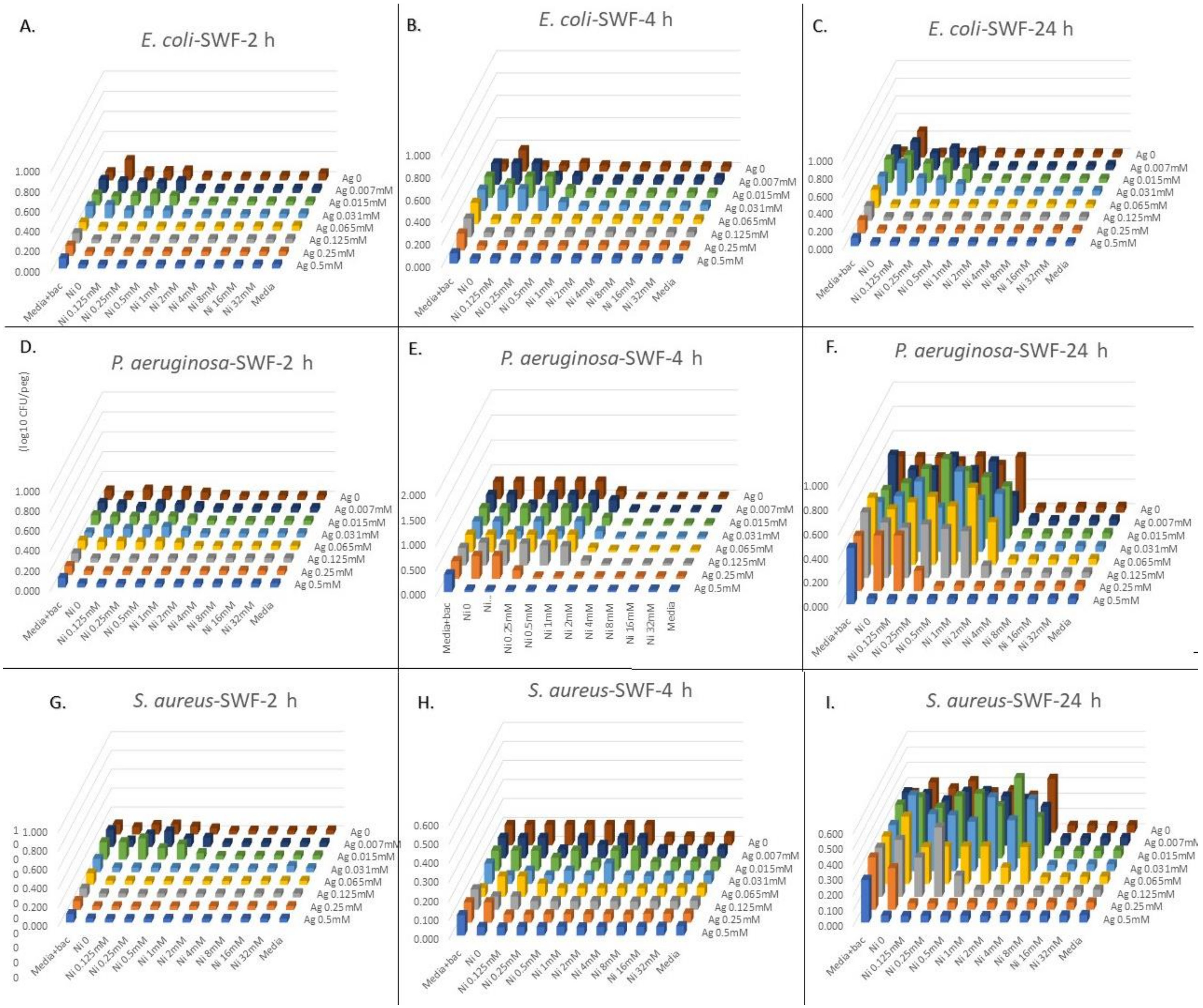
A.
E. coli-SWF-2 h
B.
E. coli-SWF-4 h
C.
E. coli-SWF-24 h
D.
P. aeruginosa-SWF-2 h
(log10 CFU/peg)
E.
P. aeruginosa-SWF-4 h
F.
P. aeruginosa-SWF-24 h
G.
S. aureus-SWF-2 h
H.
S. aureus-SWF-4 h
I.
S. aureus-SWF-24 h
Media+bac
Ni 0
Ni 0.125mM
Ni 0.25mM
Ni 0.5mM
Ni 1mM
Ni 2mM
Ni 4mM
Ni 8mM
Ni 16mM
Ni 32mM
Media
Ag 0
Ag 0.007mM
Ag 0.015mM
Ag 0.031mM
Ag 0.065mM
Ag 0.125mM
Ag 0.25mM
Ag 0.5mM

Figure S64. Recovery potency of *E. coli* ATCC 259 against synergism effects of silver nitrate ($AgNO_3$) combined with sodium selenite (Na_2SeO_3) after 2 h (A), 4 h (B), and 24 h (C); *P. aeruginosa* after 2 h (D), 4 h (E), and 24 h (F); and *S. aureus* after 2 h (G), 4 h (H), and 24 h (I) in SWF.

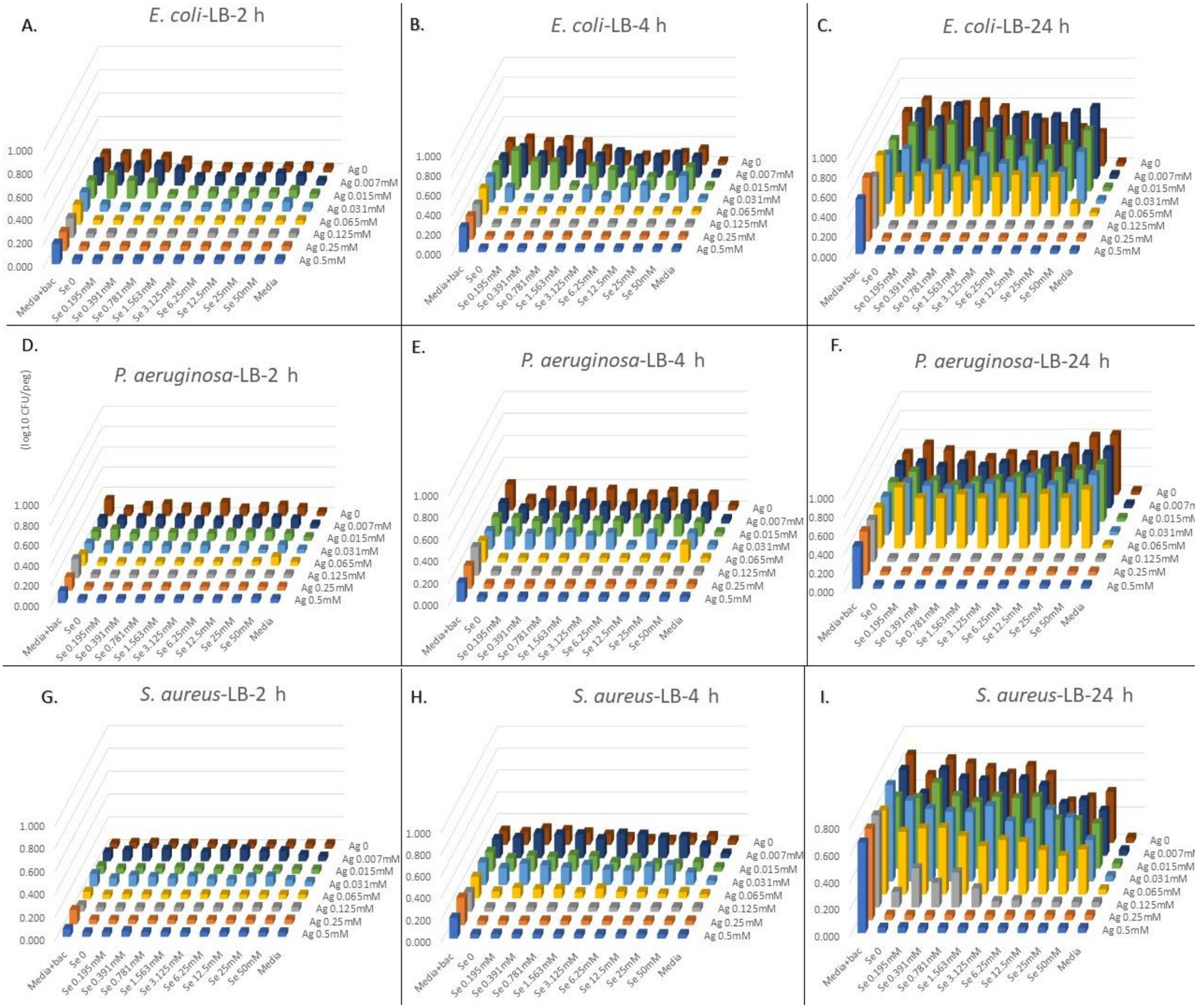

Figure S65. Recovery potency of *E. coli* ATCC 259 against synergism effects of silver nitrate ($AgNO_3$) combined with sodium selenite (Na_2SeO_3) after 2 h (A), 4 h (B), and 24 h (C); *P. aeruginosa* after 2 h (D), 4 h (E), and 24 h (F); and *S. aureus* after 2 h (G), 4 h (H), and 24 h (I) in LB.

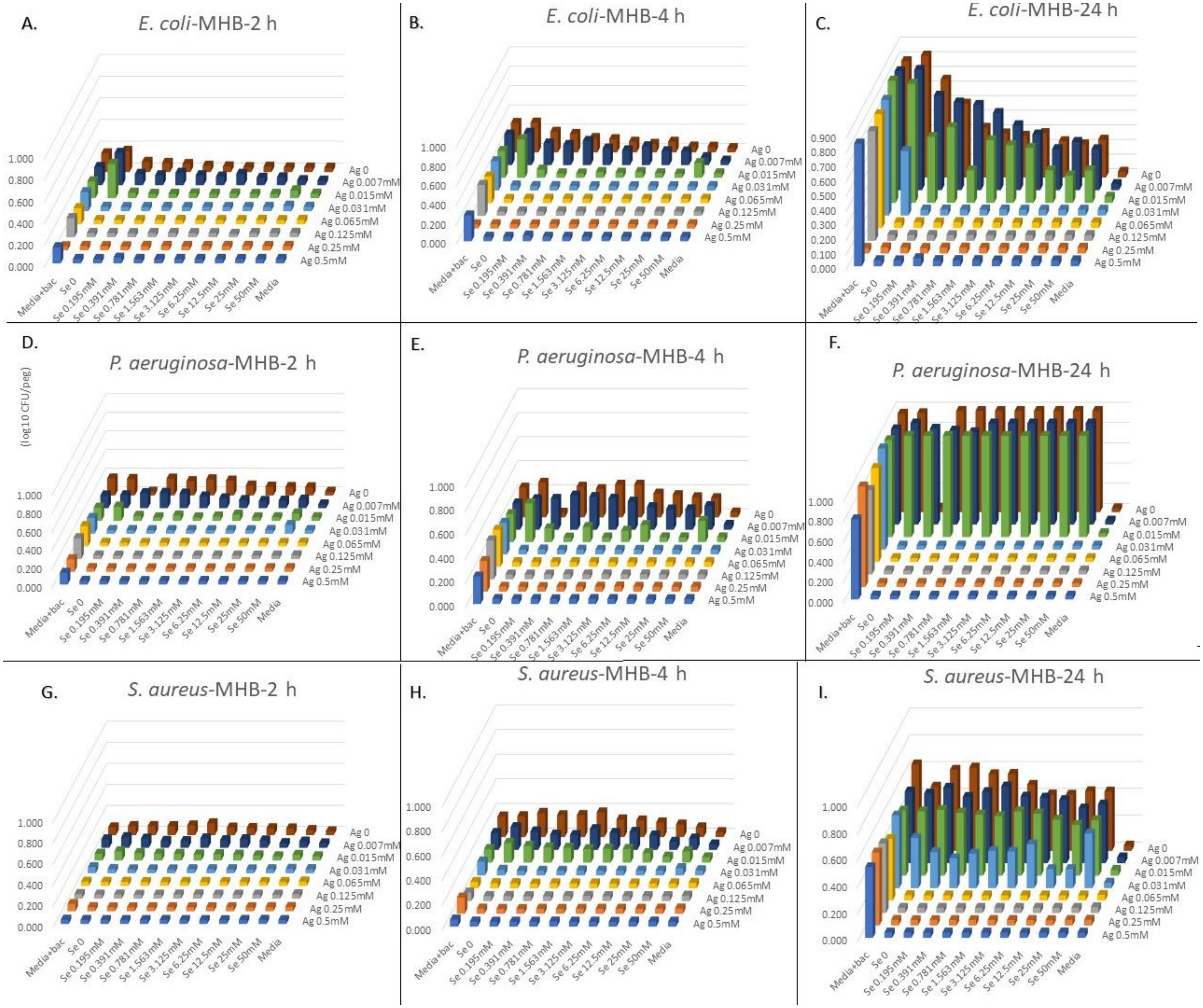

Figure S66. Recovery potency of *E. coli* ATCC 259 against synergism effects of silver nitrate ($AgNO_3$) combined with sodium selenite (Na_2SeO_3) after 2 h (A), 4 h (B), and 24 h (C); *P. aeruginosa* after 2 h (D), 4 h (E), and 24 h (F); and *S. aureus* after 2 h (G), 4 h (H), and 24 h (I) in MHB.

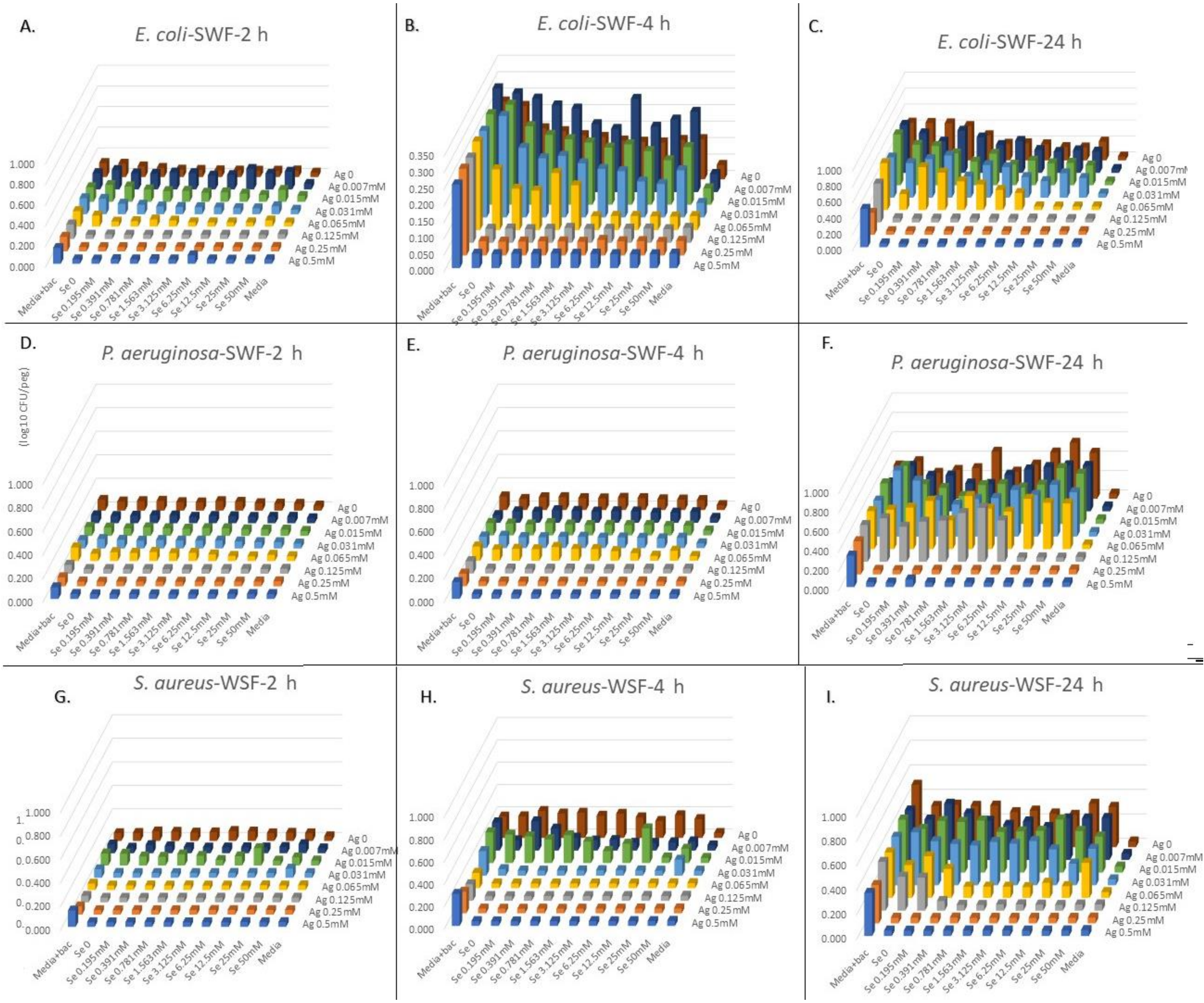

Figure S67. Recovery potency of *E. coli* ATCC 259 against synergism effects of silver nitrate ($AgNO_3$) combined with sodium selenite (Na_2SeO_3) after 2 h (A), 4 h (B), and 24 h (C); *P. aeruginosa* after 2 h (D), 4 h (E), and 24 h (F); and *S. aureus* after 2 h (G), 4 h (H), and 24 h (I) in SWF.

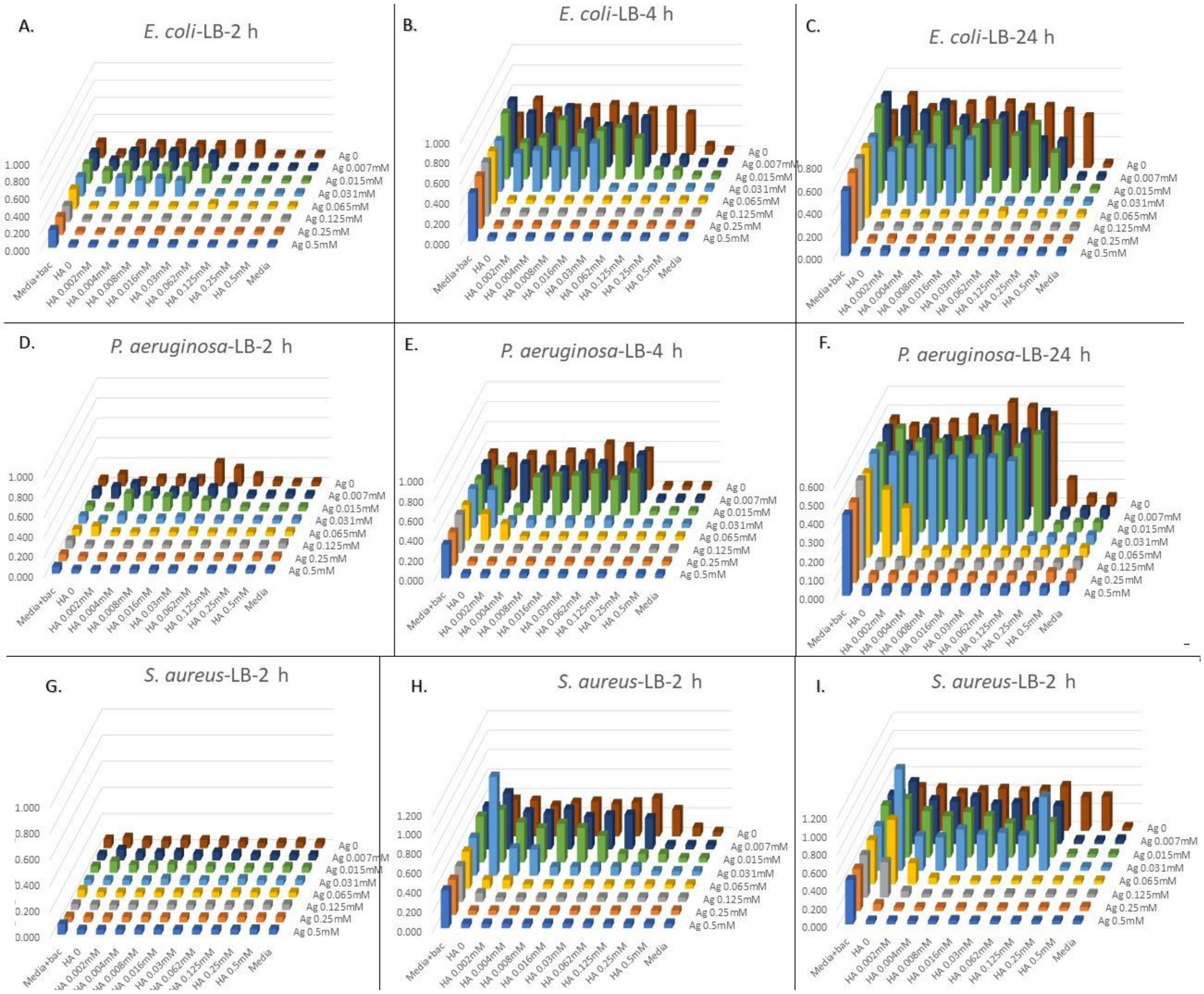

Figure S68. Recovery potency of *E. coli* ATCC 259 against synergism effects of silver nitrate ($AgNO_3$) combined with tetrachloroaurate (iii) (HA, $AuCl_4$) after 2 h (A), 4 h (B), and 24 h (C); *P. aeruginosa* after 2 h (D), 4 h (E), and 24 h (F); and *S. aureus* after 2 h (G), 4 h (H), and 24 h (I) in LB.

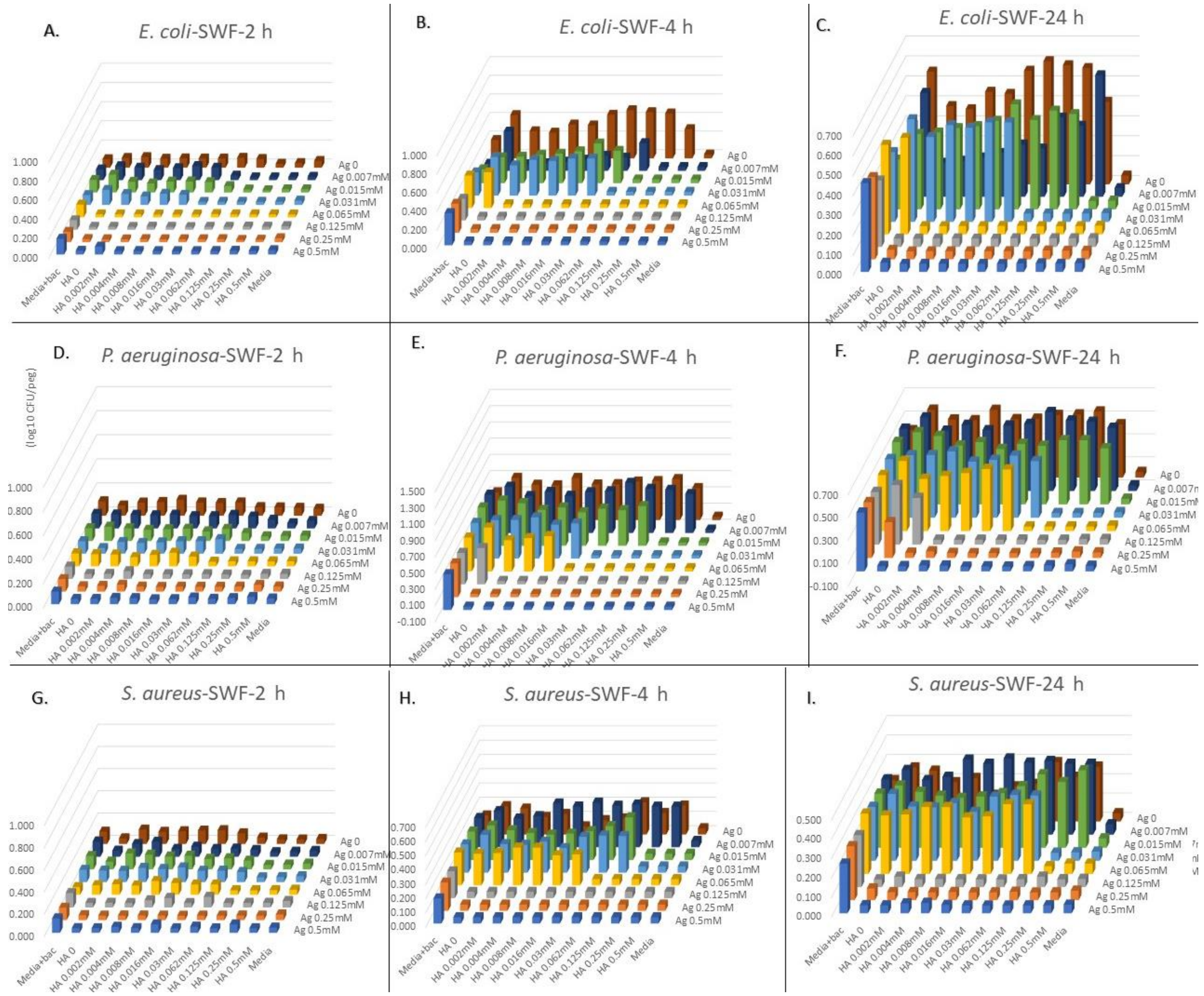
A.
E. coli-SWF-2 h
B.
E. coli-SWF-4 h
C.
E. coli-SWF-24 h
D.
P. aeruginosa-SWF-2 h
(log10 CFU/peg)
E.
P. aeruginosa-SWF-4 h
F.
P. aeruginosa-SWF-24 h
G.
S. aureus-SWF-2 h
H.
S. aureus-SWF-4 h
I.
S. aureus-SWF-24 h

Figure S69. Recovery potency of *E. coli* ATCC 259 against synergism effects of silver nitrate ($AgNO_3$) combined with tetrachloroaurate (iii) (HA, $AuCl_4$) after 2 h (A), 4 h (B), and 24 h (C); *P. aeruginosa* after 2 h (D), 4 h (E), and 24 h (F); and *S. aureus* after 2 h (G), 4 h (H), and 24 h (I) in SWF.
